# Clinics Optimizing MEthadone Take-homes for opioid use disorder (COMET): Protocol for a stepped-wedge randomized trial to facilitate clinic level changes

**DOI:** 10.1371/journal.pone.0286859

**Published:** 2023-06-09

**Authors:** Sugy Choi, Megan A. O’Grady, Charles M. Cleland, Elizabeth Knopf, Sueun Hong, Thomas D’Aunno, Yuhua Bao, Kelly S. Ramsey, Charles J. Neighbors

**Affiliations:** 1 Department of Population Health, New York University Grossman School of Medicine, New York City, NY, United States of America; 2 Department of Public Health Sciences, University of Connecticut School of Medicine, Farmington, CT, United States of America; 3 New York University Wagner School of Public Policy, New York, NY, United States of America; 4 Department of Population Health Sciences, Weill Cornell Medicine, New York, NY, United States of America; 5 New York State Office of Addiction Services and Supports (OASAS), New York, NY, United States of America; PLOS: Public Library of Science, UNITED KINGDOM

## Abstract

**Introduction:**

Regulatory changes made during the COVID-19 public health emergency (PHE) that relaxed criteria for take-home dosing (THD) of methadone offer an opportunity to improve quality of care with a lifesaving treatment. There is a pressing need for research to study the long-term effects of the new PHE THD rules and to test data-driven interventions to promote more effective adoption by opioid treatment programs (OTPs). We propose a two-phase project to develop and test a multidimensional intervention for OTPs that leverages information from large State administrative data.

**Methods and analysis:**

We propose a two-phased project to develop then test a multidimensional OTP intervention to address clinical decision making, regulatory confusion, legal liability concerns, capacity for clinical practice change, and financial barriers to THD. The intervention will include OTP THD specific dashboards drawn from multiple State databases. The approach will be informed by the Health Equity Implementation Framework (HEIF). In phase 1, we will employ an explanatory sequential mixed methods design to combine analysis of large state administrative databases—Medicaid, treatment registry, THD reporting—with qualitative interviews to develop and refine the intervention. In phase 2, we will conduct a stepped-wedge trial over three years with 36 OTPs randomized to 6 cohorts of a six-month clinic-level intervention. The trial will test intervention effects on OTP-level implementation outcomes and patient outcomes (1) THD use; 2) retention in care; and 3) adverse healthcare events). We will specifically examine intervention effects for Black and Latinx clients. A concurrent triangulation mixed methods design will be used: quantitative and qualitative data collection will occur concurrently and results will be integrated after analysis of each. We will employ generalized linear mixed models (GLMMs) in the analysis of stepped-wedge trials. The primary outcome will be weekly or greater THD. The semi-structured interviews will be transcribed and analyzed with Dedoose to identify key facilitators, barriers, and experiences according to HEIF constructs using directed content analysis.

**Discussion:**

This multi-phase, embedded mixed methods project addresses a critical need to support long-term practice changes in methadone treatment for opioid use disorder following systemic changes emerging from the PHE—particularly for Black and Latinx individuals with opioid use disorder. By combining findings from analyses of large administrative data with lessons gleaned from qualitative interviews of OTPs that were flexible with THD and those that were not, we will build and test the intervention to coach clinics to increase flexibility with THD. The findings will inform policy at the local and national level.

## Introduction

Methadone is a highly effective treatment for opioid use disorder (OUD) that has been available since the 1960s [1–7]. In the United States, methadone is provided through clinics called opioid treatment programs (OTPs) that are highly regulated by the Drug Enforcement Agency (DEA), the Substance Abuse and Mental Health Services Administration (SAMHSA), and state and local governments. There are over 1,800 OTPs in the United States [8]; however, the regulations and the OTP system that have emerged since the Vietnam War era reflect stigmatizing and discriminatory notions of people with OUD (PWOUD) that influence clinical practices to this date [9–15].

Prior to the COVID-19 PHE, SAMHSA regulations required the majority of OTP clients to visit clinics almost daily to monitor their methadone dosing [16, 17]. Clients could eventually earn privileges for THD that would allow for self-administration outside the clinic setting, but the bar was high. A client needed to be in treatment for 9 months before being eligible for weekly THD and 12 months before being eligible for bimonthly THD. In addition to the time in treatment criteria, clients need to be abstinent from other substances, including alcohol and cannabis, be ‘compliant’ with treatment, and be relatively high functioning in other life domains [16, 18–22]. The SAMHSA 8-point criteria for compliance and ‘stability’ were not defined in detail and left open to OTP staff clinical discretion. Staff generally defaulted to risk-averse interpretations that were often not aligned with existing data [23]. As a result, clients were subjected to burdensome and demeaning treatment that created barriers to employment or improvements in other domains of life functioning [24–27]. The barriers are particularly troublesome for PWOUD who live in rural areas or with long travel distances to OTPs [28–31]. Notably, Black/African American clients were subject to more restrictive interpretations of the criteria than non-Latinx White clients [32]. The presumed rationale for the restrictive regulations was to mitigate risk of diversion or overdose from methadone [22]; however, the effect has been to limit access and place a heavy burden on clients that has been referred to as “liquid handcuffs [27]”.

In response to the COVID-19 Public Health Emergency (PHE), SAMHSA gave states a temporary option to request a waiver to allow greater flexibility of take-home dosing (THD) of methadone for clients [33]. Thus, the PHE federal rules [33] shifted from time-in-treatment requirements to clinical judgement regarding patient stability. SAMHSA issued an extension of the COVID-19 THD waiver option for states indefinitely [33]. Uptake by states of the new waiver option has been uneven. Not all states applied for the waiver [34, 35], which reflected the variability in local regulatory approaches [36]. SAMHSA recently published a notice of proposed rulemaking to make the flexibility of THD permanent [37]. The response by OTP organizational leadership and staff has been mixed. While some have highlighted the benefits to clients of greater flexibility in THD, others have expressed concern about the potential harm to clients from unsupervised dosing that may result in overdoses, diversion, or program legal liability from potential misuse [21, 38–42].

There is no evidence that greater THD is associated with more overdoses from methadone [43–48]. A recent large and robust study from Ontario, Canada, found that more flexible THD increased retention in care yet was not associated with increased mortality [49]. Cross-national comparisons with other countries that have less restrictive regulations do not demonstrate higher mortality or diversion than in the United States [50–56]. DEA reports show little evidence of large-scale diversion of methadone dispensed through OTPs, and recent studies show that mortality and diversion have been more strongly associated with methadone prescribed for pain management than for OUD [57–59]. Publications based on recent experience from the COVID-19 PHE by two large OTP providers in New York City have reported positive responses from clients, general support from OTP staff, and no evidence of significant increases in overdose or diversion [60, 61].

In New York, as elsewhere in the United States, the adoption of THD by OTPs across the state has been uneven. There is a pressing need for research to study the long-term implications of the PHE THD rules as well as to test data-driven interventions to promote more effective decision making and clinical practice regarding THD by OTPs [22, 62]. We are conducting a two-phase project to develop and test a multidimensional intervention for OTPs that leverages information from large State administrative databases. By combining findings from analyses of large administrative data with lessons gleaned from qualitative interviews of clinics that have implemented THD, we will build scientifically supported knowledge to inform policy at a larger system level. Our protocol is informed by the Health Equity Implementation Framework (HEIF) to guide the development and study of an intervention to increase THD among OTPs. HEIF integrates two research frameworks for conducting implementation studies of interventions targeting underserved populations: 1) the Kilbourne framework for health disparities research; and 2) the Integrated Promoting Action on Research Implementation in Health Services (i-PARIHS) framework. The Kilbourne framework focuses on historical, cultural, and contextual factors that affect client-provider encounters [63]. i-PARIHS posits that optimal implementation occurs when practice facilitation promotes the acceptance and use of a new practice innovation by tailoring it to the recipient’s specific needs [64–66]. Facilitators are the active ingredient that help navigate individuals and teams through complex change processes by addressing: a) the innovation’s degree of fit within the existing practice; b) the motivations, beliefs, goals, characteristics, and resources of the intervention recipients; and c) the inner and outer context in terms of leadership support, culture, past innovation experiences, the learning environment, organizational priorities, capacity for change, regulatory/policy drivers, incentives/mandates, and system stability/instability.

The COMET: Clinics Optimizing MEthadone Take-homes for opioid use disorder study is a two-phase project to develop then test a multidimensional OTP intervention to address clinical decision making, regulatory confusion, legal liability concerns, capacity for clinical practice change, and financial barriers to THD.

We have six main aims:

### Phase 1, Year 1: Intervention development

1. Analyze administrative data to identify factors associated with OTP variation in THD practices and categorize clinics by THD flexibility.
2. Conduct qualitative interviews with leadership and staff of 10 OTPs (5 high and 5 low THD flexibility) on clinical and organizational factors affecting take-home dosing decisions.
3. Complete development of the multidimensional OTP intervention.

### Phase 2, Years 2–5: Stepped-wedge trial

1. Test the effects of the intervention on THD, retention in care, and adverse events.
2. Assess clinic implementation outcomes and conduct qualitative interviews with OTP leadership, staff, and clients on attitudes, experiences and behaviors related to the intervention.
3. Using mixed methods, explore variation in THD associated with race and ethnicity.

Multiple systemic reviews have concluded that addiction treatment in the United States has large gaps in quality of care and limited capacity for clinical program improvement, and there are no agreed upon quality metrics for the SUD treatment field [67–72]. Notably, the workforce has limited levels of education or professional training to implement process change initiatives to improve clinical outcomes [67, 70, 71, 73]. Because of this limitation, the proposed intervention will draw from our current research [74] based on principles of process improvement and the iPARIHS framework to coach OTPs on changing protocols and workflows to adopt more flexible THD [75–82]. One obstacle to adopting new THD practices is uncertainty by the senior leadership of OTPs regarding the financial viability of any change that deviates from established billing practices [60]. As part of this project, we will use State administrative data to develop a revenue projection tool for OTPs; this decision-support tool will be provided to OTP leadership and allows for revenue projections conditional on target THD practices and optimal billing [83, 84].

### Participant and data confidentiality

Every effort will be made to ensure participant confidentiality. Leadership at participating clinics will not be informed about any information regarding participation in the study. All of the personnel and staff on the study team have been certified in NYU’s Human Research Social/Behavioral Research Course through the CITI program. The study team recognizes that the protection of human subjects relies on policies that secure data with personally identifiable information. Each of the investigators has adopted, and will strictly follow, such policies for all data used in the study. Any time data is reported, it will be aggregated with that of other respondents (e.g., to discuss identified themes or key concepts) and never reported with personally identifiable information. No information will be reported that could make it possible for anyone to identify participants in any presentations or written reports about this study. Any identifiable personal information that participants may reveal about themselves during the interviews will be removed from the transcripts. If a direct quote from an interview is utilized in a report or publication (e.g., to exemplify a concept that arose in multiple interviews), it will only be cited using a pseudonym or participant ID number. All information will be stored in our secure, encrypted NYU drives. Personal identifying information will never be shared with anyone outside of the research team. In the event that a subject revokes authorization to collect or use PHI, the investigator, by regulation, retains the ability to use all information collected prior to the revocation of subject authorization. For subjects that have revoked authorization to collect or use PHI, attempts should be made to obtain permission to collect at least vital status (i.e. that the subject is alive) at the end of their scheduled study period.

### Ethics approval

**Fig 1:**
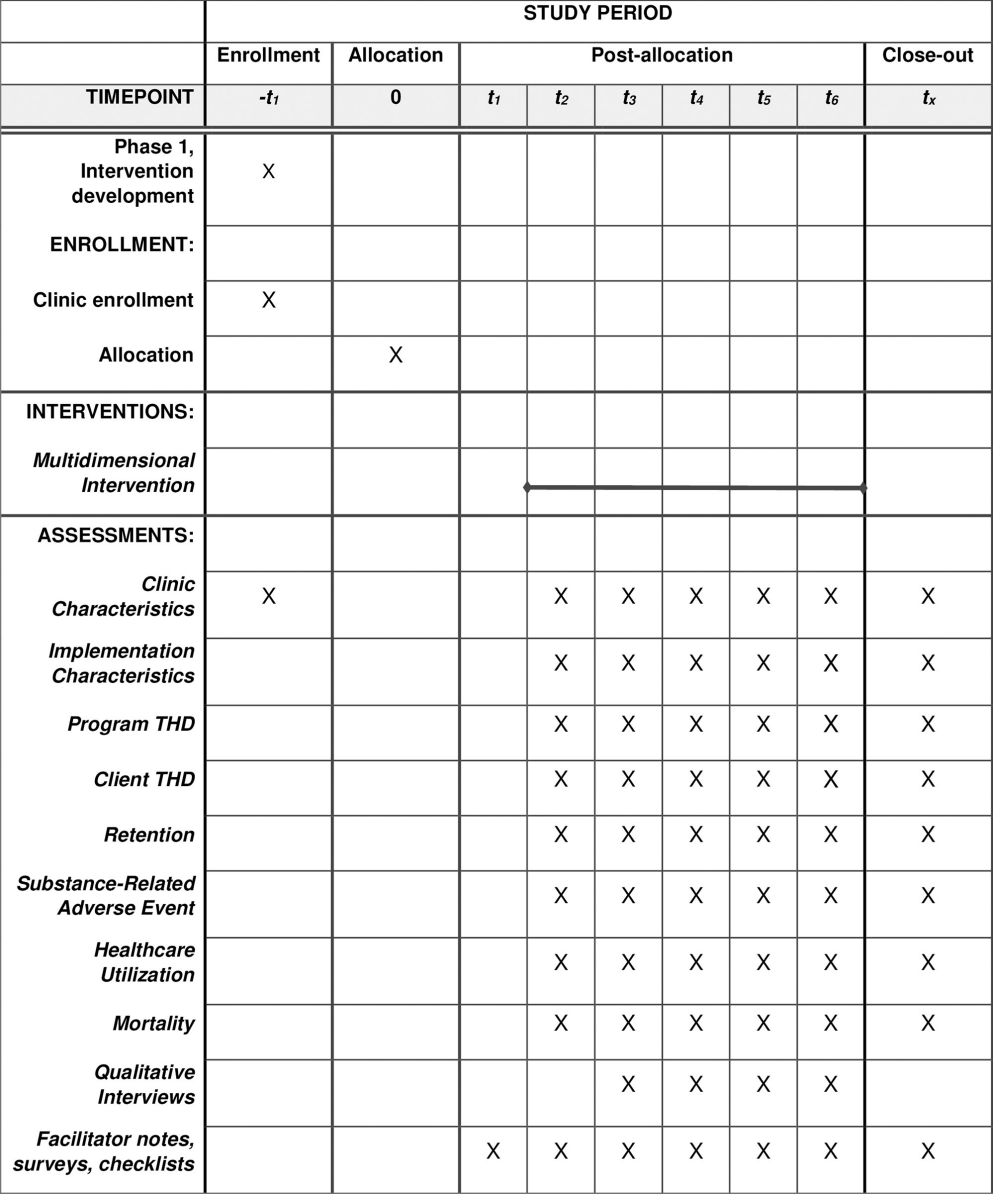
SPIRIT flow diagram for the COMET study.

Ethics approvals have been obtained using a single IRB from NYU Langone Health (#S22-00892), overseeing all sites participating in the study: New York State Office of Addiction Services and Supports (OASAS), University of Connecticut, and Weill Cornell Medicine. The study was registered under Clinicaltrials.gov (NCT05675735), and the SPIRIT checklist was used as a guide for reporting this study protocol (Fig 1).

## Material and methods

### Phase 1. Intervention development

#### Aim 1. Analyze administrative data to identify factors associated with OTP variation in THD practices and categorize clinics by THD flexibility

We will join multiple administrative data sources for years March 2020 to February 2021 to form our analytical dataset. An OASAS treatment registry (Client Data System, CDS) will provide extensive clinical data on clients at treatment admission. All licensed SUD treatment programs in NYS must enter admission and discharge data into the CDS. Medicaid will provide medical diagnoses, healthcare services, OTP visits, and methadone administration billing data. The OTP Opioid Treatment Annual Update Report (OTAU) database will provide weekly, bimonthly, monthly THD measures at clinics. The outcome measure is OTP-level THD use. We will descriptively assess variations in THD flexibility (1. no THD; 2. weekly supply; 3. bimonthly; 4. month supply) and rank clinics.

#### Aim 2. Conduct qualitative interviews with leadership and staff of 10 OTPs (5 high and 5 low THD flexibility) on clinical and organizational factors affecting take-home dosing decisions

We will select 10 clinics for qualitative interviews with staff members to explore clinic-level factors associated with THD practices and technical assistance needed to implement more flexible THD. Clinic staff inclusion will include anyone who works at the 10 clinics that the OASAS client data system generates from the quantitative analysis in year 1. The semi-structured 1:1 interviews will be recorded and transcribed and then using directed content analysis following steps recommended for content analysis [85–87]. We will use rapid analysis using matrices to analyze the data in real-time [88–90]. Researchers will make field notes immediately after interviews. Weekly meetings will be held to review coding decisions, discuss discrepancies, and check progress. As emerging concepts are identified, we will adapt the existing coding structure. When the analysis is complete, the team will meet to review summaries of the qualitative results and refine hypotheses about the contextual factors and strategies that lead to better outcomes and those that might be barriers to success.

#### Aim 3. Complete development of the multidimensional OTP intervention

We plan to convene an online stakeholder group meeting comprised of leadership from providers, health insurers, clients, and family members to provide input on qualitative research findings and subsequent intervention design.

### Intervention structure

Table 1 provides an overview of the multidimensional OTP intervention and its goals. The intervention draws from a similar approach our team is currently employing to coach non-OTP SUD treatment clinics on quality improvement [74]. The proximal goal will be to increase OTP uptake of flexible THD, which in turn should result in increased retention in care without increasing adverse healthcare events or mortality. The intervention addresses facilitators and barriers to THD practices as identified by existing studies as well as those identified during phase 1 of the current project. Guided by HEIF and research on organization change [91–95], the intervention will be designed to address the information gaps, training needs, and beliefs of individuals across the organizations. Senior leadership have purview over financial and legal matters. Medical directors and nursing staff are responsible for dosing decisions consistent with best clinical practices. Counseling and administrative staff are involved in multiple dimensions of clinic operations. Table 1 describes the components of the intervention as well as the audience within the OTP for each. It aligns with the HEIF by addressing clinical encounter factors (e.g., THD best practices), inner and outer context (e.g., regulatory guidance, data feedback), and economic influences (e.g., revenue projection tool). A designated external facilitator will work closely with each clinic using remote conferencing capabilities, as in our current trial. These facilitators will be trained in quality improvement practices and data management as well as relevant clinical topics (e.g., THD best practices). They will be supervised in weekly intervention oversight meetings by study team members. Facilitators will work closely with an identified member of clinic leadership, a clinical champion, and a small implementation team (e.g., 4–6 staff).

**Table 1:** COMET intervention.

Component	Who	Description
Data Dashboard	All	Performance feedback is an organizational intervention with robust scientific support [91–93]. Drawing from analytical methods applied during phase 1 of the project, provide clinic-level reports that show relative performance compared to other OTPs within the State on THD, retention, and adverse healthcare events. Relative performance will be adjusted for clients’ demographic and clinical characteristics [96–99]. The data feedback will specifically detail THD performance by race, ethnicity, and gender. Reports would be available for all staff.
Legal/Regulatory	All	To address barriers related to uncertainty over legal and regulatory risk [21, 38–42], provide guidance on Federal and State requirements for THD as well as guidance on managing legal liability risk. Guidance would be available for all staff.
Financial Guidance	SL	To address senior leadership concerns about financial viability, provide a revenue projection tool along with training to allow clinics to model out the financial effects of using new THD bundled payment billing. The tool and guidance will walk through number of clients with different THD schedules and projections for revenue based on optimal billing. Leadership would be able to conduct ‘what-if’ analyses under varying assumptions of THD practices [83, 84].
THD Best Practices	CL	Drawing from extant research as well as from findings from qualitative studies conducted during phase 1, provide training on clinical best practices for THD. The training would address parameters for determining level of client stability, protocols for monitoring client status, and use of technology [100–102]. The training will also highlight any clinic THD variation by race, ethnicity, or gender. The training would specifically address concerns and best practices for managing risks of methadone overdose and/or diversion. Our team will provide training to Medical Directors and nursing staff.
Process Change	C+A	Training and support on process improvement strategies drawn from organizational change management. The OTP will be asked to designate a clinic implementation team led by local champion to review data and guide practice change projects [67–72]. The project facilitator will train and support the implementation team on rapid cycle change strategies drawn from management science and used in our current clinic-level trial.

Table Notes: All = All OTP staff, including senior leadership, medical, counseling, and administrators; SL = senior leadership, including chief executive officer, financial, and clinic directors; CL = Clinical staff, including medical (physicians, nurses, nurse practitioners, physician assistants) and counselors; C+A = Clinical and administrative staff (e.g., reception, billing)

### Phase 2. Stepped-wedge trial

We propose an embedded mixed methods design, which places the qualitative findings in context of the larger trial. We will assess outcomes quantitatively and answer the primary research questions, then use qualitative data to answer secondary questions. In years 2–5, we will employ a stepped-wedged randomized control trial with 36 OTPs chosen by the OASAS client data system which will be randomized into 6 cohorts of a six-month intervention.

### Recruitment of sites

OTPs will be recruited to participate in this stepped-wedge trial by State partners via email invitations, announcements via State listservs, and announcements at relevant meetings. A total of 36 programs will be recruited at the beginning of the trial (Year 2). OTP that participated in the qualitative interviews during Phase 1 can also participate in the stepped-wedge trial. We will exclude clinics that already have very flexible THD policies (e.g., greater than 75% of clients are on weekly or greater THD schedule). The intervention is delivered to the leadership and staff members at the clinic-level. Zoom meetings will be conducted with directors of programs to confirm their enrollment and to explain the timeline and expectations. The study team will monitor enrollment and if enough clinics do not enroll, additional recruitment strategies will be discussed with the study team and implemented. In order to retain clinics in the trial, the study team will stay in regular contact with the clinic program directors. If a clinic is considering withdrawing from the study, the study team will schedule a meeting to further discuss and try to address reasons for desired withdrawal. We expect that offering the intervention to clinics will be attractive because the intervention will offer training and external facilitation free of charge. The study team has found in their experience that providing Statewide technical assistance is very welcome during the period of state reforms and the COVID-19 PHE; therefore, we do not anticipate problems with enrollment.

#### Aim 4. Test the effects of the intervention on THD, retention in care, and adverse events

*Data*. Similar to Phase 1 of the study, we will join multiple administrative data sources for years 2020 to 2027 to form our analytical dataset. We will use CDS, Medicaid claims, OTAU, and NYS vital statistics data to form the analytic dataset. The New York State vital statistics reporting will provide data on mortality for OTP clients. Our experience has been that accurate overdose data is reported with a long lag time due to capacity constraints for extensive forensic analyses across many of the Local Government Units (LGUs). Challenges in obtaining timely overdose data are not unique to New York and are a function of the complexity involved in assigning cause of death as well as local resources available to conduct these assessments [45, 103]. Consequently, we rely on obtaining all-cause mortality for clients in the OTPs participating in the trial. Other OASAS data will provide basic operational and staffing data for each of the OTPs. We have extensive experience joining these databases. Specifically, we have joined 83% of all OTP clients found in the OASAS registry to Medicaid claims data.

*Measures*. The outcome measures include: client-level THD use, retention in treatment, substance-related adverse events, all-cause adverse events, and all-cause mortality.

Client THD use will be estimated using billing data from Medicaid, identify clients receiving weekly, bimonthly, and monthly THD. Retention will be derived from the CDS and be the number of days until discharge. Substance-related adverse events are defined as the following: overdose, medically managed or supervised withdrawal services, hospitalizations or emergency department visits with a primary diagnosis of substance use disorder. All-cause adverse events will be estimated by examining any emergency department visits or hospitalizations irrespective of diagnosis. All-cause mortality will be defined as OTP client deaths irrespective of cause. As mentioned previously, we focus on all-cause mortality due to challenges with reliable data reporting on methadone and other opioid related overdose deaths [45, 103]. All-cause mortality will provide a broad indicator of health associated with longer retention in care as well as subsume any overdoses that may emerge from greater THD. A complete and detailed list of measures that will be used in the study can be found in Table 2.

**Table 2:** COMET study measures.

Domain	Variables	Source
***Individual Baseline Characteristics***		
Demographic	Client age; race/ethnicity; gender; education; unhoused status; employment; criminal justice involvement	OASAS Medicaid
Clinical complexity	Mental health conditions (e.g., PTSD, ADHD); serious mental illness (SMI) (e.g., schizophrenia); chronic physical conditions (e.g., HIV, HCV)	Medicaid
Substance Use Patterns	Age of onset; heroin use; other substances used (alcohol, stimulants, cannabis, cocaine, and etc); frequency of use; and injection drug use (IDU)	OASAS
Prior Healthcare Utilization	History of use of outpatient, inpatient, emergency department	Medicaid
***Individual-level Treatment Process***		
Psychosocial Interventions*	Behavioral treatment (i.e., group, individual, peer, or family counseling); telepractice; urine toxicology testing; mental health treatment; Medicaid reimbursed transportation	Medicaid
***OTP Organizational Characteristics***		
Structure	Program size (i.e., annual patient volume); hospital system affiliation; number of OTPs within parent organization	OASAS Medicaid
Staffing	Number of staff; staff professionalization (e.g., degrees); gender and race/ethnicity; client-to-staff ratio	OASAS
Clinical Process	average monthly volume claims for individual, group, peer, and family counseling; average monthly volume claims for telepractice use; monthly volume for urine toxicology testing	Medicaid
Program THD*	OTP % of clients receiving weekly, bimonthly, and monthly THD	OASAS
***Outcome Variables***		
Client THD*	Client THD in ordinal measure of weekly, bimonthly, and monthly dose	Medicaid
Retention*	Time until treatment exit	OASAS
Substance-related Adverse Event*	SUD-related emergency department visit or hospitalization, including for overdose; medically managed or supervised withdrawal services	Medicaid
Healthcare Utilization*	All-cause emergency department visits and hospitalizations	Medicaid
Mortality	Client level all-cause mortality	DOH

Table Notes: OASAS = CDS, OTP THD, and program databases; DOH = New York Department of Health Vital Statistics * Time-varying variables

### Statistical power

To estimate power for select outcomes, we used a combination of PASS 2022 and Monte Carlo simulation [104]. Using administrative data to estimate sample sizes and baseline rates (μ), we compute detectable differences for 80% power, α = 0.05, ICC = 0.05, and SW-RCT with 6 crossover points, and 6 clinics randomized to start at each crossover point. For weekly or greater THD (i.e., picking up doses weekly or less often), we assume the current prevalence is 0.45. With 36 clinics and approximately 350 clients observed in each clinic in each of the seven periods (every six-month) (n≈88,200 total observations), power is 93% to detect a small increase (OR = 1.1) in the odds of weekly or greater THD. This corresponds to an increase from a prevalence of 0.45 under the control condition to 0.47 after intervention. Even when considering racial/ethnic patient subgroups making up just 10% of clinic clients (n≈35 per site and period), power is 92% to detect a modest increase in the odds of weekly or greater THD (OR = 1.3; an increase from 0.45 to 0.51). Because power is sufficient to detect small intervention effects for the binary outcomes of weekly or greater THD and adverse events, it is also sufficient to detect small intervention effects on retention in care.

### Analysis of the stepped-wedge trial

**Fig 2:**
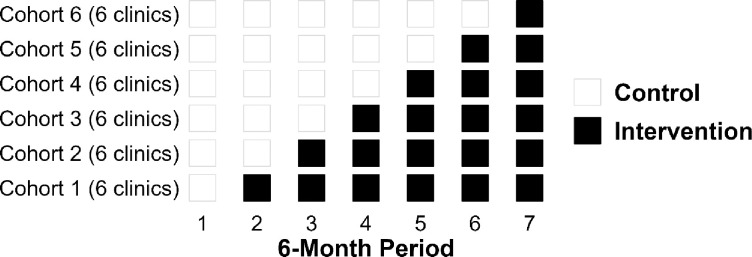
COMET implementation: Stepped-wedge design.

Fig 2 depicts the stepped-wedge research design. Clinics are randomized to onset of intervention in one of six time periods over the three years of the intervention rollout (years 2–4 of the project) and tracked using administrative data. Data for all OTP clients during each of the seven six-month periods (n≈350/clinic) will be drawn from administrative data. The first six-month period will be a baseline prior to any intervention. Six clinics will be assigned randomly to one of six time periods for onset of intervention.

We will employ GLMM models commonly used in the analysis of stepped-wedge trials [105, 106]. The models will take the form:

$$F(\mu_{ijk})=\mu +\beta_{j}+{\delta X}_{ij}+\alpha_{i}+\phi_{ik},\alpha_{i}∼N(0,\tau_{\alpha }^{2}),\gamma_{ij}∼N(0,\tau_{\phi }^{2})$$

where *β_j_* is a fixed effect for time, *X_ij_* is an indicator for intervention in clinic *i* at time *j* (coded 0 for the control condition, then 1 from when intervention begins in the clinic through end of study), and *δ* is the treatment effect. The models have random effects for clustering at the clinic and individual patient levels, which are assumed to be normally distributed with mean zero and variances $\tau_{\alpha }^{2}$ and $\tau_{\phi }^{2}$, respectively. We will estimate the effect of intervention while controlling for secular trends and adjusting for clustering within clinic (*α_i_*) and individual (*ϕ_ik_*), using mixed effects modeling with binary distribution and logit link. Models will be fit using the *glmmTMB* package using R software [107, 108]. The primary outcome will be weekly or greater THD, and the fixed-effects coefficients for the intervention effect, exponentiated, will indicate how the intervention increases the odds of weekly or greater THD. Similar mixed models will consider other THD frequencies as outcomes (i.e., bimonthly and monthly). The same mixed logistic regression model will be used with any adverse events as the outcome, but a one-sided test of non-inferiority (i.e., adverse events are no worse under intervention than under the control condition) will be used [109]. For the retention outcome, a multilevel discrete-time survival model will be used [110–112]. An expanded person-period dataset will be constructed [113]. The outcome will have two possible states in any time period: 0 = retained in treatment; 1 = treatment exit. The multilevel analysis approach can accommodate recurring events for the same individual, with individuals leaving the set of patients “at risk” for dropout until treatment is initiated again. Analysis of the person-period dataset will use a mixed-effects regression model with a complementary log-log link function and random effects for patient and clinic. With the complementary log-log link, the coefficients can be interpreted as the relative effect on the hazard of event occurrence. In a sensitivity analysis, we will restrict the retention outcome to patients who have been in treatment for less than 12 months at the start of the study. The discrete-time multilevel model accommodates both time-invariant patient characteristics as well as time-varying patient and clinic characteristics as explanatory variables. Our focus will be on the time-varying intervention condition variable at the clinic-level, adjusting for patient characteristics such as demographics and prior treatment experience. Intervention effects will be visualized by plotting fitted hazard of dropout against month since treatment initiation separately for control and intervention observations. The multilevel discrete-time survival model can be used for other time to event outcomes such as time to all-cause mortality.

#### Aim 5. Assess clinic implementation outcomes and conduct qualitative interviews with OTP leadership, staff, and clients on attitudes, experiences and behaviors related to the intervention

We will examine OTP staff and leadership attitudes, experiences, and behaviors related to implementing the OTP intervention. We plan to collect diverse data: 1) implementation surveys, 2) semi-structured interviews, and 3) external facilitator notes, surveys, and checklists [114–116]. An online version of the Klein implementation survey [117, 118] will be administered to staff at 6-months post-intervention. Semi-structured interviews (n = 72) will be conducted with at least one clinical and one member of OTP executive leadership at each OTP at the end of the six-month intervention phase. An interview guide will be created to reflect the dimensions of the HEIF, including patient-provider encounter factors from the Kilbourne framework as well as implementation factors from the i-PARIHS model [63, 94, 95, 119]. In order to inform replication and dissemination of this intervention model, as well as contextual factors affecting sites’ ability to implement THD, the facilitator will complete logs after each interaction with a study site [114]. Client interviews (n = 40) from participating OTPs will explore experiences related to THD, notably among Black/African American and Latinx clients. We will include patients who are: 1) aged 18 or older; and 2) receiving take-home methadone for at least 30 days. An interview guide will be created to reflect the dimensions of the HEIF. We will use maximum variation purposeful sampling by recruiting clients from participating OTPs in urban and rural locations as well as areas with variation in population characteristics. Further, we will recruit clients who are on a variety of THD schedules and ensure that we have representation based on gender, race, and ethnicity. Clients will receive a $30 incentive to participate [120].

*Analysis*. For the survey results, staff responses will be aggregated and group means and standard deviations will be calculated. Multilevel modeling will be used to examine change over time in survey scores given the nested structure of the data (staff nested within clinics) [121]. The semi-structured interviews will be recorded and transcribed and then using directed content analysis following steps recommended for content analysis [85–87]. We will use Dedoose to manage, code, organize, and examine patterns in the data. Researchers will make field notes immediately after interviews. We will use a deductive approach such that a codebook with codes and operational definitions will be created prior to analysis using key elements from HEIF. We will use a continuous process of coding, categorizing, and reviewing the raw data to reflect on the analysis at various points and make revisions as needed (e.g., recoding data). Weekly meetings will be held to review coding decisions, discuss discrepancies, and check progress. We will use the first few transcripts to refine the coding scheme, and additional transcripts will be compared to previously coded transcripts to ensure the consistent assignment of codes. As emerging concepts are identified, we will adapt the existing coding structure. After the team has reviewed the coding structure and all initial transcripts have been reviewed in depth by the team, trained project staff will independently code all transcripts using the final coding scheme. Twenty percent of the transcripts will be double coded to assess inter-coder agreement. Any differences in coding will be discussed and resolved after discussion with the investigators. We also will create an analysis audit trail to document all analytic decisions.

#### Aim 6. Using mixed methods, explore variation in THD associated with race and ethnicity

Throughout quantitative analysis, we will examine by including additional terms in the mixed-effects logistic regression models and in the multilevel discrete-time survival model to incorporate interactions between the intervention effect and patient race/ethnicity. If significant interaction effects are detected, simple main effects of intervention will be estimated for each racial/ethnic group to understand how the intervention effect may vary and to identify any inequities in intervention impact. Throughout qualitative analysis, we will explore variation in THD experiences among Black/African American and Latinx clients and those clinics that serve more Black/African American and Latinx populations.

### Dissemination

This study will comply with the NIH Public Access Policy, which ensures that the public has access to the published results of NIH funded research. It requires scientists to submit final peer-reviewed journal manuscripts that arise from NIH funds to the digital archive PubMed Central upon acceptance for publication. This trial has been registered with ClinicalTrials.gov database (#NCT05675735). The data generated in this study will be presented at national or international conferences and published in a timely fashion. All final peer-reviewed manuscripts that arise from this proposal will be submitted to the digital archive PubMed Central. Project data will be made available to the Data Ecosystem in a manner that is consistent with Data Use Agreements with the data owners.

### Status and timeline

The recruitment of initial interviews to inform the design of the intervention has begun. The working groups are meeting regularly to design an intervention by the end of Year 1. Site recruitment will start by August 2023. All participating HCPs will complete training by Year 2. Data collection will be completed by August 2026. Data analysis and reporting of results will be completed by August 2027.

## Conclusion

Currently, there is a national debate about balancing safety concerns over more flexible THD against the benefits of client retention and quality of life. Studies have not found increased overdoses or diversion under more flexible THD rules during the PHE; however, longer term studies are needed to understand best THD practices and outcomes. This study leverages large administrative datasets that can inform immediate policy questions of supporting flexible THD. The intervention targets improvement in treatment for OUD and tracks outcomes for healthcare, overdose, and mortality. It will develop and test actionable organizational interventions to promote better person-centered treatment.

## Supporting information

S1 Checklist SPIRIT 2013 checklist: Recommended items to address in a clinical trial protocol and related documents*. (DOCX)

S1 File (DOCX)

## References

[1] Mattick RP, Breen C, Kimber J, Davoli M. Methadone maintenance therapy versus no opioid replacement therapy for opioid dependence. Cochrane Database Syst Rev. 2009;(3):CD002209. Epub 2009/07/10. doi: 10.1002/14651858.CD002209.pub2 . 19588333 PMC7097731

[2] Kleber HD. Methadone Maintenance 4 Decades Later. JAMA. 2008;300(19):2303. doi: 10.1001/jama.2008.648 19017918

[3] Dole VP. A Medical Treatment for Diacetylmorphine (Heroin) Addiction. JAMA. 1965;193(8):646. doi: 10.1001/jama.1965.03090080008002 14321530

[4] Dole VP. Methadone Maintenance Treatment for 25,000 Heroin Addicts. JAMA: The Journal of the American Medical Association. 1971;215(7):1131. doi: 10.1001/jama.1971.03180200055012 5107884

[5] Mattick RP, Breen C, Kimber J, Davoli M. Buprenorphine maintenance versus placebo or methadone maintenance for opioid dependence. Cochrane Database Syst Rev. 2014;(2):CD002207. Epub 2014/02/07. doi: 10.1002/14651858.CD002207.pub4 . 24500948 PMC10617756

[6] Gryczynski J, Mitchell SG, Jaffe JH, Kelly SM, Myers CP, O’Grady KE, et al. Retention in methadone and buprenorphine treatment among African Americans. J Subst Abuse Treat. 2013;45(3):287–92. Epub 2013/04/10. doi: 10.1016/j.jsat.2013.02.008 ; PubMed Central PMCID: PMC3714350. 23566446 PMC3714350

[7] Medications for Opioid Use Disorder Save Lives. Leshner AIM, Michelle, editor. Washington, DC: National Academies of Science, Engineering, and Medicine; 2019. 30896911

[8] National Association of State Alcohol and Drug Abuse Directors (NASADAD). Technical brief: Census of opioid treatment programs. 2022.

[9] Earnshaw V, Smith L, Copenhaver M. Drug Addiction Stigma in the Context of Methadone Maintenance Therapy: An Investigation into Understudied Sources of Stigma. Int J Ment Health Addict. 2013;11(1):110–22. Epub 2013/08/21. doi: 10.1007/s11469-012-9402-5 ; PubMed Central PMCID: PMC3743126. 23956702 PMC3743126

[10] Jackson DS, Nguemeni Tiako MJ, Jordan A. Disparities in Addiction Treatment: Learning from the Past to Forge an Equitable Future. Med Clin North Am. 2022;106(1):29–41. Epub 2021/11/27. doi: 10.1016/j.mcna.2021.08.008 . 34823733

[11] Peterkin A, Davis CS, Weinstein Z. Permanent Methadone Treatment Reform Needed to Combat the Opioid Crisis and Structural Racism. J Addict Med. 2021. Epub 20210319. doi: 10.1097/adm.0000000000000841 . 33758114

[12] Hansen HB, Siegel CE, Case BG, Bertollo DN, Dirocco D, Galanter M. Variation in Use of Buprenorphine and Methadone Treatment by Racial, Ethnic, and Income Characteristics of Residential Social Areas in New York City. The Journal of Behavioral Health Services & Research. 2013;40(3):367–77. doi: 10.1007/s11414-013-9341-3 23702611 PMC3818282

[13] Hansen H, Siegel C, Wanderling J, Dirocco D. Buprenorphine and methadone treatment for opioid dependence by income, ethnicity and race of neighborhoods in New York City. Drug and Alcohol Dependence. 2016;164:14–21. doi: 10.1016/j.drugalcdep.2016.03.028 27179822 PMC5539992

[14] Davis CS, Carr DH. Legal and policy changes urgently needed to increase access to opioid agonist therapy in the United States. Int J Drug Policy. 2019;73:42–8. Epub 20190720. doi: 10.1016/j.drugpo.2019.07.006 . 31336293

[15] Allen B, Nolan ML, Paone D. Underutilization of medications to treat opioid use disorder: What role does stigma play? Subst Abus. 2019;40(4):459–65. Epub 20190924. doi: 10.1080/08897077.2019.1640833 . 31550201

[16] Davis CS, Samuels EA. Opioid Policy Changes During the COVID-19 Pandemic—and Beyond. J Addict Med. 2020;14(4):e4–e5. doi: 10.1097/ADM.0000000000000679 ; PubMed Central PMCID: PMC7273953. 32433363 PMC7273953

[17] Eaves E, Trotter R 2nd, Baldwin J. Another silver lining?: Anthropological perspectives on the promise and practice of relaxed restrictions for telemedicine and medication-assisted treatment in the context of COVID-19. Hum Organ. 2020;79(4):292–303. Epub 20201202. doi: 10.17730/1938-3525-79.4.292 ; PubMed Central PMCID: PMC7861509. 33551465 PMC7861509

[18] Administration SAaMHS. Statutes, Regulations, and Guidelines 2022 [updated 2022/02/01/]. Available from: https://www.samhsa.gov/medication-assisted-treatment/statutes-regulations-guidelines.

[19] Center for Substance Abuse T. SAMHSA/CSAT Treatment Improvement Protocols. Medication-Assisted Treatment for Opioid Addiction in Opioid Treatment Programs. Rockville (MD): Substance Abuse and Mental Health Services Administration (US); 2005. 22514849

[20] 42 CFR § 8.12—Federal opioid treatment standards Legal Information Institute: Cornell Law School; [cited 2022 February 5]. Available from: https://www.law.cornell.edu/cfr/text/42/8.12.

[21] Madden EF, Christian BT, Lagisetty PA, Ray BR, Sulzer SH. Treatment provider perceptions of take-home methadone regulation before and during COVID-19. Drug Alcohol Depend. 2021;228:109100. Epub 20210923. doi: 10.1016/j.drugalcdep.2021.109100 ; PubMed Central PMCID: PMC8459541. 34600251 PMC8459541

[22] Joudrey PJ, Bart G, Brooner RK, Brown L, Dickson-Gomez J, Gordon A, et al. Research priorities for expanding access to methadone treatment for opioid use disorder in the United States: A National Institute on Drug Abuse Center for Clinical Trials Network Task Force report. Subst Abus. 2021;42(3):245–54. Epub 2021/10/05. doi: 10.1080/08897077.2021.1975344 ; PubMed Central PMCID: PMC8790761. 34606426 PMC8790761

[23] Larance B, Carragher N, Mattick RP, Lintzeris N, Ali R, Degenhardt L. A latent class analysis of self-reported clinical indicators of psychosocial stability and adherence among opioid substitution therapy patients: do stable patients receive more unsupervised doses? Drug Alcohol Depend. 2014;142:46–55. Epub 2014/07/13. doi: 10.1016/j.drugalcdep.2014.05.018 . 25015687

[24] Anstice S, Strike CJ, Brands B. Supervised methadone consumption: client issues and stigma. Subst Use Misuse. 2009;44(6):794–808. Epub 2009/05/16. doi: 10.1080/10826080802483936 . 19444722

[25] Deering DEA, Sheridan J, Sellman JD, Adamson SJ, Pooley S, Robertson R, et al. Consumer and treatment provider perspectives on reducing barriers to opioid substitution treatment and improving treatment attractiveness. Addict Behav. 2011;36(6):636–42. Epub 2011/02/01. doi: 10.1016/j.addbeh.2011.01.004 . 21276664

[26] Treloar C, Fraser S, Valentine K. Valuing methadone takeaway doses: The contribution of service-user perspectives to policy and practice. Drugs: Education, Prevention and Policy. 2007;14(1):61–74. doi: 10.1080/09687630600997527

[27] Frank D, Mateu-Gelabert P, Perlman DC, Walters SM, Curran L, Guarino H. "It’s like ’liquid handcuffs": The effects of take-home dosing policies on Methadone Maintenance Treatment (MMT) patients’ lives. Harm Reduct J. 2021;18(1):88. Epub 2021/08/16. doi: 10.1186/s12954-021-00535-y ; PubMed Central PMCID: PMC8364307. 34391436 PMC8364307

[28] Amiri S, Lutz RB, McDonell MG, Roll JM, Amram O. Spatial access to opioid treatment program and alcohol and cannabis outlets: analysis of missed doses of methadone during the first, second, and third 90 days of treatment. Am J Drug Alcohol Abuse. 2020;46(1):78–87. Epub 2019/06/27. doi: 10.1080/00952990.2019.1620261 . 31237791

[29] Joudrey PJ, Edelman EJ, Wang EA. Drive Times to Opioid Treatment Programs in Urban and Rural Counties in 5 US States. Jama. 2019;322(13):1310–2. Epub 2019/10/02. doi: 10.1001/jama.2019.12562 ; PubMed Central PMCID: PMC6777265. 31573628 PMC6777265

[30] Kiang MV, Barnett ML, Wakeman SE, Humphreys K, Tsai AC. Robustness of estimated access to opioid use disorder treatment providers in rural vs. urban areas of the United States. Drug Alcohol Depend. 2021;228:109081. Epub 20210924. doi: 10.1016/j.drugalcdep.2021.109081 ; PubMed Central PMCID: PMC8595811. 34600256 PMC8595811

[31] Lister JJ, Lister HH. Improving methadone access for rural communities in the USA: lessons learned from COVID-19 adaptations and international models of care. Rural Remote Health. 2021;21(4):6770. Epub 20211110. doi: 10.22605/RRH6770 . 34757760

[32] D’Aunno T. Variations in Methadone Treatment Practices. JAMA. 1992;267(2):253. doi: 10.1001/jama.1992.03480020063032 1727522

[33] Methadone Take-Home Flexibilities Extension Guidance: SAMHSA; 2021 [cited 2022 February 5]. Available from: https://www.samhsa.gov/medication-assisted-treatment/statutes-regulations-guidelines/methadone-guidance.

[34] Andraka-Christou B, Bouskill K, Haffajee RL, Randall-Kosich O, Golan M, Totaram R, et al. Common themes in early state policy responses to substance use disorder treatment during COVID-19. Am J Drug Alcohol Abuse. 2021;47(4):486–96. Epub 20210428. doi: 10.1080/00952990.2021.1903023 ; PubMed Central PMCID: PMC8564552. 33909518 PMC8564552

[35] Nesoff ED, Marziali ME, Martins SS. The estimated impact of state-level support for expanded delivery of substance use disorder treatment during the COVID-19 pandemic. Addiction. 2021. Epub 20211207. doi: 10.1111/add.15778 . 34873783 PMC9081157

[36] Jackson JR, Harle CA, Silverman RD, Simon K, Menachemi N. Characterizing variability in state-level regulations governing opioid treatment programs. J Subst Abuse Treat. 2020;115:108008. Epub 20200424. doi: 10.1016/j.jsat.2020.108008 . 32600617

[37] Notice of proposed rulemaking: Medications for the Treatment of Opioid Use Disorder, (2022).

[38] Treitler PC, Bowden CF, Lloyd J, Enich M, Nyaku AN, Crystal S. Perspectives of opioid use disorder treatment providers during COVID-19: Adapting to flexibilities and sustaining reforms. J Subst Abuse Treat. 2022;132:108514. Epub 20210531. doi: 10.1016/j.jsat.2021.108514 ; PubMed Central PMCID: PMC8630075. 34098210 PMC8630075

[39] Hatch-Maillette MA, Peavy KM, Tsui JI, Banta-Green CJ, Woolworth S, Grekin P. Re-thinking patient stability for methadone in opioid treatment programs during a global pandemic: Provider perspectives. Journal of Substance Abuse Treatment. 2021;124:108223. doi: 10.1016/j.jsat.2020.108223 33342667 PMC8005420

[40] Goldsamt LA, Rosenblum A, Appel P, Paris P, Nazia N. The impact of COVID-19 on opioid treatment programs in the United States. Drug Alcohol Depend. 2021;228:109049. Epub 20210924. doi: 10.1016/j.drugalcdep.2021.109049 ; PubMed Central PMCID: PMC8461004. 34600258 PMC8461004

[41] Hunter SB, Dopp AR, Ober AJ, Uscher-Pines L. Clinician perspectives on methadone service delivery and the use of telemedicine during the COVID-19 pandemic: A qualitative study. J Subst Abuse Treat. 2021;124:108288. Epub 20210113. doi: 10.1016/j.jsat.2021.108288 ; PubMed Central PMCID: PMC7833320. 33771285 PMC7833320

[42] Levander XA, Pytell JD, Stoller KB, Korthuis PT, Chander G. COVID-19-related policy changes for methadone take-home dosing: A multistate survey of opioid treatment program leadership. Subst Abus. 2022;43(1):633–9. Epub 20211019. doi: 10.1080/08897077.2021.1986768 ; PubMed Central PMCID: PMC8810732. 34666636 PMC8810732

[43] Amram O, Amiri S, Panwala V, Lutz R, Joudrey PJ, Socias E. The impact of relaxation of methadone take-home protocols on treatment outcomes in the COVID-19 era. Am J Drug Alcohol Abuse. 2021;47(6):722–9. Epub 20211020. doi: 10.1080/00952990.2021.1979991 . 34670453

[44] Trujols J, Larrabeiti A, Sànchez O, Madrid M, De Andrés S, Duran-Sindreu S. Increased flexibility in methadone take-home scheduling during the COVID-19 pandemic: Should this practice be incorporated into routine clinical care? J Subst Abuse Treat. 2020;119:108154. Epub 20201003. doi: 10.1016/j.jsat.2020.108154 ; PubMed Central PMCID: PMC7532346. 33032860 PMC7532346

[45] Brothers S, Viera A, Heimer R. Changes in methadone program practices and fatal methadone overdose rates in Connecticut during COVID-19. J Subst Abuse Treat. 2021;131:108449. Epub 2021/06/08. doi: 10.1016/j.jsat.2021.108449 . 34098303 PMC9758251

[46] Figgatt MC, Salazar Z, Day E, Vincent L, Dasgupta N. Take-home dosing experiences among persons receiving methadone maintenance treatment during COVID-19. J Subst Abuse Treat. 2021;123:108276. Epub 20210108. doi: 10.1016/j.jsat.2021.108276 ; PubMed Central PMCID: PMC8060693. 33612201 PMC8060693

[47] Hazan J, Congdon L, Sathanandan S, Grewal P. An analysis of initial service transformation in response to the COVID-19 pandemic in two inner-city substance misuse services. Journal of Substance Use. 2021;26(3):275–9. doi: 10.1080/14659891.2020.1820089

[48] Saloner B, Krawczyk N, Solomon K, Allen ST, Morris M, Haney K, et al. Experiences with substance use disorder treatment during the COVID-19 pandemic: Findings from a multistate survey. Int J Drug Policy. 2021;101:103537. Epub 2021/12/07. doi: 10.1016/j.drugpo.2021.103537 ; PubMed Central PMCID: PMC8602971. 34871945 PMC8602971

[49] Gomes T, Campbell TJ, Kitchen SA, Garg R, Bozinoff N, Men S, et al. Association Between Increased Dispensing of Opioid Agonist Therapy Take-Home Doses and Opioid Overdose and Treatment Interruption and Discontinuation. Jama. 2022;327(9):846–55. Epub 2022/03/02. doi: 10.1001/jama.2022.1271 . 35230394 PMC8889466

[50] Nolan S, Hayashi K, Milloy MJ, Kerr T, Dong H, Lima VD, et al. The impact of low-threshold methadone maintenance treatment on mortality in a Canadian setting. Drug Alcohol Depend. 2015;156:57–61. Epub 2015/10/13. doi: 10.1016/j.drugalcdep.2015.08.037 ; PubMed Central PMCID: PMC4633383. 26455554 PMC4633383

[51] Langendam MW, van Brussel GH, Coutinho RA, van Ameijden EJ. The impact of harm-reduction-based methadone treatment on mortality among heroin users. Am J Public Health. 2001;91(5):774–80. Epub 2001/05/10. doi: 10.2105/ajph.91.5.774 ; PubMed Central PMCID: PMC1446673. 11344886 PMC1446673

[52] Fraser S, valentine k, Treloar C, Macmillan K. Methadone maintenance treatment in New South Wales and Victoria: Takeaways, diversion and other key issues. Sydney, Australia: National Centre in HIV Social Research, 2007.

[53] Strike C, Millson M, Hopkins S, Smith C. What is low threshold methadone maintenance treatment? Int J Drug Policy. 2013;24(6):e51–6. Epub 2013/06/08. doi: 10.1016/j.drugpo.2013.05.005 . 23743178

[54] Priest KC, Gorfinkel L, Klimas J, Jones AA, Fairbairn N, McCarty D. Comparing Canadian and United States opioid agonist therapy policies. Int J Drug Policy. 2019;74:257–65. Epub 2019/02/16. doi: 10.1016/j.drugpo.2019.01.020 ; PubMed Central PMCID: PMC6689455. 30765118 PMC6689455

[55] Peles E, Schreiber S, Sason A, Adelson M. Earning "take-home" privileges and long-term outcome in a methadone maintenance treatment program. J Addict Med. 2011;5(2):92–8. Epub 2011/07/20. doi: 10.1097/ADM.0b013e3181e6ad48 . 21769054

[56] Adelson M, Schreiber S, Sason A, Peles E. Are 2 weeks of "take-home" privileges beneficial for patients’ long-term outcome in a methadone maintenance treatment program? J Addict Med. 2014;8(3):170–5. Epub 2014/01/21. doi: 10.1097/ADM.0000000000000011 . 24440893

[57] Administration DE. Methadone. DEA Diversion Control Division: Drug & Chemical Evaluation Section, 2019.

[58] Jones CM, Baldwin GT, Manocchio T, White JO, Mack KA. Trends in Methadone Distribution for Pain Treatment, Methadone Diversion, and Overdose Deaths—United States, 2002–2014. MMWR Morb Mortal Wkly Rep. 2016;65(26):667–71. Epub 2016/07/09. doi: 10.15585/mmwr.mm6526a2 . 27387857

[59] Treatment CfSA. Methadone-Associated Mortality: Background Briefing Report. Center for Substance Abuse Treatment, Substance Abuse and Mental Health Services Administration, 2004.

[60] Joseph G, Torres-Lockhart K, Stein MR, Mund PA, Nahvi S. Reimagining patient-centered care in opioid treatment programs: Lessons from the Bronx during COVID-19. J Subst Abuse Treat. 2021;122:108219. Epub 20201203. doi: 10.1016/j.jsat.2020.108219 ; PubMed Central PMCID: PMC7833302. 33353790 PMC7833302

[61] Tracy K, Wachtel L, Friedman T. The impact of COVID-19 on opioid treatment program (OTP) services: Where do we go from here? J Subst Abuse Treat. 2021;131:108394. Epub 20210409. doi: 10.1016/j.jsat.2021.108394 ; PubMed Central PMCID: PMC8032399. 34098292 PMC8032399

[62] Nunes EV, Levin FR, Reilly MP, El-Bassel N. Medication treatment for opioid use disorder in the age of COVID-19: Can new regulations modify the opioid cascade? J Subst Abuse Treat. 2021;122:108196. Epub 20201114. doi: 10.1016/j.jsat.2020.108196 ; PubMed Central PMCID: PMC7666540. 33221125 PMC7666540

[63] Kilbourne AM, Switzer G, Hyman K, Crowley-Matoka M, Fine MJ. Advancing Health Disparities Research Within the Health Care System: A Conceptual Framework. American Journal of Public Health. 2006;96(12):2113–21. doi: 10.2105/AJPH.2005.077628 17077411 PMC1698151

[64] Harvey G, Kitson AJIS. PARIHS revisited: from heuristic to integrated framework for the successful implementation of knowledge into practice. 2016;11(1):33. doi: 10.1186/s13012-016-0398-2 27013464 PMC4807546

[65] Swindle T, Johnson SL, Whiteside-Mansell L, Curran GM. A mixed methods protocol for developing and testing implementation strategies for evidence-based obesity prevention in childcare: a cluster randomized hybrid type III trial. Implementation Science. 2017;12(1):90. doi: 10.1186/s13012-017-0624-6 28720140 PMC5516351

[66] Kitson A, Harvey G. Facilitating an evidence-based innovation into practice. Implementing evidence-based practice in healthcare: a facilitation guide. 2015:85.

[67] Institute of Medicine. Improving the Quality of Health Care for Mental and Substance-Use Conditions. Washington, D.C.: National Academies Press; 2006. 20669433

[68] Office of Surgeon General. Facing Addiction in America: The Surgeon General’s Report on Alcohol, Drugs, and Health. Reports of the Surgeon General. Washington (DC): US Department of Health and Human Services; 2016. 28252892

[69] Padwa H, Urada D, Gauthier P, Rieckmann T, Hurley B, Crevecouer-MacPhail D, et al. Organizing Publicly Funded Substance Use Disorder Treatment in the United States: Moving Toward a Service System Approach. J Subst Abuse Treat. 2016;69:9–18. doi: 10.1016/j.jsat.2016.06.010 . 27568505

[70] National Center on Addiction and Substance Abuse at Columbia University. Addiciton medicine: Closing the gap between science and practice author, 2012 June 2012. Report No.

[71] England MJ, Butler AS, Gonzalez ML. Psychosocial interventions for mental and substance use disorders: A framework for establishing evidence-based standards: National Academy Press; 2015. 26203478

[72] McLellan AT, Lewis DC, O’Brien CP, Kleber HD. Drug dependence, a chronic medical illness: implications for treatment, insurance, and outcomes evaluation. Jama. 2000;284(13):1689–95. doi: 10.1001/jama.284.13.1689 . 11015800

[73] O’Grady MA, Lincourt P, Gilmer E, Kwan M, Burke C, Lisio C, et al. How are Substance Use Disorder Treatment Programs Adjusting to Value-Based Payment? A Statewide Qualitative Study. Subst Abuse. 2020;14:1178221820924026. Epub 2020/06/11. doi: 10.1177/1178221820924026 ; PubMed Central PMCID: PMC7252360. 32518481 PMC7252360

[74] O’Grady MA, Lincourt P, Greenfield B, Manseau MW, Hussain S, Genece KG, et al. A facilitation model for implementing quality improvement practices to enhance outpatient substance use disorder treatment outcomes: a stepped-wedge randomized controlled trial study protocol. Implement Sci. 2021;16(1):5. Epub 2021/01/09. doi: 10.1186/s13012-020-01076-x ; PubMed Central PMCID: PMC7789887. 33413493 PMC7789887

[75] Hunter SB, Ober AJ, Paddock SM, Hunt PE, Levan D. Continuous quality improvement (CQI) in addiction treatment settings: design and intervention protocol of a group randomized pilot study. Addiction Science & Clinical Practice. 2014;9(1):4. doi: 10.1186/1940-0640-9-4 24467770 PMC3906762

[76] Hunter SB, Rutter CM, Ober AJ, Booth MS. Building capacity for continuous quality improvement (CQI): A pilot study. Journal of substance abuse treatment. 2017;81:44–52. doi: 10.1016/j.jsat.2017.07.014 28847454 PMC5599160

[77] Quanbeck AR, Madden L, Edmundson E, Ford JH, McConnell KJ, McCarty D, et al. A business case for quality improvement in addiction treatment: evidence from the NIATx collaborative. The journal of behavioral health services & research. 2012;39(1):91–100. doi: 10.1007/s11414-011-9259-6 21918924 PMC3488450

[78] McCarty D, Gustafson D, Capoccia VA, Cotter F. Improving care for the treatment of alcohol and drug disorders. The journal of behavioral health services & research. 2009;36(1):52–60. doi: 10.1007/s11414-008-9108-4 18259871 PMC2642891

[79] Crèvecoeur-MacPhail D, Bellows A, Rutkowski BA, Ransom L, Myers AC, Rawson RA. “I’ve been NIATxed”: Participants’ Experience with Process Improvement. Journal of psychoactive drugs. 2010;42(sup6):249–59. 21138201

[80] Gustafson DH, Quanbeck AR, Robinson JM, Ford JH, 2nd, Pulvermacher A, French MT, et al. Which elements of improvement collaboratives are most effective? A cluster-randomized trial. Addiction. 2013;108(6):1145–57. doi: 10.1111/add.12117 ; PubMed Central PMCID: PMC3651751. 23316787 PMC3651751

[81] McCarty D, Gustafson DH, Wisdom JP, Ford J, Choi D, Molfenter T, et al. The Network for the Improvement of Addiction Treatment (NIATx): enhancing access and retention. Drug Alcohol Depend. 2007;88(2–3):138–45. doi: 10.1016/j.drugalcdep.2006.10.009 ; PubMed Central PMCID: PMC1896099. 17129680 PMC1896099

[82] Fields D, Knudsen HK, Roman PM. Implementation of Network for the Improvement of Addiction Treatment (NIATx) processes in substance use disorder treatment centers. The journal of behavioral health services & research. 2016;43(3):354–65. 25934355 10.1007/s11414-015-9466-7 PMC4630212

[83] Bao Y, Williams AR, Schackman BR. COVID-19 Could Change the Way We Respond to the Opioid Crisis-for the Better. Psychiatr Serv. 2020;71(12):1214–5. Epub 20200812. doi: 10.1176/appi.ps.202000226 ; PubMed Central PMCID: PMC7891848. 32781928 PMC7891848

[84] Bao Y, Li Y, Jeng PJ, Scodes J, Papp MA, Humensky JL, et al. Design of a Payment Decision-Support Tool for Coordinated Specialty Care for Early Psychosis. Psychiatr Serv. 2021;72(2):180–5. Epub 2020/12/04. doi: 10.1176/appi.ps.202000129 ; PubMed Central PMCID: PMC8317229. 33267653 PMC8317229

[85] Hsieh HF, Shannon SE. Three approaches to qualitative content analysis. Qual Health Res. 2005;15(9):1277–88. Epub 2005/10/06. doi: 10.1177/1049732305276687 . 16204405

[86] Bengtsson M. How to plan and perform a qualitative study using content analysis. NursingPlus open. 2016;2:8–14.

[87] Erlingsson C, Brysiewicz P. A hands-on guide to doing content analysis. African journal of emergency medicine. 2017;7(3):93–9. doi: 10.1016/j.afjem.2017.08.001 30456117 PMC6234169

[88] Nevedal AL, Reardon CM, Opra Widerquist MA, Jackson GL, Cutrona SL, White BS, et al. Rapid versus traditional qualitative analysis using the Consolidated Framework for Implementation Research (CFIR). Implementation Science. 2021;16(1):1–12. 34215286 10.1186/s13012-021-01111-5 PMC8252308

[89] Gale RC, Wu J, Erhardt T, Bounthavong M, Reardon CM, Damschroder LJ, et al. Comparison of rapid vs in-depth qualitative analytic methods from a process evaluation of academic detailing in the Veterans Health Administration. Implementation Science. 2019;14(1):11. doi: 10.1186/s13012-019-0853-y 30709368 PMC6359833

[90] Hamilton AB, Finley EP. Qualitative methods in implementation research: An introduction. Psychiatry research. 2019;280:112516. doi: 10.1016/j.psychres.2019.112516 31437661 PMC7023962

[91] Stouten J, Rousseau DM, de Cremer D. Successful organizational change: Integrating the management practice and scholarly literatures. The Academy of Management Annals. 2018;12(2):752–88. doi: 10.5465/annals.2016.0095

[92] Wang SY, Groene O. The effectiveness of behavioral economics-informed interventions on physician behavioral change: A systematic literature review. PLoS One. 2020;15(6):e0234149. Epub 2020/06/05. doi: 10.1371/journal.pone.0234149 ; PubMed Central PMCID: PMC7272062 policies on sharing data and materials. 32497082 PMC7272062

[93] Mostofian F, Ruban C, Simunovic N, Bhandari M. Changing physician behavior: what works? Am J Manag Care. 2015;21(1):75–84. Epub 2015/04/17. . 25880152

[94] Woodward EN, Matthieu MM, Uchendu US, Rogal S, Kirchner JE. The health equity implementation framework: proposal and preliminary study of hepatitis C virus treatment. Implement Sci. 2019;14(1):26. Epub 20190312. doi: 10.1186/s13012-019-0861-y ; PubMed Central PMCID: PMC6417278. 30866982 PMC6417278

[95] Woodward EN, Singh RS, Ndebele-Ngwenya P, Melgar Castillo A, Dickson KS, Kirchner JE. A more practical guide to incorporating health equity domains in implementation determinant frameworks. Implement Sci Commun. 2021;2(1):61. Epub 20210605. doi: 10.1186/s43058-021-00146-5 ; PubMed Central PMCID: PMC8178842. 34090524 PMC8178842

[96] MacKenzie TA, Grunkemeier GL, Grunwald GK, O’Malley AJ, Bohn C, Wu Y, et al. A primer on using shrinkage to compare in-hospital mortality between centers. Ann Thorac Surg. 2015;99(3):757–61. doi: 10.1016/j.athoracsur.2014.11.039 . 25742812

[97] Cohen ME, Ko CY, Bilimoria KY, Zhou L, Huffman K, Wang X, et al. Optimizing ACS NSQIP modeling for evaluation of surgical quality and risk: patient risk adjustment, procedure mix adjustment, shrinkage adjustment, and surgical focus. J Am Coll Surg. 2013;217(2):336–46.e1. Epub 20130428. doi: 10.1016/j.jamcollsurg.2013.02.027 . 23628227

[98] George EI, Ročková V, Rosenbaum PR, Satopää VA, Silber JH. Mortality Rate Estimation and Standardization for Public Reporting: Medicare’s Hospital Compare. Journal of the American Statistical Association. 2017;112(519):15. Epub 30 October 2017. doi: 10.1080/01621459.2016.1276021

[99] Varewyck M, Goetghebeur E, Eriksson M, Vansteelandt S. On shrinkage and model extrapolation in the evaluation of clinical center performance. Biostatistics. 2014;15(4):651–64. Epub 20140508. doi: 10.1093/biostatistics/kxu019 ; PubMed Central PMCID: PMC4173104. 24812420 PMC4173104

[100] Kidorf M, Brooner RK, Dunn KE, Peirce JM. Use of an electronic pillbox to increase number of methadone take-home doses during the COVID-19 pandemic. J Subst Abuse Treat. 2021;126:108328. Epub 20210211. doi: 10.1016/j.jsat.2021.108328 ; PubMed Central PMCID: PMC7876480. 34116819 PMC7876480

[101] Dunn KE, Brooner RK, Stoller KB. Technology-assisted methadone take-home dosing for dispensing methadone to persons with opioid use disorder during the Covid-19 pandemic. J Subst Abuse Treat. 2021;121:108197. Epub 20201124. doi: 10.1016/j.jsat.2020.108197 ; PubMed Central PMCID: PMC7834258. 33357606 PMC7834258

[102] Brooklyn JR, Stothart M, Stunell M, Berman VM, Rylant D, Hanson M. Characterizing the Clinical use of a Novel Video-assisted Dosing Protocol With Secure Medication Dispensers to Reduce Barriers to Opioid Treatment. J Addict Med. 2021. Epub 20210716. doi: 10.1097/adm.0000000000000895 . 34282084

[103] Welsh C, Doyon S, Hart K. Methadone exposures reported to poison control centers in the United States following the COVID-19-related loosening of federal methadone regulations. Int J Drug Policy. 2022;102:103591. Epub 20220120. doi: 10.1016/j.drugpo.2022.103591 ; PubMed Central PMCID: PMC8769878. 35085855 PMC8769878

[104] PASS 2022 Power Analysis and Sample Size Software. Kaysville, Utah, USA: NCSS, LLC; 2022.

[105] Li F, Hughes JP, Hemming K, Taljaard M, Melnick ER, Heagerty PJ. Mixed-effects models for the design and analysis of stepped wedge cluster randomized trials: An overview. Stat Methods Med Res. 2021;30(2):612–39. Epub 2020/07/08. doi: 10.1177/0962280220932962 ; PubMed Central PMCID: PMC7785651. 32631142 PMC7785651

[106] Stroup WW. Generalized linear mixed models: modern concepts, methods and applications. Boca Raton: CRC Press, Taylor & Francis Group; 2013. xxv, 529 pages p.

[107] Brooks ME, Kristensen K, van Benthem KJ, Magnusson A, Berg CW, Nielsen A, et al. glmmTMB Balances Speed and Flexibility Among Packages for Zero-inflated Generalized Linear Mixed Modeling. R J. 2017;9(2):378–400. doi: 10.32614/Rj-2017-066 WOS:000423751200026.

[108] Team RC. R: A language and environment for statistical computing. Vienna, Austria: R Foundation for Statistical Computing; 2021.

[109] Laster LL, Johnson MF. Non-inferiority trials: the ’at least as good as’ criterion. Stat Med. 2003;22(2):187–200. Epub 2003/01/10. doi: 10.1002/sim.1137 . 12520556

[110] Barber JS, Murphy SA, Axinn WG, Maples J. Discrete-time multilevel hazard analysis. Sociol Methodol. 2000;30:201–35. doi: 10.1111/0081-1750.00079 WOS:000166588900006.

[111] Austin PC. A Tutorial on Multilevel Survival Analysis: Methods, Models and Applications. International Statistical Review. 2017;85(2):185–203. doi: 10.1111/insr.12214 29307954 PMC5756088

[112] Steele F. Multilevel discrete-time event history models with applications to the analysis of recurrent employment transitions. Australian & New Zealand Journal of Statistics. 2011;53(1):1–20. doi: 10.1111/j.1467-842X.2011.00604.x

[113] Singer JD, Willett JB. Applied longitudinal data analysis: Modeling change and event occurrence. 1st Edition ed: Oxford University Press; 2003.

[114] Shelley DR, Ogedegbe G, Anane S, Wu WY, Goldfeld K, Gold HT, et al. Testing the use of practice facilitation in a cluster randomized stepped-wedge design trial to improve adherence to cardiovascular disease prevention guidelines: HealthyHearts NYC. Implement Sci. 2016;11(1):88. doi: 10.1186/s13012-016-0450-2 ; PubMed Central PMCID: PMC4932668. 27377404 PMC4932668

[115] Nutting PA, Crabtree BF, Stewart EE, Miller WL, Palmer RF, Stange KC, et al. Effect of facilitation on practice outcomes in the National Demonstration Project model of the patient-centered medical home. Ann Fam Med. 2010;8 Suppl 1:S33–44; S92. doi: 10.1370/afm.1119 ; PubMed Central PMCID: PMC2885723. 20530393 PMC2885723

[116] Solberg LI, Asche SE, Margolis KL, Whitebird RR. Measuring an organization’s ability to manage change: the change process capability questionnaire and its use for improving depression care. Am J Med Qual. 2008;23(3):193–200. doi: 10.1177/1062860608314942 . 18539980

[117] Klein KJ, Sorra JS. The challenge of innovation implementation. The Academy of Management Review. 1996;21(4):1055–80. doi: 10.2307/259164

[118] Klein KJ, Conn AB, Sorra JS. Implementing computerized technology: an organizational analysis. J Appl Psychol. 2001;86(5):811–24. Epub 2001/10/13. doi: 10.1037/0021-9010.86.5.811 . 11596799

[119] Green CA, McCarty D, Mertens J, Lynch FL, Hilde A, Firemark A, et al. A qualitative study of the adoption of buprenorphine for opioid addiction treatment. J Subst Abuse Treat. 2014;46(3):390–401. doi: 10.1016/j.jsat.2013.09.002 ; PubMed Central PMCID: PMC3897203. 24268947 PMC3897203

[120] Palinkas LA, Horwitz SM, Green CA, Wisdom JP, Duan N, Hoagwood K. Purposeful Sampling for Qualitative Data Collection and Analysis in Mixed Method Implementation Research. Administration and policy in mental health. 2015;42(5):533–44. doi: 10.1007/s10488-013-0528-y . 24193818 PMC4012002

[121] Singer JW J.B. Applied Longitudinal Data Analysis: Modeling change and event occurance New York: Oxford University Press 2003.

