# Supplementary material for: Clinics Optimizing MEthadone Take-homes for opioid use disorder (COMET): Protocol for a stepped-wedge randomized trial to facilitate clinic level changes
Source: PLoS One. 2023 Jun 9;18(6):e0286859. doi: 10.1371/journal.pone.0286859 (PMC10256218; doi:10.1371/journal.pone.0286859)
Supplement: S1 File — (DOCX) [file pone.0286859.s002.docx]

**Leveraging Regulatory Flexibility for Methadone Take-Home Dosing to Improve Retention in Treatment for Opioid Use Disorder: A Stepped-Wedge Randomized Trial to Facilitate Clinic Level Changes**

| **Principal Investigator:** | Dr. Charles Neighbors  NYU Langone Health  Department of Population Health  Charles.Neighbors@nyulangone.org  646-501-3879 |
| --- | --- |
| **NYULMC Study Number:** | S22-00892 |
| **Funding Sponsor:** | National Institutes of Health |

# Statement of Compliance

This study will be conducted in accordance with the Code of Federal Regulations on the Protection of Human Subjects (45 CFR Part 46), 21 CFR Parts 50, 56, 312, and 812 as applicable, any other applicable US government research regulations, and institutional research policies and procedures. The International Conference on Harmonisation (“ICH”) Guideline for Good Clinical Practice (“GCP”) (sometimes referred to as “ICH-GCP” or “E6”) will be applied only to the extent that it is compatible with FDA and DHHS regulations. The Principal Investigator will assure that no deviation from, or changes to the protocol will take place without prior agreement from the sponsor and documented approval from the Institutional Review Board (IRB), except where necessary to eliminate an immediate hazard(s) to the trial participants. All personnel involved in the conduct of this study have completed Human Subjects Protection Training.

**Table of Contents**

Statement of Compliance ii

Protocol Summary 6

Schematic of Study Design 9

1 Key Roles 10

2 Introduction, Background Information and Scientific Rationale 10

2.1 Background Information and Relevant Literature 10

2.2 Rationale 11

2.3 Potential Risks & Benefits 14

2.3.1 Known Potential Risks 14

2.3.2 Adequacy of Protection Against Risks 16

2.3.3 Known Potential Benefits 17

3 Objectives and Purpose 18

3.1 Primary Objective 19

4 Study Design and Endpoints 20

4.1 Description of Study Design 20

4.2 Study Endpoints 20

4.2.1 Primary Study Endpoints 20

5 Study Enrollment and Withdrawal 20

5.1 Inclusion Criteria 21

5.2 Exclusion Criteria 21

5.3 Vulnerable Subjects 21

5.4 Strategies for Recruitment and Retention 21

5.5 Duration of Study Participation 22

5.6 Participant Withdrawal or Termination 22

6 Study Procedures and Schedule 22

6.1 Study Procedures/Evaluations 22

6.1.1 Year 1 22

6.1.2 Years 2-5 24

6.2 Study Schedule 25

7 Assessment of Safety 26

7.1 Investigator Reporting 26

7.1.1 Safety and Adverse Events 26

7.2 Notifying the IRB 26

7.3 Study Oversight 27

8 Statistical Methods 27

8.1 Year 1 Analyses 27

8.2 Years 2-5 Analyses 28

8.2.1 Analysis of the effect of the OTP Intervention on THD, retention in care and adverse events 28

8.2.2 Analysis of qualitative interviews with OTP leadership, staff, and clients 29

8.2.3 Analysis of variation in THD associated with race/ethnicity 30

8.3 Statistical Power 30

9 Ethics/Protection of Human Subjects 31

9.1 Ethical Standard 31

9.2 Institutional Review Board 31

9.3 Informed Consent Process 31

9.3.1 Consent/Assent and Other Informational Documents Provided to Participants 31

9.3.2 Waivers 31

9.4 Consent Procedures and Documentation 31

9.5 Participant and Data Confidentiality 32

10 Data Handling and Record Keeping 32

10.1 Data Collection, Confidentiality and Management Responsibilities 32

10.2 Study Records Retention 33

10.3 Protocol Deviations 33

10.3.1 Reporting Process 34

10.4 Publication and Data Sharing Policy 34

11 Study Finances 35

11.1 Funding Source 35

11.2 Costs to the Participant 35

11.3 Participant Reimbursements or Payments 35

12 Study Administration 35

12.1 Study Leadership 35

12.2 DSMP 35

12.3 Rationale for Multiple Principal Investigators 36

12.3.1 Communication among Principal Investigators 36

12.3.2 Plans for Resolution of Conflicts 37

12.3.3 Change in PI Location 37

13 Conflict of Interest Policy 37

14 References 38

**List of Abbreviations**

| CDS | Client Data System |
| --- | --- |
| CIMU | Conflict of Interest Management Unit |
| DEA | Drug Enforcement Agency |
| DHHS | Department of Health and Human Services |
| DSMB | Data and Safety Monitoring Board |
| ED | Emergency Department |
| FDA | Federal Drug Administration |
| GCP | Good Clinical Practice |
| GLMM | General Linear Mixed Model |
| HEIF | Health Equity Implementation Framework |
| HIPAA | Health Insurance Portability and Accountability Act |
| ICH | International Conference on Harmonisation |
| iPARIHS | Integrated- Promoting Action on Research Implementation in Health Services |
| IRB | Institutional Review Board |
| MPI | Multiple Principal Investigators |
| NIH | National Institutes of Health |
| NYSDOH | New York State Department of Health |
| OASAS | Office of Addiction Services and Supports |
| OTP | Opioid Treatment Program |
| OUD | Opioid Use Disorder |
| PHE | Public Health Emergency |
| PWOUD | People with Opioid Use Disorders |
| PI | Principal Investigator |
| SAMHSA | Substance Abuse and Mental Health Services Administration |
| SDM | Shared Decision Making |
| SW-RCT | Stepped-Wedged Randomized Controlled Trial |
| THD | Take-Home Dosing |
|  |  |
|  |  |
|  |  |
|  |  |
|  |  |
|  |  |

# Protocol Summary

| Title | Leveraging regulatory flexibility for methadone take-home dosing to improve retention in treatment for opioid use disorder: A stepped-wedge randomized trial to facilitate clinic level changes |
| --- | --- |
| Brief Summary | Regulatory changes made during the COVID-19 public health emergency (PHE) that relaxed criteria for take- home dosing (THD) of methadone offer an opportunity to improve retention in care with a lifesaving treatment. Methadone is a highly effective medication for treating opioid use disorders (OUD) that is provided in opioid treatment programs (OTPs). Yet, longstanding regulatory restrictions limit the availability of methadone as well as create demands that heavily burden clients by requiring frequent visits to clinics. The rationale for these regulations is to safeguard against diversion and overdoses from methadone. Yet, the history and application of methadone regulations stem from stigmatized and racist notions of people with OUD. Most OTPs are located within communities with predominantly Black/African American or Latinx populations. Consequently, Black/African American and Latinx individuals have greater access to methadone than other, less restricted, medications for OUD. Within OTPs, Black/African American and Latinx individuals are less likely to receive adequate dosing levels of methadone and have lower retention than non-Hispanic White clients. More flexible THD may help address disparities in care. Currently, there is a national debate about balancing safety concerns over more flexible THD against the benefits of client retention and quality of life. Low offering of THD in many OTPs suggests a need for new data-driven interventions to encourage changes in engrained clinical workflows and long-standing stigmatizing beliefs about OUD clients. OTP leadership and staff express concern about misapplying regulatory flexibility, of iatrogenic effects of greater THD, and about legal liability from overdoses or diversion. Finally, financial concerns mount for organizations that have long based their business models on billing for frequent in-person medication dispensing. This project stems from a well-established academic-public partnership in New York State between the Office of Addiction Services and Supports (OASAS) and research collaborators from New York University, Cornell University, and the University of Connecticut. We propose a two-part project to develop then test a multidimensional OTP intervention to address clinical decision making, regulatory confusion, legal liability concerns, capacity for clinical practice change, and financial barriers to THD. The intervention will include OTP THD specific dashboards drawn from multiple State databases. The approach will be informed by the Health Equity Implementation Framework. In year 1, we will employ an explanatory sequential mixed method design to combine analysis of large state administrative databases—Medicaid, treatment registry, THD reporting—with qualitative interviews to refine the intervention. In years 2-5, we will conduct a stepped-wedge trial with 36 OTPs (~10,800 Medicaid clients/yr) randomized to 6 cohorts of a six-month long clinic-level intervention over three years. The trial will test the effects of the intervention on 1) THD; 2) retention in care; and 3) adverse healthcare events. We will specifically examine the effects of the intervention for Black/African American and Latinx clients. |
| Methods | - Explanatory Sequential Mixed-Methods study with database analysis and qualitative interviews to refine intervention - Randomized Stepped-Wedge Trial with sic-month clinic-level intervention |
| Objectives | **Year 1: Hone the Intervention**  Aim 1. Analyze administrative data to identify factors associated with OTP variation in THD practices and categorize clinics by THD flexibility.  Aim 2. Conduct qualitative interviews with leadership and staff of 10 OTPs (5 high and 5 low THD flexibility) on clinical and organizational factors affecting take-home dosing decisions.  Aim 3. Complete development of the multidimensional OTP intervention.  **Years 2-5: Stepped-Wedge Trial**  Aim 4. Test the effects of the intervention on THD, retention in care, and adverse events. Hypotheses 1-3: The intervention will increase H1) rates of 7-day, 14-day, and 28-day THD, H2) retention in care, yet H3) not change the rate of adverse events (e.g., emergency department visits, hospitalizations, all-cause mortality).  Aim 5. Conduct qualitative interviews with OTP leadership, staff, and clients on attitudes, experiences and behaviors related to the intervention.  Aim 6. Using mixed methods, explore variation in THD associated with race and ethnicity. |
| Methodology | **Year 1:** We will implement an explanatory sequential mixed methods design^[[1]](#endnote-1)^, ^[[2]](#endnote-2)^ that combines analyses of administrative data with qualitative data collection to examine OTP organizational factors associated THD. We will combine data from four sources (described further below). An OASAS registry of OTP treatment episodes—the Client Data System (CDS)—will provide socio-demographic and clinical information on clients. An OASAS THD monitoring database will provide clinic aggregate data on THD practices. Based on findings from analyses of these data, we will select 10 clinics for qualitative interviews with staff members to explore further clinic level factors associated with THD practices as well as technical assistance needs to implement more flexible THD.  **Years 2-5:** We propose an embedded mixed methods study in which we will employ a stepped-wedged randomized controlled trial with 36 OTPs to test whether the intervention a) increases THD, b) increases retention in care, and c) has any effect on adverse events (i.e., emergency department visits, hospitalizations, mortality). OTPs will be randomized across 6 sequential steps between years 2 and 4. The examination of outcomes will benefit from access to rich sources of data described below. Qualitative data collection with staff and patients will be embedded within the trial to examine experiences with the new THD protocols and to inform quantitative findings. |
| Study Duration | 5 years |
| Participant Duration | 6-month interventions for 6 cohorts of 6 clinics each over 3 years |
| Population | Stepped-Wedged Trial: During year 1 of the study, we will draw potential sites from OTPs with high and low THD flexibility. We anticipate to recruit 10 OTPs (5 high and 5 low THD flexibility) on clinical and organizational factors affecting take-home dosing decisions based on our quantitative study during year 1. After the consent from the OTPs, we will recruit leadership (e.g., program director, clinical supervisor, etc.) and frontline treatment staff (e.g., addiction physicians, hospital nursing and social work leaders, hospitalists, administrative staff, etc.) for one-on-one interviews. In years 2-4, we plan to conduct a post-intervention survey with providers/staff and interviews with providers and patients from 36 OTPs. |
| Study Sites | NYU Langone Health  Weil Cornell Medical College  New York State (NYS) Office of Addiction Services and Supports (OASAS)  University of Connecticut |
| Number of participants | The qualitative research will include data from approximately 174 addiction treatment staff and 144 patients being interviewed about take-home dosing practice changes after the intervention has been implemented. |
| Study Intervention | Using a stepped-wedge randomized controlled trial, the study will test whether a clinic-level multidimensional intervention conducted in 36 opioid treatment programs (OTPs) will improve clinical decision making, regulatory confusion, legal liability concerns, capacity for clinical practice change, and financial barriers to take- home dosing (THD) for methadone as compared to treatment as usual. |
| Statistical Analysis | This two-part proposal will develop, implement, and test protocols and tools for facilitating adoption of greater THD flexibility in OTPs. It will evaluate the impact of the intervention on acute healthcare events (i.e., emergency department visits and hospitalizations) as well as all-cause mortality. *We choose all- cause mortality for measurement concerns explained below. Y*ear 1 of the project will involve an explanatory sequential mixed methods design, starting with extensive analyses of state administrative data (Medicaid, treatment registry, OTP THD reporting) to examine patterns of THD as well as individual and organizational level factors associated with greater flexibility of THD. We will then interview staff at OTPs to obtain qualitative data on factors affecting THD. Based on the results, we will hone the OTP intervention described below. In years 2-5 of the project, we will employ a stepped-wedge randomized controlled trial to test the effectiveness of the intervention among 36 OTPs that are enrolled in six cohorts at staggered enrollment time points. The trial will employ an embedded mixed methods design to study the intervention impact as well as gain important tacit knowledge that will inform future dissemination. |

# Schematic of Study Design


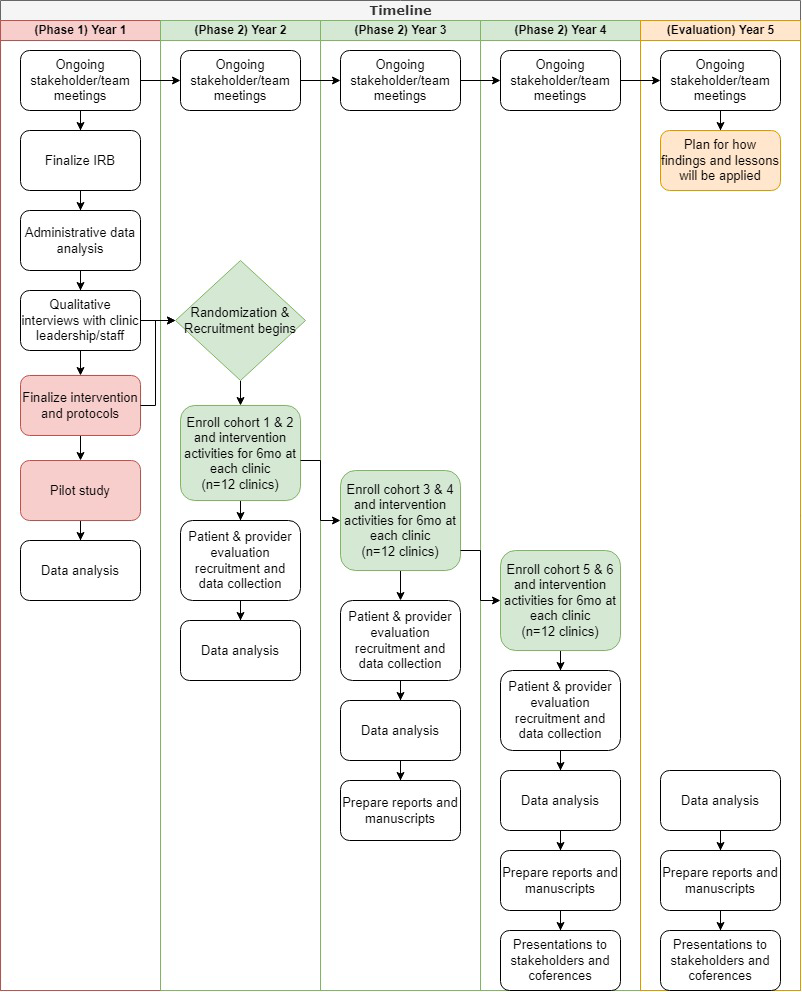


# Key Roles

| **Principal Investigator** | Charles Neighbors  NYU Langone Health  Department of Population Health  Charles.Neighbors@nyulangone.org |
| --- | --- |
| **Sub-Investigators** | Sugy Choi  NYU Langone Health  Department of Population Health  Sugy.Choi@nyulangone.org |
| **Study Team Members:** | Kamila Kiszko  NYU Langone Health  Department of Population Health  Kamila.Kiszko@nyulangone.org  Sueun Hong  NYU Langone Health  Department of Population Health  Sueun.Hong@nyulangone.org |

# Introduction, Background Information and Scientific Rationale

## Background Information and Relevant Literature

Regulatory changes made during the COVID-19 public health emergency (PHE) that relaxed criteria for take- home dosing (THD) of methadone offer an opportunity to improve retention in care with a lifesaving treatment. Methadone is a highly effective medication for treating opioid use disorders (OUD) that is provided in opioid treatment programs (OTPs). Yet, longstanding regulatory restrictions limit the availability of methadone as well as create demands that heavily burden clients by requiring frequent visits to clinics. The rationale for these regulations is to safeguard against diversion and overdoses from methadone. Yet, the history and application of methadone regulations stem from stigmatized and racist notions of people with OUD. Most OTPs are located within communities with predominantly Black/African American or Latinx populations. Consequently, Black/African American and Latinx individuals have greater access to methadone than other, less restricted, medications for OUD. Within OTPs, Black/African American and Latinx individuals are less likely to receive adequate dosing levels of methadone and have lower retention than non-Hispanic White clients. More flexible THD may help address disparities in care.

Currently, there is a national debate about balancing safety concerns over more flexible THD against the benefits of client retention and quality of life. Studies have not found increased overdoses under more flexible THD rules during the PHE; however, longer term studies are needed to understand best THD practices and outcomes. In New York, the Office of Addiction Services and Supports (OASAS) vigorously encouraged OTPs during the PHE to increase THD flexibility by highlighting clinical benefits, clarifying regulatory rules, monitoring OTP THD practices, and offering a new Medicaid payment to incentivize THD. The New York OTP system is large, serving >38,000 individuals each year. The state provides robust support to OTPs, with 83% of clients covered by Medicaid. New York data from the first year of the PHE show large variability across OTPs in the application of the more flexible THD criteria. Low offering of THD in many OTPs suggests a need for new data- driven interventions to encourage changes in engrained clinical workflows and long-standing stigmatizing beliefs about OUD clients. OTP leadership and staff express concern about misapplying regulatory flexibility, of iatrogenic effects of greater THD, and about legal liability from overdoses or diversion. Finally, financial concerns mount for organizations that have long based their business models on billing for frequent in-person medication dispensing. Buoyed by lack of evidence of adverse consequences from greater THD during the PHE, OASAS remains committed to maintaining increased THD flexibility in the system and will apply for federal waivers to extend the regulatory relaxation for THD indefinitely.

We propose a two-part project to develop then test a multidimensional OTP intervention to address clinical decision making, regulatory confusion, legal liability concerns, capacity for clinical practice change, and financial barriers to THD. The intervention will include OTP THD specific dashboards drawn from multiple State databases. The approach will be informed by the Health Equity Implementation Framework. In year 1, we will employ an explanatory sequential mixed method design to combine analysis of large state administrative databases—Medicaid, treatment registry, THD reporting—with qualitative interviews to refine the intervention. In years 2-5, we will conduct a stepped-wedge trial with 36 OTPs (~10,800 Medicaid clients/yr) randomized to 6 cohorts of a six-month long clinic-level intervention over three years. The trial will test the effects of the intervention on 1) THD; 2) retention in care; and 3) adverse healthcare events. We will specifically examine the effects of the intervention for Black/African American and Latinx clients.

## Rationale

**Opioid Use Disorders.** The United States is experiencing an unrelenting epidemic of opioid use disorder (OUD).^[[3]](#endnote-3)^, ^[[4]](#endnote-4)^, ^[[5]](#endnote-5)^, ^[[6]](#endnote-6)^ OUD is associated with high mortality, morbidity, and low remission.^[[7]](#endnote-7)^, ^[[8]](#endnote-8)^, ^[[9]](#endnote-9)^, ^[[10]](#endnote-10)^, ^[[11]](#endnote-11)^, ^[[12]](#endnote-12)^, ^[[13]](#endnote-13)^ Opioid related overdose rates continue to rise at an alarming rate.^[[14]](#endnote-14)^, ^[[15]](#endnote-15)^ The increase is disproportionately affecting Black and Latinx individuals, laying bare disparities in the effect of the epidemic on communities of color.^[[16]](#endnote-16)^, ^[[17]](#endnote-17)^, ^[[18]](#endnote-18)^, ^[[19]](#endnote-19)^, ^[[20]](#endnote-20)^, ^[[21]](#endnote-21)^, ^[[22]](#endnote-22)^, ^[[23]](#endnote-23)^

**Methadone.** Methadone is a highly effective treatment for OUD that has been available since the 1960s.^[[24]](#endnote-24)^, ^[[25]](#endnote-25)^, ^[[26]](#endnote-26)^, ^[[27]](#endnote-27)^, ^[[28]](#endnote-28)^, ^[[29]](#endnote-29)^, ^[[30]](#endnote-30)^ In the United States, methadone is provided through clinics called opioid treatment programs (OTPs) that are highly regulated by the Drug Enforcement Agency (DEA), the Substance Abuse and Mental Health Services Administration (SAMHSA), state and local governments. The regulations and the OTP system that has emerged since the Vietnam War era reflect stigmatizing and racist notions of people with OUD (PWOUD) that underscore clinical practices to this date.^[[31]](#endnote-31)^, ^[[32]](#endnote-32)^, ^[[33]](#endnote-33)^, ^[[34]](#endnote-34)^, ^[[35]](#endnote-35)^, ^[[36]](#endnote-36)^ The putative rationale for the restrictive regulations are to mitigate risk of diversion or overdose from methadone;^[[37]](#endnote-37)^ however, the effect has been to limit access and place a heavy burden on clients that one client called “liquid handcuffs.”^[[38]](#endnote-38)^

**Research on OTP Quality of Care.** Results from several prior NIDA-funded studies show that the nation’s OTPs often fail to provide clients with evidence-based treatment and prevention services, including adequate doses of methadone; HIV and HCV testing; mental health services; and critical social services such as childcare and transportation.^[[39]](#endnote-39)^, ^[[40]](#endnote-40)^, ^[[41]](#endnote-41)^, ^[[42]](#endnote-42)^, ^[[43]](#endnote-43)^, ^[[44]](#endnote-44)^, ^[[45]](#endnote-45)^ Further, results from prior studies show that variation in the provision of evidence-based services is associated with key organizational characteristics of OTPs; specifically, OTPs characterized by high ratios of clients to staff, managerial and staff support for abstinence models of care, and for-profit ownership are less likely than others to provide evidence-based services. In general, OTPs are under-resourced, culturally rigid, and highly-regulated. As a result, these programs have low capacity for much-needed innovation.

**Racial/Ethnic Disparities.** OTPs are predominantly located in low-income communities and more likely to be in neighborhoods that are primarily Black/African American or Latinx.^[[46]](#endnote-46)^, ^[[47]](#endnote-47)^, ^[[48]](#endnote-48)^ Black/African American and Latinx PWOUD have less access than non-Latinx White PWOUD to other medications for OUD that have fewer regulatory restrictions that impose high burden on clients.^[[49]](#endnote-49)^, ^[[50]](#endnote-50)^, ^[[51]](#endnote-51)^ Although Black/African American and Latinx PWOUD are less likely than non-Latinx White PWOUD to receive any pharmacotherapy, they are more likely to receive methadone than other medications.^[[52]](#endnote-52)^, ^[[53]](#endnote-53)^,^[[54]](#endnote-54)^, ^[[55]](#endnote-55)^, ^[[56]](#endnote-56)^, ^[[57]](#endnote-57)^ Within OTPs, Black/African American and Latinx PWOUD suffer from poorer quality of care relative to non-Latinx White PWOUD, being more likely to receive sub-optimal therapeutic doses of methadone.^[[58]](#endnote-58)^, ^[[59]](#endnote-59)^, ^[[60]](#endnote-60)^ As a consequence, they have shorter retention in care and lower levels of successful treatment completion.^[[61]](#endnote-61)^, ^[[62]](#endnote-62)^, ^[[63]](#endnote-63)^, ^[[64]](#endnote-64)^, ^[[65]](#endnote-65)^ Notably, Black/African American PWOUD are more likely to be discharged from treatment if they have a positive toxicology test than non-Latinx White PWOUD.^[[66]](#endnote-66)^

**Pre-COVID-19 Take-Home Dosing (THD).** Prior to the COVID-19 federal public health emergency (PHE), SAMHSA regulations required the majority of OTP clients to visit clinics almost daily to monitor their methadone dosing.^[[67]](#endnote-67)^, ^[[68]](#endnote-68)^ Clients could eventually earn privileges for THD that would allow for self-administration but the bar was high. A client needed to be in treatment for 9 months before being eligible for 7-day THD and 12 months before being able to have 14-day THD. In addition to the time in treatment criteria, clients needed to not have evidence of other substance use—including alcohol and cannabis, be ‘compliant’ with treatment, and be relatively high functioning in other life domains.^[[69]](#endnote-69)^, ^[[70]](#endnote-70)^, ^[[71]](#endnote-71)^, ^[[72]](#endnote-72)^, ^[[73]](#endnote-73)^, ^[[74]](#endnote-74)^ The 8-point criteria for compliance and ‘stability’ were not defined in detail and left open to OTP medical staff clinical discretion. Staff generally defaulted to risk- averse interpretations that were often not aligned with objective data.^[[75]](#endnote-75)^ As a result, clients were subject to burdensome and demeaning treatment that created barriers to employment or improvements in other domains of life functioning.^[[76]](#endnote-76)^, ^[[77]](#endnote-77)^, ^[[78]](#endnote-78)^, ^[[79]](#endnote-79)^ The barriers are particularly troublesome for PWOUD who live in rural areas or with long travel distances to OTPs.^[[80]](#endnote-80)^, ^[[81]](#endnote-81)^, ^[[82]](#endnote-82)^, ^[[83]](#endnote-83)^ Notably, Black/African American clients are subject to more restrictive interpretations of the criteria than non-Latinx White clients.^[[84]](#endnote-84)^

There is little evidence to support the notion that these levels of THD restrictions are needed to reduce overdoses or large-scale diversion of methadone. Cross-national comparisons with other countries that have less restrictive regulations don’t show higher mortality or diversion than the United States.^[[85]](#endnote-85)^, ^[[86]](#endnote-86)^, ^[[87]](#endnote-87)^, ^[[88]](#endnote-88)^, ^[[89]](#endnote-89)^, ^[[90]](#endnote-90)^, ^[[91]](#endnote-91)^ Within the United States, DEA reports show little evidence of large-scale diversion of methadone dispensed through OTPs and recent studies show that mortality and diversion have been more strongly associated with methadone prescribed for pain management than for OUD.^[[92]](#endnote-92)^, ^[[93]](#endnote-93)^, ^[[94]](#endnote-94)^

**COVID-19 Regulatory Changes.** In response to the PHE, SAMHSA gave states a temporary option to request a waiver to allow greater flexibility of THD for clients. The new federal rules shifted from time-in- treatment requirements to clinical judgement regarding patient stability. The new rules allow medical staff to classify clients into two categories that would be eligible for THD, “stable” and “less-stable.” Stable clients would be eligible for a 28-day supply of THD, less-stable clients could receive up to 14 days. Recently, SAMHSA issued an extension of the COVID-19 THD waiver option for states indefinitely.^[[95]](#endnote-95)^

Uptake by states of the new waiver option has been uneven. Not all states applied for the waiver,^[[96]](#endnote-96)^, ^[[97]](#endnote-97)^ which reflected the variability in local regulatory approaches.^[[98]](#endnote-98)^ The response by OTP senior leadership and staff has also been mixed. While some have highlighted the benefits to clients of greater flexibility in THD, others have expressed concern about the potential harm to clients from unsupervised dosing that may result in overdoses or diversion or program legal liability from potential misuse.^[[99]](#endnote-99)^, ^[[100]](#endnote-100)^, ^[[101]](#endnote-101)^, ^[[102]](#endnote-102)^, ^[[103]](#endnote-103)^, ^[[104]](#endnote-104)^

**Despite coinciding with a period of spikes in opioid related overdose deaths, there is no evidence that mortality associated with greater THD was associated with more overdoses from methadone.^[[105]](#endnote-105)^, ^[[106]](#endnote-106)^, ^[[107]](#endnote-107)^, ^[[108]](#endnote-108)^, ^[[109]](#endnote-109)^, ^[[110]](#endnote-110)^** A recent large and robust study from Ontario, Canada, found that more flexible THD **increased retention** in care yet wasn’t associated with increased mortality.^[[111]](#endnote-111)^

**New York State Response.** New York State was quick to apply for a federal waiver to allow greater THD flexibility. The State’s regulatory agency, Office of Addiction Services and Supports (OASAS), worked closely with local government units and OTP provider groups to encourage flexibility in THD. OASAS provided regulatory guidance and instituted a THD reporting requirement that allowed for monitoring and feedback to OTPs. In addition, the State adopted new bundled payment options to incentivize extended THD. The bundles provide a robust rate that exceeds the standard in-clinic daily administration rate as well as supports counseling services via telemedicine.

**There has been little evidence of adverse events in New York from more flexible THD.** Publications from two large OTP providers in New York City have reported positive response from clients, general support from OTP staff, and no evidence of significant increases in overdose or diversion.^[[112]](#endnote-112)^, ^[[113]](#endnote-113)^ **OASAS remains committed to maintaining its waiver indefinitely and encouraging OTPs to allow more THD.**

**Rigor of Prior Research.** Methadone is a strongly effective treatment for OUD but regulatory restrictions reduce access and retention. Black/African American and Latinx PWOUD are impacted disproportionately by these restrictions. To date, there is no substantive evidence that THD restrictions provide sufficient protections from any iatrogenic effects of methadone treatment that would outweigh the benefits to clients of greater flexibility. One recent rigorous study from Canada found greater THD was associated with improved retention in care but not increased mortality.^[[114]](#endnote-114)^ In New York, as elsewhere, the greater flexibility for THD during the PHE has been embraced by OASAS with little evidence of untoward effects. Yet, the adoption by OTPs across the state has been uneven. Research to date finds that OTPs are hampered by organizational cultures that have limited capacity for innovation or adoption of new practices. **There is a pressing need for research to study the long-term effects of the new PHE THD rules as well as to test data-driven interventions to promote more effective adoption by OTPs.^[[115]](#endnote-115)^, ^[[116]](#endnote-116)^ We propose a two-part project to develop and test a multidimensional intervention for OTPs that leverages information from large State administrative data.**

**Research Framework.** We draw from the Health Equity Implementation Framework (HEIF) to guide the development and study of an intervention to increase THD among OTPs. HEIF integrates two research frameworks for conducting implementation studies of interventions targeting underserved populations: 1) the Kilbourne framework for health disparities research and 2) the Integrated Promoting Action on Research Implementation in Health Services (i-PARIHS) framework. The Kilbourne framework focuses on historical, cultural, and contextual factors that affect client-provider encounters.^[[117]](#endnote-117)^ i-PARIHS posits that optimal implementation occurs when practice facilitation promotes the acceptance and use of a new practice innovation by tailoring it to the recipient’s specific needs.^[[118]](#endnote-118)^, ^[[119]](#endnote-119)^, ^[[120]](#endnote-120)^Facilitators are the active ingredient that help navigate individuals and teams through complex change processes by addressing a) the innovation’s degree of fit within the existing practice, b) the motivations, beliefs, goals, characteristics, and resources of the intervention recipients, and c) the inner and outer context in terms of leadership support, culture, past innovation experiences, the learning environment, organizational priorities, capacity for change, regulatory/policy drivers, incentives/mandates, and system stability/instability.

**Facilitating Change Management in OTPs.** Multiple systemic reviews have concluded addiction treatment in the United States has large gaps in quality of care and limited capacity for clinical program improvement.^[[121]](#endnote-121)^, ^[[122]](#endnote-122)^, ^[[123]](#endnote-123)^, ^[[124]](#endnote-124)^, ^[[125]](#endnote-125)^, ^[[126]](#endnote-126)^ Notably, the workforce has limited levels of education or professional training to implement process change initiatives to improve clinical outcomes.^[[127]](#endnote-127)^, ^[[128]](#endnote-128)^,^[[129]](#endnote-129)^, ^[[130]](#endnote-130)^ Because of this limitation, the proposed intervention will draw from our current research^[[131]](#endnote-131)^ based on principles of process improvement and the iPARIHS framework to coach OTPs on changing protocols and workflows to adopt more flexible THD.^[[132]](#endnote-132)^, ^[[133]](#endnote-133)^, ^[[134]](#endnote-134)^, ^[[135]](#endnote-135)^, ^[[136]](#endnote-136)^, ^[[137]](#endnote-137)^, ^[[138]](#endnote-138)^, ^[[139]](#endnote-139)^

**Financial Guidance.** One obstacle to adopting new THD practices is uncertainty by the senior leadership of OTPs regarding the financial viability of any change that deviates from established billing practices.^[[140]](#endnote-140)^ Although New York instituted new bundle rates to incentivize THD during the PHE, the low and varied uptake suggests that OTP leadership need financial decision support to increase understanding and acceptance of these new rates. Dr. Bao (MPI of this project) has recent experience developing user-friendly financial assessment tools for mental health programs.^[[141]](#endnote-141)^, ^[[142]](#endnote-142)^ As part of this project, we will use State administrative data to develop a net revenue tool for OTPs then provide a decision-support tool to OTP leadership that allows for revenue/cost projections conditional on target THD practices.

## Potential Risks & Benefits

### Known Potential Risks

The risks to the human subjects in the qualitative study are minimal. Participants in the qualitative analyses will be staff members of the non-NYU affiliated outpatient clinics or agencies, that are not engaged in HSR, identified through the quantitative analysis. All questions will be about organizational characteristics and work protocols. The risks to the participants in the qualitative study are minimal. They are limited to a) breach of confidentiality related to participation and b) discomfort and stress related to sharing their experiences. The study team will implement protections to minimize that risk. First, interview participants will be informed that their participation in this study is voluntary and that they may withdraw at any time. All participants will have the right to refuse participation. Participation in this study will have no impact on employment status or damaging impact on reputation or relationships. During interviews, we will only use first names and if identifying information is shared during the interviews, it will be redacted during transcription. The research team will not be able to track back to individual’s names and affiliation after transcription process.

We plan to obtain verbal consent prior to conducting the interview. The team has decided to obtain verbal consent as consent will be obtained immediately before interview begins. The key information sheet and verbal consent script will be provided to participants prior to the interview and the verbal consent will be read to participants verbally. Verbal consent reduces unnecessary burden on the participants; therefore, we have chosen to obtain verbal consent over e-consents. Participants are always given permission to not answer questions with which they feel uncomfortable. Data collected at the staff level will be non-invasive and similar to what they would experience in the course of their treatment provision or daily work. Participation will not affect daily work and supervisor or clinic staff at respective clinics will not be informed about who is participating in this study. We will not share any information from the interviews with the clinic or staff unless in aggregate, de-identified form. The probability and magnitude of harm or discomfort for the interviewees anticipated in the study are not greater in and of themselves than those ordinarily encountered in daily life. Records of participation in this study will not be connected to residential facility staffs’ employment record. Confidentiality of the participants will be protected in several ways. All participants will receive an identifying number for coding and analyzing data. Identifying code numbers will be kept in a separate file from completed transcripts. Data will be reported in clinic-level format only. Any other potentially identifying information will also be removed (e.g., specific information about unusual characteristics or events that may be known in the community). All data will be kept in a locked file and will be accessible only to the research team. Participants’ names and identifying characteristics will not be used outside of the NYU site.

Interviews will be completed over a secure passcode protected Zoom. We will be audio-recording the interview. Both an audio and video file are automatically created by Zoom when recording, but the video file will be immediately destroyed following the interview and only the audio file will be retained for transcription purposes. The audio file will be downloaded onto secure folders using encrypted laptops. The interviews will be transcribed and saved electronically in encrypted files in a restricted secure folder. Recordings will be deleted immediately after transcription. Prior to transcription, recordings will be saved onto a password protected folder on a secure network and only staff doing transcription will have access to these. Field notes will be completed immediately and transcriptions will be completed as soon as possible after the interviews are conducted to protect confidentiality. In transcripts, participants will be identified using a participant code only. The interview, including completing a short demographic questionnaire, will last approximately 1 hour. Evening times will be offered to minimize interference with work schedules. Additionally, participants may decline to participate in the qualitative interview. Interviewees may feel obliged to complete the questionnaire and the interview if recommended in their organization. We will not be reporting back to leadership on participants therefore they will remain unaware of who agreed to participate and who did not. There is minimal risk of coercion or undue influence over the identification and recruitment process. However, participants will undergo a verbal consenting procedure, and during this consenting procedure, participants will be informed participation is voluntary. Additionally, they will be reminded that they may withdraw from the study at any time.

While we acknowledge the following risks and mitigation procedures, we note that the study risks are minimal. We will implement robust protocols for protecting participants’ privacy and well-being.

Our research will not reveal the names of the participating treatment programs or participants. We will not be collecting names of participants for the survey. In terms of the interviews, the research team will not be able to track back to individual’s names and affiliation after transcription process. The participants will be identified using their role and whether they worked in a low or a high-performing program or whether they live in or outside of New York City (NYC) area. (e.g. Nurse 1, Low; Patient living in NYC, Low)

Time burden is another potential risk but we will try to reduce the burden of participants by only asking necessary questions. The interview, including a short demographic questionnaire, will last approximately 1 hour. Evening times will be offered to minimize interference with work schedules. Additionally, participants may decline to participate in the qualitative interview.

Interviewees may feel obliged to complete the questionnaire and the interview. However, during initial consent, participants will be informed participation is voluntary. Additionally, they will be reminded that they may withdraw from the study at any time.

### Adequacy of Protection Against Risks

#### Administrative Data

No consent will be sought for this data, it is a pre-existing data source.

All data provided will be kept confidential by the NYU research staff. All information contained in research documents and materials will be accessible only to members of the NYU research team who will follow the principle of minimum necessary access to achieve study aims. NYU maintains a robust infrastructure to ensure HIPAA compliance and in the full spirit of protecting human subjects' privacy and welfare. Regarding assessment data and other records, NYU study investigators have an established set of procedures designed to ensure the confidentiality of study data. All staff at NYU who participate in research with human subjects are required to complete regularly scheduled training on the protection of human subjects, including confidentiality. Additionally, the NYU research staff will be trained on and strictly monitored for adherence to federal guidelines for maintaining the privacy and confidentiality of participants throughout the research process. All study databases will be stored within the NYU HEAL, which has robust hardware and software safeguards to secure the data. These safeguards include physical barriers to access to computers containing the data, data encryption in storage, and limited access set by the principal investigator and requiring multi-factor authentication.

#### Interview Participants

*Informed Consent and Assent for Interview Participants*

After low- and high-performing treatment programs have been chosen, one of the qualitative study group leaders (not the research assistant) will arrange a meeting to discuss the study with the relevant leadership in the treatment program. After treatment programs have agreed to participate, we anticipate that we will gather a verbal informed consent form from all of the participants for interviews and surveys. We will provide information about study goals and risks using a key information sheet for all web-based survey/interview administrations.

*Protection Against Study Risks for Interview Participants*

The risks to the participants in the study are minimal. They are limited to breach of confidentiality related to participation. The confidentiality of the participants will be protected in several ways. All participants will receive an identifying number for coding and analyzing data. Informed consent documents and identifying code numbers will be kept in a separate file from completed data questionnaires. REDCap, which is a secure platform managed by the NYU Langone Clinical and Translational Science Institute, will be used to collect the demographic data. Only the NYU research team will have access to the REDCap platform, which has detailed user access settings. Participants will only receive a Record ID in REDCap; their names will not be recorded anywhere. Data will be reported in a group format only; if any need should arise to report individual data, it will be done using a participant number or alias name. Any other potentially identifying information will also be removed (e.g., specific information about unusual characteristics or events that may be known in the community). All data will be kept in a locked file and will be accessible only to the research team. Participants’ names and identifying characteristics will not be used outside NYU.

***Vulnerable Subjects, if relevant to the study***

The only possible involvement of special vulnerable populations in our study would be pregnant women identified as individuals with SUD. However, as pre-existing administrative data will be used and data protection protocols enacted, the risk is minimized for all included individuals.

### Known Potential Benefits

Insights gained about successful training and protocols for THD as well as instituting data reporting to guide quality improvement from the proposed study will be directly relevant and beneficial to the population under study as they will improve the quality of treatment for OTPs.

Additionally, these findings will be more broadly relevant on a systems-level as they can inform best practices (with both health-outcome and cost-effectiveness data) in Medicaid. The risks to all study participants are reasonable in relation to the anticipated benefits to the participants and others. Procedures have been developed and will be in place throughout the proposed study to minimize these risks to the greatest degree possible. These procedures have been successful in preventing risk in past studies of similar populations.

The benefits of the proposed study are considerable. Within the context of the unprecedented pandemic around the world, research identifying the most effective clinical practices could help mitigate the severe morbidity and mortality that has ensued. The findings from this study would be directly relevant to the population under study, as well as similar populations across the country, and also at the systems level by informing best practices for OTPs in regards to THD. The information gained from this research will contribute to the scientific literature on SUD treatment programs during times of a pandemic. The knowledge gained will also contribute to the future policymaking in New York State and other states as OASAS remains committed to increasing THD flexibility in the system and will apply for federal waivers to extend the regulatory relaxation for THD indefinitely.

This study examines critical research questions by leveraging unique partnerships and methods.

This project innovations brings together an academic-government partnership to apply large administrative data to inform an intervention. We have long collaborated on examining the effects of system reform efforts. OASAS provides access to its data as well as an understanding of important policy considerations. The academic team brings the scientific framework, tools, and sophisticated statistical analyses for studying systems of care. These efforts inform the OASAS oversight function, including the use of data to inform system interventions.

We integrate multiple data sources to examine client and OTP organizational level factors associated with THD. We will join data from Medicaid and a treatment registry maintained by OASAS. The registry contains client-level socio-demographic (e.g., housing), other patient-level clinical factors (e.g., criminal justice involvement, injection drug use), and THD data that will supplement the Medicaid claims. We will also add data from the New York State’s mortality registry to examine differences in deaths across clinics. We will use qualitative interview data to explain and explore quantitative findings.

It addresses disparities in care for Black/African American and Latinx PWOUD. The intervention will specifically address subjective assessments (often racially biased) that lead to disparities in THD. The intervention will include dashboards that use State administrative data to highlight differences in THD by race, ethnicity, and gender.

We are addressing a critical need to understand the long-term implications of systemic changes emerging from the PHE—particularly for Black/African American and Latinx individuals with OUD. The THD regulatory changes present a unique circumstance under which a fragmented and fragile system of care must adapt to unprecedented changes. The implications for millions of individuals are enormous. Notably, we go beyond identifying racial/ethnic disparities in outcomes; we will identify structural and interpersonal reasons for disparities and examine whether our intervention improves them.

We are using mixed methods at a large—whole state—level to analyze systemic changes that have significant implications for policy at the local and national level.^[[143]](#endnote-143)^, ^[[144]](#endnote-144)^ By combining findings from analyses of large administrative data with lessons gleaned from qualitative interviews of successful and struggling clinics, we will build scientifically supported knowledge to inform policy at a larger systemic level.

# Objectives and Purpose

Regulatory changes made during the COVID-19 public health emergency (PHE) that relaxed criteria for take- home dosing (THD) of methadone offer an opportunity to improve retention in care with a lifesaving treatment. Methadone is a highly effective medication for treating opioid use disorders (OUD) that is provided in opioid treatment programs (OTPs). Yet, longstanding regulatory restrictions limit the availability of methadone as well as create demands that heavily burden clients by requiring frequent visits to clinics. The rationale for these regulations is to safeguard against diversion and overdoses from methadone. Yet, the history and application of methadone regulations stem from stigmatized and racist notions of people with OUD. Most OTPs are located within communities with predominantly Black/African American or Latinx populations. Consequently, Black/African American and Latinx individuals have greater access to methadone than other, less restricted, medications for OUD. Within OTPs, Black/African American and Latinx individuals are less likely to receive adequate dosing levels of methadone and have lower retention than non-Hispanic White clients. More flexible THD may help address disparities in care.

Currently, there is a national debate about balancing safety concerns over more flexible THD against the benefits of client retention and quality of life. Studies have not found increased overdoses under more flexible THD rules during the PHE; however, longer term studies are needed to understand best THD practices and outcomes. In New York, the Office of Addiction Services and Supports (OASAS) vigorously encouraged OTPs during the PHE to increase THD flexibility by highlighting clinical benefits, clarifying regulatory rules, monitoring OTP THD practices, and offering a new Medicaid payment to incentivize THD. The New York OTP system is large, serving >38,000 individuals each year. The state provides robust support to OTPs, with 83% of clients covered by Medicaid. New York data from the first year of the PHE show large variability across OTPs in the application of the more flexible THD criteria. Low offering of THD in many OTPs suggests a need for new data- driven interventions to encourage changes in engrained clinical workflows and long-standing stigmatizing beliefs about OUD clients. OTP leadership and staff express concern about misapplying regulatory flexibility, of iatrogenic effects of greater THD, and about legal liability from overdoses or diversion. Finally, financial concerns mount for organizations that have long based their business models on billing for frequent in-person medication dispensing. Buoyed by lack of evidence of adverse consequences from greater THD during the PHE, OASAS remains committed to maintaining increased THD flexibility in the system and will apply for federal waivers to extend the regulatory relaxation for THD indefinitely.

We propose a two-part project to develop then test a multidimensional OTP intervention to address clinical decision making, regulatory confusion, legal liability concerns, capacity for clinical practice change, and financial barriers to THD. The intervention will include OTP THD specific dashboards drawn from multiple State databases. The approach will be informed by the Health Equity Implementation Framework. In year 1, we will employ an explanatory sequential mixed method design to combine analysis of large state administrative databases—Medicaid, treatment registry, THD reporting—with qualitative interviews to refine the intervention. In years 2-5, we will conduct a stepped-wedge trial with 36 OTPs (~10,800 Medicaid clients/yr) randomized to 6 cohorts of a six-month long clinic-level intervention over three years. The trial will test the effects of the intervention on 1) THD; 2) retention in care; and 3) adverse healthcare events. We will specifically examine the effects of the intervention for Black/African American and Latinx clients.

## Primary Objective

**Year 1: Hone the Intervention**

Aim 1. Analyze administrative data to identify factors associated with OTP variation in THD practices and categorize clinics by THD flexibility.

Aim 2. Conduct qualitative interviews with leadership and staff of 10 OTPs (5 high and 5 low THD flexibility) on clinical and organizational factors affecting take-home dosing decisions.

Aim 3. Complete development of the multidimensional OTP intervention.

**Years 2-5: Stepped-Wedge Trial**

Aim 4. Test the effects of the intervention on THD, retention in care, and adverse events. Hypotheses 1-3: The intervention will increase H1) rates of 7-day, 14-day, and 28-day THD, H2) retention in care, yet H3) not change the rate of adverse events (e.g., emergency department visits, hospitalizations, all-cause mortality).

Aim 5. Conduct qualitative interviews with OTP leadership, staff, and clients on attitudes, experiences and behaviors related to the intervention.

Aim 6. Using mixed methods, explore variation in THD associated with race and ethnicity.

# Study Design and Endpoints

## Description of Study Design

This two-part proposal will develop, implement, and test protocols and tools for facilitating adoption of greater THD flexibility in OTPs. It will evaluate the impact of the intervention on acute healthcare events (i.e., emergency department visits and hospitalizations) as well as all-cause mortality. *We choose all- cause mortality for measurement concerns explained below.* Year 1 of the project will involve an explanatory sequential mixed methods design, starting with extensive analyses of state administrative data (Medicaid, treatment registry, OTP THD reporting) to examine patterns of THD as well as individual and organizational level factors associated with greater flexibility of THD. We will then interview staff at OTPs to obtain qualitative data on factors affecting THD. Based on the results, we will hone the OTP intervention described below. In years 2-5 of the project, we will employ a stepped-wedge randomized controlled trial to test the effectiveness of the intervention among 36 OTPs that are enrolled in six cohorts at staggered enrollment time points. The trial will employ an embedded mixed methods design to study the intervention impact as well as gain important tacit knowledge that will inform future dissemination.^[[145]](#endnote-145)^

## Study Endpoints

### Primary Study Endpoints

Milestones will include a) findings on patient and organizational factors associated with THD; b) findings on association with OTP THD practices and outcomes (retention in care, emergency department visits, hospitalizations, mortality); c) findings from qualitative interviews on organizational considerations for THD; and d) development of materials for the OTP intervention to improve THD. See milestone addendum for further details.

# Study Enrollment and Withdrawal

Opioid treatment programs (OTPs) will be recruited to participate in this stepped wedge trial by State partners via email invitations, announcements via State listservs, and announcements at relevant meetings to contact the Project Manager or the Principal Investigators. A total of 36 programs will be recruited at the beginning of the trial (Year 2). The intervention is delivered to the leadership and staff members at the clinic-level. Zoom meetings will be conducted with directors of programs to confirm their enrollment and to explain the timeline and expectations. The Project Manager will monitor enrollment and if enough clinics do not enroll, additional enrollment techniques will be discussed with the study team and implemented. In order to retain clinics in the trial, the study Project Manager and the Facilitators will stay in regular contact with the clinic program director to ensure that any concerns are addressed and to answer any questions. If a clinic is considering withdrawing from the study, the Project Manager will schedule an in-person/online meeting to further discuss and try to address reasons for desired withdrawal. We expect that offering the intervention to clinics will be attractive because the intervention will offer training and external facilitation free of charge, which the study team has found in their experience in providing Statewide technical assistance is very welcome during the period of state reforms and COVID-19 Public Health Emergency; therefore, do not anticipate problems with enrollment. Any issues with clinic enrollment and retention that cannot be resolved by the Project Manager will be discussed with the Principal Investigators for resolution. In addition, recruitment and retention will be discussed during regular weekly study team meetings.

## Inclusion Criteria

The study aims to address the adult opioid treatment program (OTP) system that serves more than 45,000 adults in New York each year. Based on our findings from analyses of administrative data in year 1, we will select 10 clinics for qualitative interviews with staff members to explore further clinic level factors associated with THD practices as well as technical assistance needs to implement more flexible THD. In years 2-5, we will employ a stepped-wedged randomized control trial with 36 OTPs which will be randomized into 6 cohorts of six-month long interventions over three years.

Clinic staff inclusion will include anyone who works at the 10 clinics that the OASAS client data system generates from the quantitative analysis in year one. In years 2-5, clinics chosen by the OASAS client data system will be placed into six cohorts. Only staff from these clinics will be eligible.

Patient inclusion anyone aged 18 or older who has been receiving take-home methadone for at least 30 days.

## Exclusion Criteria

There are no exclusion criteria related to sex/gender to increase the generalizability of the findings. We do not include children in this study because the treatment system that we are examining largely excludes adolescents and younger children.

## Vulnerable Subjects

No vulnerable subjects will participate in the qualitative interviews. We will not be interviewing staff of NYU Langone Health; thus, participants are not classified as a vulnerable population. Still, interviewees may feel obliged to complete the questionnaire and the interview if recommended by their organization. We will not be reporting back to leadership on participants therefore they will remain unaware of who agreed to participate and who did not.

## Strategies for Recruitment and Retention

Opioid treatment programs (OTPs) will be recruited to participate in this stepped wedge trial by State partners via email invitations, announcements via State listservs, and announcements at relevant meetings to contact the Project Manager or the Principal Investigators. A total of 36 programs will be recruited at the beginning of the trial (Year 2). The intervention is delivered to the leadership and staff members at the clinic-level. Zoom meetings will be conducted with directors of programs to confirm their enrollment and to explain the timeline and expectations. The Project Manager will monitor enrollment and if enough clinics do not enroll, additional enrollment techniques will be discussed with the study team and implemented. In order to retain clinics in the trial, the study Project Manager and the Facilitators will stay in regular contact with the clinic program director to ensure that any concerns are addressed and to answer any questions. If a clinic is considering withdrawing from the study, the Project Manager will schedule an in-person/online meeting to further discuss and try to address reasons for desired withdrawal. We expect that offering the intervention to clinics will be attractive because the intervention will offer training and external facilitation free of charge, which the study team has found in their experience in providing Statewide technical assistance is very welcome during the period of state reforms and COVID-19 Public Health Emergency; therefore, do not anticipate problems with enrollment. Any issues with clinic enrollment and retention that cannot be resolved by the Project Manager will be discussed with the Principal Investigators for resolution. In addition, recruitment and retention will be discussed during regular weekly study team meetings. Further information on interview/ survey recruitment will be discussed in section 6.

Patients will be recruited using the recruitment flyer. Anyone who fits the inclusion criteria will be able to express interest in interviewing. Clinics will post flyers throughout the clinic to allow interested eligible patients to participate.

## Duration of Study Participation

During year 1 of the study, we will draw potential sites from OTPs with high and low THD flexibility. We anticipate to recruit 10 OTPs (5 high and 5 low THD flexibility) on clinical and organizational factors affecting take-home dosing decisions based on our quantitative study during year 1. After the consent from the OTPs, we will recruit leadership (e.g., program director, clinical supervisor, etc.) and frontline treatment staff (e.g., addiction physicians, hospital nursing and social work leaders, hospitalists, administrative staff, etc.) for one-on-one interviews. Before the trial begins, clinics will be randomized into 6 cohorts of six-months long interventions over three years. Using large administrative data, we will be able to track clients within each OTP over four years, including a pre-intervention six-month baseline period. Six clinics in each cohort will receive the intervention for six months. In years 2-4, we plan to conduct a post-intervention survey with providers/staff and interviews with providers and patients. The qualitative research will include data from approximately 174 addiction treatment staff and 144 patients being interviewed about take-home dosing practice changes after the intervention has been implemented. Recruitment will end will end when either 174 staff and 144 patients are interviewed or saturation is reached.

## Participant Withdrawal or Termination

Participants are free to withdraw from participation in the study at any time upon request.

# Study Procedures and Schedule

## Study Procedures/Evaluations

### Year 1

We will develop an explanatory sequential mixed methods design^[[146]](#endnote-146)^, ^[[147]](#endnote-147)^ that combines analyses of administrative data with qualitative data collection to examine OTP organizational factors associated THD. We will combine data from four sources (described further below). An OASAS registry of OTP treatment episodes—the Client Data System (CDS)—will provide socio-demographic and clinical information on clients. An OASAS THD monitoring database will provide clinic aggregate data on THD practices. Medicaid data will provide healthcare, OTP services and billing data. New York Vital Statistics will provide data on all-cause mortality among OTP clients. Based on findings from analyses of these data, we will select 10 clinics for qualitative interviews with staff members to explore further clinic level factors associated with THD practices as well as technical assistance needs to implement more flexible THD.

Our analyses of the administrative data will begin with examination of distributions of variables and transformations where appropriate, examination of patterns of missingness, and application of formal imputation methods^[[148]](#endnote-148)^, ^[[149]](#endnote-149)^, ^[[150]](#endnote-150)^ if deemed essential to project aims. Formal statistical analyses will progress from the simple to the level of complexity necessary to examine factors associated with THD flexibility. We will begin with plotting observed THD patterns across OTPs, examine their bivariate relationship to client and provider characteristics, and with clinical outcomes (i.e., retention, emergency department visits, hospitalizations, mortality), then proceed to more complex mixed effect models to examine relative performance of OTPs on THD flexibility while adjusting for relevant client characteristics (e.g., time in treatment, housing status).^[[151]](#endnote-151)^, ^[[152]](#endnote-152)^, ^[[153]](#endnote-153)^, ^[[154]](#endnote-154)^ Ten top and bottom OTPs will be identified based on client case-mix adjusted models, then approached for qualitative interviewing. The analyses will also inform on typical billing patterns and provide data to inform development of the intervention’s net revenue tool. Baseline measures of characteristics of clients and OTPs will be assessed at admission to the treatment episode. Time-varying measures of treatment and healthcare services will be assessed monthly. 6.1.1.1 outlines these measures in a table.

Using the Health Equity Implementation Framework as a guide (HEIF),^[[155]](#endnote-155)^, ^[[156]](#endnote-156)^ we will interview senior leadership, medical staff (i.e., Medical Director, Lead Nurse), and counselors at the selected OTPs (n = 30). Interview domains will cover organizational facilitators and barriers to THD, factors considered in determining client level of stability, and operational considerations (e.g., use of technology) in offering THD. Interviews will also inform development of the net revenue tool. Interviews will be recorded and transcribed then analyzed using directed content analysis.^[[157]](#endnote-157)^

Milestones will include a) findings on patient and organizational factors associated with THD; b) findings on association with OTP THD practices and outcomes (retention in care, emergency department visits, hospitalizations, mortality); c) findings from qualitative interviews on organizational considerations for THD; and d) development of materials for the OTP intervention to improve THD.

Year One analysis will be conducted in collaboration with all study investigators (Neighbors, Bao and Ramsay)

We will examine OTP staff and leadership attitudes, experiences, and behaviors related to implementing the OTP intervention. There will be three data sources: 1) implementation surveys, 2) semi- structured interviews, and 3) external facilitator notes, surveys, and checklists.^[[158]](#endnote-158)^, ^[[159]](#endnote-159)^, ^[[160]](#endnote-160)^ The Klein implementation survey^[[161]](#endnote-161)^, ^[[162]](#endnote-162)^ will be administered to OTP staff at 6-months post-intervention. Semi-structured interviews (n=72) will be conducted with at least one clinical and one member of OTP executive leadership at each OTP at the end of the six-month intervention phase. An interview guide will be created to reflect the dimensions of the HEIF, including patient-provider encounter factors from the Kilbourne framework as well as implementation factors from the i-PARIHS model.^[[163]](#endnote-163)^, ^[[164]](#endnote-164)^, ^[[165]](#endnote-165)^, ^[[166]](#endnote-166)^ In order to inform replication and dissemination of this intervention model, as well as contextual factors affecting sites’ ability to implement THD, the facilitator will complete logs after each interaction with a study site.^[[167]](#endnote-167)^

After individual clinics are recruited, leadership will reach out to clinics to identify staff members who may be interested and can provide important information for the research study. Staff members do not have to participate if they do not want to and their participation will never be discussed with leadership to avoid coercion. Staff members will be contacted via email along with a key information sheet for the survey and interviews.

All staff and client interviews (~60 minutes) will be conducted by master’s level interviewers with qualitative experience under the direction of Drs. D’Aunno and O’Grady. Therefore, Dr. O’Grady from the University of Connecticut will be involved in the consent process. Researchers will take field notes after interviews. Additional interviews will be conducted if our planned samples do not reach saturation.^[[168]](#endnote-168)^

Analyses will begin with examination of distributions of variables and transformations where appropriate, examination of patterns of missingness, and application of formal imputation methods ^[[169]](#endnote-169)^, ^[[170]](#endnote-170)^, ^[[171]](#endnote-171)^ if deemed essential to project aims. Formal statistical analysis will progress from the simple to the level of complexity necessary to test study hypotheses. We will begin with plotting observed outcomes, examine their bivariate relationship to client and provider characteristics, then proceed to more complex General Linear Mixed Models (GLMM) for stepped-wedge designs.^[[172]](#endnote-172)^

####
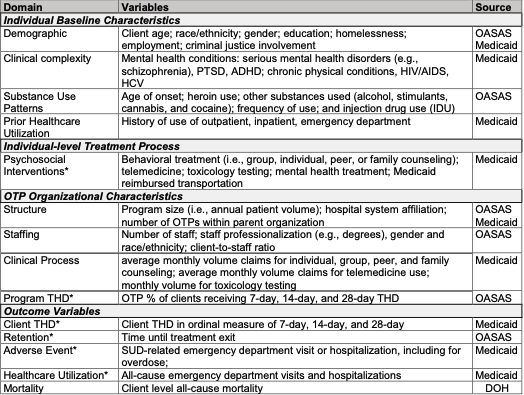
Measures

### Years 2-5

We propose an embedded mixed methods^[[173]](#endnote-173)^ study in which we will employ a stepped-wedged randomized controlled trial with 36 OTPs to test whether the intervention a) increases THD, b) increases retention in care, and c) has any effect on adverse events (i.e., emergency department visits, hospitalizations, mortality). OTPs will be randomized across 6 sequential steps between years 2 and 4. The examination of outcomes will benefit from access to rich sources of data described below. Qualitative data collection with staff and patients will be embedded within the trial to examine experiences with the new THD protocols and to inform quantitative findings. Based on analyses conducted during phase 1 of this project, we will recruit 36 OTP clinics from among the lower ranked OTPs based on client case-mix adjusted THD performance. Clinic recruitment will also be proportionate to the geographical distribution of OTPs across the State. In a current trial with similar approach (R61/33DA049252),^[[174]](#endnote-174)^ we have had strong success in recruiting and retaining 30 non-OTP outpatient clinics.

Before the trial begins, clinics will be randomized into 6 cohorts of six- month long interventions over three years.^[[175]](#endnote-175)^, ^[[176]](#endnote-176)^, ^[[177]](#endnote-177)^, ^[[178]](#endnote-178)^, ^[[179]](#endnote-179)^ Using large administrative data, we will be able to track clients within each OTP over four years, including a pre-intervention six-month baseline period. Six clinics in each cohort will receive the intervention for six months.

Client interviews (n=40) from participating OTPs will explore experiences related to THD, notably among Black/African American and Latinx clients. An interview guide will be created to reflect the dimensions of the HEIF. We will use maximum variation purposeful sampling by recruiting clients from participating OTPs in urban and rural locations as well as areas with variation in population characteristics. Further, we will recruit clients who are on a variety of THD schedules and ensure that we have representation based on gender, race, and ethnicity. OTPs will assist with recruiting clients by posting and handing out flyers with study information.

#### Intervention

The proximal goal will be to increase OTP uptake of flexible THD, which will increase retention in care without increasing adverse healthcare events or mortality. The intervention addresses
facilitators and barriers to THD practices as identified by existing studies as well as those found during the first phase of the current project. Guided by HEIF and research on organization
change,^[[180]](#endnote-180)^, ^[[181]](#endnote-181)^, ^[[182]](#endnote-182)^, ^[[183]](#endnote-183)^, ^[[184]](#endnote-184)^ the intervention will be designed to address the information gaps, training needs, and beliefs of individuals across the organizations. Senior leadership have purview over
financial and legal matters. Medical directors and nursing staff are responsible for dosing decisions consistent with best clinical practices. A designated external facilitator will work closely with each clinic using remote conferencing capabilities. These facilitators will be trained in quality improvement practices and data management as well as relevant clinical topics (e.g., THD best practices). They will be supervised in weekly intervention oversight meetings by study team members (Neighbors, Ramsey, Bao, D’Aunno, O’Grady, Lincourt). Facilitators will work closely with an identified member of clinic leadership, a clinical champion, and a small implementation team (e.g., 4-6 staff)

## Study Schedule

**Years 2-5 OTP THD Intervention**

| **Component** | **Who** | **Description** |
| --- | --- | --- |
| Data Dashboard | All OTP Staff | Performance feedback is an organizational intervention with robust scientific support. ^[[185]](#endnote-185)^, ^[[186]](#endnote-186)^, ^[[187]](#endnote-187)^ Drawing from analytical methods applied during phase 1 of the project, provide clinic-level reports that show relative performance compared to other OTPs within the State on THD, retention, and adverse healthcare events. Relative performance will be adjusted for clients’ demographic and clinical characteristics.^[[188]](#endnote-188)^, ^[[189]](#endnote-189)^, ^[[190]](#endnote-190)^, ^[[191]](#endnote-191)^ The data feedback will specifically detail THD performance by race, ethnicity, and gender. Reports would be available for all staff. |
| Legal/Regulatory | All OTP Staff | To address barriers related to uncertainty over legal and regulatory risk,^[[192]](#endnote-192)^^[[193]](#endnote-193)^, ^[[194]](#endnote-194)^, ^[[195]](#endnote-195)^, ^[[196]](#endnote-196)^, ^[[197]](#endnote-197)^ provide guidance on Federal and State requirements for THD as well as guidance on managing legal liability risk. Guidance would be available for all staff. |
| Financial Guidance | Senior Leadership | To address senior leadership concerns about fiscal viability, provide a net revenue tool along with training to allow clinics to model out the financial effects of using new THD bundled payment billing. The tool and guidance will walk through projections for number of clients with THD, revenue, and related costs. Leadership would be able to conduct ‘what-if’ analyses under varying demand and revenue assumptions.^[[198]](#endnote-198)^, ^[[199]](#endnote-199)^ |
| THD Best Practices | Clinical Staff | Drawing from extant research as well as from findings from qualitative studies conducted during phase 1, provide training on clinical best practices for THD. The training would address parameters for determining level of client stability, protocols for monitoring client status, and use of technology.^[[200]](#endnote-200)^, ^[[201]](#endnote-201)^, ^[[202]](#endnote-202)^ The training will also highlight any clinic THD variation by race, ethnicity, or gender. The training would specifically address concerns and best practices for managing risks of methadone overdose and/or diversion. Dr. Ramsey and Ms. Falkowski from our team will provide training to Medical Directors and nursing staff. |
| Process Change | Clinical and Administrative Staff | Training and support on process improvement strategies drawn from organizational change management. The OTP will be asked to designate a clinic implementation team led by local champion to review data and guide practice change projects.^[[203]](#endnote-203)^, ^[[204]](#endnote-204)^, ^[[205]](#endnote-205)^, ^[[206]](#endnote-206)^, ^[[207]](#endnote-207)^, ^[[208]](#endnote-208)^ The project facilitator will train and support the implementation team on rapid cycle change strategies drawn from management science and used in our current clinic-level trial. |

# Assessment of Safety

## Investigator Reporting

Federal regulations require timely reporting by investigators to their local IRB of unanticipated problems posing risks to subjects or others. The following describes the NYULMC IRB reporting requirements, though Investigators at participating sites are responsible for meeting the specific requirements of their IRB of record.

### Safety and Adverse Events

At each contact with the subject, the investigator must seek information on adverse events by specific questioning and, as appropriate, by examination. The only possible adverse events for this study are breach of confidentiality.

All adverse events occurring during the study period must be recorded.

## Notifying the IRB

**Report Promptly, but no later than 5 working days:**

Researchers are required to submit reports of the following problems promptly but no later than 5 working days from the time the investigator becomes aware of the event:

- ***Unanticipated problems including adverse events* that are unexpected and related**
  - *Unexpected****:*** *An event is “unexpected” when its specificity and severity are not accurately reflected in the protocol-related documents, such as the IRB-approved research protocol, any applicable investigator brochure, and the current IRB-approved informed consent document and other relevant sources of information, such as product labeling and package inserts.*
  - *Related to the research procedures****:*** *An event is related to the research procedures if in the opinion of the principal investigator or sponsor, the event was more likely than not to be caused by the research procedures.*
  - *Harmful: either caused harm to subjects or others, or placed them at increased risk*

**Other Reportable events:**

The following events also require prompt reporting to the IRB, though ***no later than 5 working days***:

- ***Complaint of a research subject*** when the complaint indicates unexpected risks or the complaint cannot be resolved by the research team.
- ***Breach of confidentiality***
- ***Incarceration of a participant*** when the research was not previously approved under Subpart C and the investigator believes it is in the best interest of the subject to remain on the study.
- ***New Information indicating a change to the risks or potential benefits*** of the research, in terms of severity or frequency (e.g. analysis indicates lower-than-expected response rate or a more severe or frequent side effect; other research finds arm of study has no therapeutic value; FDA labeling change or withdrawal from market).

## Study Oversight

It is the responsibility of the Principal Investigator, Dr. Charles Neighbors, to oversee the safety of the study at his/her site. This safety monitoring will include careful assessment and appropriate reporting of adverse events, as well as the implementation of a site data and safety-monitoring plan.

The Principal Investigator is a clinical psychologist who will be responsible for distinguishing between a Serious Adverse Event (SAE) and a non-serious adverse event (AE). All SAE and AE will be reported to the NYU IRB within 48 hours of its occurrence. Neighbors will prepare an annual report summarizing all SAE and AE to be submitted to NIDA and the IRB. Primary responsibility and decision making in regards to participants remaining in or being withdrawn from the study will lie with the principal investigator*.}*

# Statistical Methods

## Year 1 Analyses

Our analyses of the administrative data will begin with examination of distributions of variables and transformations where appropriate, examination of patterns of missingness, and application of formal imputation methods ^[[209]](#endnote-209)^, ^[[210]](#endnote-210)^, ^[[211]](#endnote-211)^ if deemed essential to project aims. Formal statistical analyses will progress from the simple to the level of complexity necessary to examine factors associated with THD flexibility. We will begin with plotting observed THD patterns across OTPs, examine their bivariate relationship to client and provider characteristics, and with clinical outcomes (i.e., retention, emergency department visits, hospitalizations, mortality), then proceed to more complex mixed effect models to examine relative performance of OTPs on THD flexibility while adjusting for relevant client characteristics (e.g., time in treatment, housing status).^[[212]](#endnote-212)^, ^[[213]](#endnote-213)^, ^[[214]](#endnote-214)^, ^[[215]](#endnote-215)^ Ten top and bottom OTPs will be identified based on client case-mix adjusted models, then approached for qualitative interviewing. The analyses will also inform on typical billing patterns and provide data to inform development of the intervention’s net revenue tool.

## Years 2-5 Analyses

### Analysis of the effect of the OTP Intervention on THD, retention in care and adverse events

Analyses will begin with examination of distributions of variables and transformations where appropriate, examination of patterns of missingness, and application of formal imputation methods ^[[216]](#endnote-216)^, ^[[217]](#endnote-217)^, ^[[218]](#endnote-218)^ if deemed essential to project aims. Formal statistical analysis will progress from the simple to the level of complexity necessary to test study hypotheses. We will begin with plotting observed outcomes, examine their bivariate relationship to client and provider characteristics, then proceed to more complex General Linear Mixed Models (GLMM) for stepped-wedge designs. ^[[219]](#endnote-219)^

We will employ GLMM models commonly used in the analysis of stepped-wedge trials. ^[[220]](#endnote-220)^, ^[[221]](#endnote-221)^ The models will take the form: 𝐹(𝜇 )=𝜇+𝛽 +𝛿𝑋 +𝛼 +𝜙 ,𝛼~𝑁(0,𝜏2),𝛾 ~𝑁(0,𝜏2) where 𝛽 is a fixed effect for time, 𝑋 is an indicator for intervention in clinic i at time j (coded 0 for the control condition, then 1 from when intervention begins in the clinic through end of study), and 𝛿 is the treatment effect. The models have random effects for clustering at the clinic and individual patient levels, which are assumed to be normally distributed with mean zero and variances 𝜏2 and 𝜏2 , respectively. We will estimate the 𝛼𝜙 effect of intervention while controlling for secular trends and adjusting for clustering within clinic (𝛼𝑖) and individual (𝜙𝑖𝑘), using mixed effects modeling with binary distribution and logit link. Models will be fit using the *glmmTMB* package using R software.^[[222]](#endnote-222)^, ^[[223]](#endnote-223)^ In the case of H1, the primary outcome will be weekly or greater THD, and the fixed-effects coefficients for the intervention effect, exponentiated, will indicate how the intervention increases the odds of weekly or greater THD. Similar mixed models will consider other THD frequencies as outcomes (i.e., 14-day and 28-day). In the case of H3, the same mixed logistic regression model will be used with any adverse events as the outcome, but a one-side test of non-inferiority (i.e., adverse events are no worse under intervention than under the control condition) will be used.^[[224]](#endnote-224)^ We will use a non-inferiority margin of OR≈1.13, which corresponds to an increase in the prevalence of adverse events from 0.20 under the control condition to 0.22 under intervention. In other words, we will test whether the intervention effect is less than OR≈1.13 in a one-sided test. This is equivalent to obtaining a 95% confidence interval for the intervention odds ratio where the upper limit is less than 1.13.

For the retention outcome (H2), a multilevel discrete-time survival model will be used.^[[225]](#endnote-225)^, ^[[226]](#endnote-226)^, ^[[227]](#endnote-227)^An expanded person-period dataset will be constructed.^[[228]](#endnote-228)^ The outcome will have two possible states in any time period: 0 = retained in treatment; 1 = treatment exit. The multilevel analysis approach can accommodate recurring events for the same individual, with individuals leaving the set of patients “at risk” for dropout until treatment is initiated again. Analysis of the person-period dataset will use a mixed-effects regression model with a complementary log-log link function and random effects for patient and clinic. With the complementary log-log link, the coefficients can be interpreted as the relative effect on the hazard of event occurrence. Discrete time intervals in which an individual may be retained or not retained will be one month in length, and a set of dummy variables will capture differences in risk of dropout across these intervals. Time will be relative to the initiation of treatment, which may have been many months before the start of the study for some patients. In a sensitivity analysis, we will restrict the retention outcome to patients who have been in treatment for less than 12 months at the start of the study. The discrete-time multilevel model accommodates both time-invariant patient characteristics as well as time-varying patient and clinic characteristics as explanatory variables. Our focus will be on the time-varying intervention condition variable at the clinic-level, adjusting for patient characteristics such as demographics and prior treatment experience. Intervention effects will be visualized by plotting fitted hazard of dropout against month since treatment initiation separately for control and intervention observations. The multilevel discrete-time survival model can be used for other time to event outcomes such as time to all- cause mortality.

#### Further Exploratory Analyses

Should we find significant treatment effects of the OTP intervention, we will conduct a series of analyses to develop further evidence that bolsters the inference that the effect is due to the treatment.^[[229]](#endnote-229)^, ^[[230]](#endnote-230)^, ^[[231]](#endnote-231)^ We borrow from methods used in mechanism of treatment research that test whether postulated factors associated with the treatment are also related to the outcome.^[[232]](#endnote-232)^, ^[[233]](#endnote-233)^, ^[[234]](#endnote-234)^ We will test the association between putative mediators and outcomes. For example, the putative mechanism for the OTP intervention is that increased THD will improve retention in care. We will examine the association between THD and retention in care as mediators of the intervention effect. We will also see whether the intervention effect is moderated by characteristics of the population or regional variations. For example, we will also examine variation in intervention impact by region and gender. Any significant moderator effects (e.g., differences in effect size by gender) will suggest that there may be other factors that may account for some of the observed intervention effect. We will also explore variation in effect of the intervention by providers to see if there are moderating effects that suggest variation in treatment implementation. Finally, we will test whether there are lagged treatment effects by introducing interaction terms between treatment and time since intervention start by cohort.^[[235]](#endnote-235)^, ^[[236]](#endnote-236)^ These findings will inform our qualitative study methods. We view these analyses as exploratory since we do not have strong hypotheses regarding the nature of these associations, yet we will use these findings to refine further our interpretation of results and presentation of study findings.

### Analysis of qualitative interviews with OTP leadership, staff, and clients

A concurrent triangulation mixed-methods design will be used such that quantitative and qualitative data collection will occur concurrently and results will be integrated after analysis of each.^[[237]](#endnote-237)^ The semi-structured interviews will be transcribed and analyzed with Atlas.ti to identify key facilitators, barriers, and experiences according to the HEIF constructs using directed content analysis^[[238]](#endnote-238)^ integrated with a framework method.^[[239]](#endnote-239)^ Researchers will make field notes immediately after interviews. We will use the first few interviews to develop the coding scheme, and additional transcripts will be compared to previously coded transcripts to ensure the consistent assignment of codes. As emerging concepts are identified, we will adapt the existing coding structure. Using principles of grounded theory, data collection and analysis will be iterative, with the interview adapted to reflect emerging themes. The research team will review the structure of codes to ensure that it is logical and comprehensive. After the team has reviewed the coding structure and all interviews have been reviewed in depth by two researchers, trained project staff will independently code all transcripts using the final coding scheme. Twenty percent of the transcripts will be double coded to assess inter-coder agreement. Any differences in coding will be discussed and resolved after discussion with the investigators. We also will create an analysis audit trail to document all analytic decisions. Targeted analyses will examine the consistency of the data within sites and identify those themes which appear to discriminate between high and low-performing THD OTPs. When coding is complete, the team will meet to review summaries of the qualitative results and refine hypotheses about the contextual factors and strategies that lead to better outcomes and those that might be barriers to success. For the quantitative results, staff responses will be aggregated across each survey time point and group means and standard deviations will be calculated. Multilevel modeling will be used to examine change over time in survey scores given the nested structure of the data (staff nested within clinics).^[[240]](#endnote-240)^

### Analysis of variation in THD associated with race/ethnicity

The quantitative components of Aim 6 (H4-H6) will be examined by including additional terms in the mixed- effects logistic regression models and in the multilevel discrete-time survival model to incorporate interactions between the intervention effect and patient race/ethnicity. If significant interaction effects are detected, simple main effects of intervention will be estimated for each racial/ethnic group to understand how the intervention effect may vary and to identify any inequities in intervention impact.

## Statistical Power

To estimate power for select outcomes, we used a combination of PASS 2022 and Monte Carlo simulation.^[[241]](#endnote-241)^ Using administrative data to estimate sample sizes and baseline rates (μ), we compute detectable differences for 80% power, α = 0.01, ICC=0.05, and SW-RCT with 6 crossover points, and 6 clinics randomized to start at each crossover point. For 7-day or greater THD (i.e., picking up doses every 7 days or less often), we assume the currently prevalence is 0.45. With 36 clinics and approximately 350 clients observed in each clinic in each six-month period (n≈88,200 total observations), power is 93% to detect a small increase (OR=1.1) in the odds of 7-day or greater THD. This corresponds to an increase from a prevalence of 0.45 under the control condition to .47 after intervention. Even when considering racial/ethnic patient subgroups making up just 10% of clinic clients (n≈35 per site and period), power is 86% to detect a modest increase in the odds of 7-day or greater THD (OR=1.3; an increase from 0.45 to 0.52). To estimate power for non-inferiority on the adverse events outcome, we simulated five thousand datasets from a mixed-effects

logistic regression model with the planned stepped-wedge design with no intervention effect. For each of these simulated datasets, the 95% confidence interval for the intervention effect was estimated. Across the five thousand datasets, the upper limit of the odds ratio was less than 1.13 in 4555 datasets, indicating approximately 91% power for the non-inferiority test. Because power is sufficient to detect small intervention effects for the binary outcomes of 7-day or greater THD and adverse events, it is also sufficient to detect small intervention effects on retention in care.

# Ethics/Protection of Human Subjects

## Ethical Standard

The Principal Investigator will ensure that this study is conducted in full conformity with Regulations for the Protection of Human Subjects of Research codified in 45 CFR Part 46*.*

## Institutional Review Board

The protocol, informed consent forms, recruitment materials, and all participant materials will be submitted to the IRB for review and approval. Approval of both the protocol and consent documents must be obtained before any participant is enrolled. Any amendment to the protocol will require review and approval by the IRB before the changes are implemented to the study. All changes to the consent form will be IRB approved; a determination will be made regarding whether previously consented participants need to be re-consented.

A study closure report will be submitted to the IRB after all research activities have been completed.

## Informed Consent Process

In obtaining informed consent, the study team will comply with applicable regulatory requirements and adhere to 45 CFR Part 46. All consent materials (described below) will receive written approval from the IRB prior to being utilized.

### Consent/Assent and Other Informational Documents Provided to Participants

The consent materials attached to this protocol, which adhere to all required regulatory elements, are as follows:

- Part 1 (Year 1- Aim: Conduct qualitative interviews with leadership and staff of 10 OTPs (5 high and 5 low THD flexibility) on clinical and organizational factors affecting take-home dosing decisions.
  - Provider Interview Guide
  - Provider Key Study Information Form
  - Provider Staff Recruitment Script
  - Provider Verbal Consent
- Part 2 (Years 2-5- Aim: Conduct qualitative interviews with OTP leadership, staff, and clients on attitudes, experiences and behaviors related to the intervention.)
  - Patient Interview Guide
  - Patient Key Study Information Form
  - Patient Verbal Consent
  - Patient Recruitment Flyer
  - Provider Interview Guide
  - Provider Key Study Information Form- Interview Portion
  - Provider Key Study Information Form- Survey Portion
  - Provider Recruitment Email
  - Provider Verbal Consent
  - Provider Staff Measures Quantitative Survey

### Waivers

We are seeking the following waivers:

- Waiver of authorization for the collection of administrative data
- Waiver of consent documentation for provider and patient participation in surveys/interview.

## Consent Procedures and Documentation

Prior to scheduling interviews, we will send key information sheets and verbal consent script to any potential participants. Potential participants can, then, agree to schedule a time to speak and each staff member will be asked to provide verbal consent if they are still willing to participate in the interview. At the beginning of each interview, the interviewer will use the verbal consent script to: explain the purpose, risks, and benefits of the study, address any questions or concerns the participant may have, and obtain the participant’s verbal informed consent to participate in the interview and be audio-recorded. We prefer a verbal consent process as this poses less burden to the participants. It will also allow participants to ask questions before they agree to participate in the study. Because we will be using a verbal consent process, we are requesting a documentation of consent waiver (attached).

## Participant and Data Confidentiality

Every effort will be made to ensure participant confidentiality. Leadership at participating clinics will not be informed about any information regarding participation in the study. All of the personnel and staff on the study team have been certified in NYU’s Human Research Social/Behavioral Research Course through the CITI program. The study team recognizes that the protection of human subjects relies on policies that secure data with personally identifiable information. Each of the investigators has adopted, and will strictly follow, such policies for all data used in the study. Any time data is reported, it will be aggregated with that of other respondents (e.g., to discuss identified themes or key concepts) and never reported with personally identifiable information. No information will be reported that could make it possible for anyone to identify participants in any presentations or written reports about this study. Any identifiable personal information that participants may reveal about themselves during the interviews will be removed from the transcripts. If a direct quote from an interview is utilized in a report or publication (e.g., to exemplify a concept that arose in multiple interviews), it will only be cited using a pseudonym or participant ID number. All information will be stored in our secure, encrypted NYU drives. Personal identifying information will never be shared with anyone outside of the research team.

Information about study subjects will be kept confidential and managed according to the requirements of the Health Insurance Portability and Accountability Act of 1996 (HIPAA). Those regulations require a signed subject authorization informing the subject of the following:

- What protected health information (PHI) will be collected from subjects in this study
- Who will have access to that information and why?
- Who will use or disclose that information?
- The rights of a research subject to revoke their authorization for use of their PHI.

In the event that a subject revokes authorization to collect or use PHI, the investigator, by regulation, retains the ability to use all information collected prior to the revocation of subject authorization. For subjects that have revoked authorization to collect or use PHI, attempts should be made to obtain permission to collect at least vital status (i.e. that the subject is alive) at the end of their scheduled study period.

# Data Handling and Record Keeping

## Data Collection, Confidentiality and Management Responsibilities

Following each interview, digital audio files of the interviews will be transcribed by Research Assistants at NYU Langone. After transcripts are checked for accuracy, audio files will be destroyed and only transcripts will be stored on a secure MCIT managed network shared drive. Contact information will be stored on a MCIT-managed network drive.

We recognize the importance of providing a plan to ensure scientific integrity and safeguard the well-being of study participants. Because the current study poses little risk to the participants, does not involve blinding of providers or participants, and does not involve a drug trial, we believe that the study qualifies for monitoring at the level of the PI. During the informed consent process, participating interviewees and their office staff will be informed that any breach of confidentiality needs to be reported to the Project Director. If any participant appears to be at risk in any way as a result of participation in the study, the IRB at NYU Langone will be notified as soon as the immediate needs of the individual have been addressed.

Subject confidentiality is held in strict trust by the research team. It is the responsibility of the Principal Investigator to oversee the study at his site.

Every effort will be made to ensure participant confidentiality as prescribed by the NYU Grossman School of Medicine Institutional Review Board and HIPAA standards. All of the personnel and staff on the study team have been certified in human subjects education from the CITI program. The study team recognizes that the protection of human subjects relies on policies that secure the data with personally identifiable information and that limit the release of analysis and results to aggregations and estimated parameters that cannot reveal information about an individual. Each of the investigators has adopted, and will strictly follow, such policies for all data used in the study.

## Study Records Retention

All research data will be stored on secure, password-protected computers managed by NYU Langone MCIT and will be properly backed up and retained by NYU Langone Health for the period required by the NYU Langone Health’s Workforce Member IT Policy. Contact information will not be published by any means. Only trained research staff will have access to the data. Study documents will be retained for 3 years after the completion of the project in accordance with the NYU Langone policy on research data relating to projects subject to the review of the IRB.

PI will maintain all audio recordings from interviews in electronic form. Research data continuously stored in these locations will be properly backed up and retained by NYU Langone Health for the period required by the NYU Langone Health’s Workforce Member IT Policy. Contact information will not be published by any means. Only research staff will have access to the data. Study documents will be retained for 3 years after the completion of the project in accordance with the NYU Langone policy on research data relating to projects subject to the review of the IRB.

## Protocol Deviations

- Any unanticipated problems will be promptly reported to the IRB (including intentional and accidental/unintentional deviations from the IRB approved protocol) for any of the following situations:
  - *one or more participants were placed at increased risk of harm*
  - *the event has the potential to occur again*
  - *the deviation was necessary to protect a subject from immediate harm*
- ***Breach of confidentiality***
- ***Incarceration of a participant*** when the research was not previously approved under Subpart C and the investigator believes it is in the best interest of the subject to remain on the study.
- ***New Information indicating a change to the risks or potential benefits*** of the research, in terms of severity or frequency (e.g. analysis indicates lower-than-expected response rate or a more severe or frequent side effect; other research finds arm of study has no therapeutic value; FDA labeling change or withdrawal from market).

### Reporting Process

The reportable events noted above will be reported to the IRB using a Reportable New Information submission and will include a description of the event with information regarding its fulfillment of the above criteria, follow-up/resolution, and need for revision to consent form and/or other study documentation. Copies of each report and documentation of IRB notification and receipt will be kept in the Clinical Investigator’s study file.

## Publication and Data Sharing Policy

This study will comply with the NIH Public Access Policy, which ensures that the public has access to the published results of NIH funded research. It requires scientists to submit final peer-reviewed journal manuscripts that arise from NIH funds to the digital archive PubMed Central upon acceptance for publication. The principal investigator at New York University Grossman School of Medicine (Charles Neighbors) will be responsible for registering the clinical trial and reporting the summary results in ClinicalTrials.gov as outlined in the NIH Policy on the Dissemination of NIH-Funded Clinical Trial Information, in order to support the NIH’s mission of broad and responsible dissemination of research. Record information will be entered within the timelines specified in the policy, i.e. initial registration of the trial no later than 21-day post-enrollment of the first participant and summary results posted no later than 12 months post-primary completion date. Additionally, informed consent documents for participants will include a statement that study will be posted on ClinicalTrials.gov. NYU Langone Sponsored Programs Administration team sets forth internal institutional guidelines to ensure compliance with NIH policy and applicable federal regulations and is available to support investigators with registering or entering summary results into the ClinicalTrials.gov database.

The data generated in this grant will be presented at national or international conferences and published in a timely fashion. All final peer-reviewed manuscripts that arise from this proposal will be submitted to the digital archive PubMed Central.

Project data will be made available to the Data Ecosystem in a manner that is consistent with Data Use Agreements with the data owners. The data owners are only permitted to share these data at the person level as long as it supports the administration of the Medicaid program. Generally, we will be able to submit reports that present statistical data at an aggregate level in a form permissible and approved by the owner. The data for the quantitative components of this study will be culled from the administrative data files of the New York State Office of Alcoholism and Substance Abuse Services (OASAS) and the New York State Department of Health (DOH). Due to the nature of the data security agreements between OASAS/DOH and NYU HEAL, we will not be able to make person-level data available for sharing. Subject to approval from the Department of Health and OASAS, we can provide tables of data summaries categorized by demographic, geographic, and provider characteristics.

We will be able to provide summary reports from qualitative data since the number of participants is small and the risk of being identification is high. The proposed qualitative research will include data from approximately 174 addiction treatment staff and 144 patients being interviewed about take-home dosing practice changes after the intervention has been implemented. The final dataset will include self-reported demographic and experience data. Even though the final dataset will be stripped of identifiers prior to release for sharing, there remains the possibility of deductive disclosure of participants with easily identifiable characteristics which could possibly affect participants’ employability. This is especially the case because the data will be collected from a small number of staff at each participating clinic and it may be known by the provider community which clinics in each identified region are participating in the study.

Thus, we will make the data and associated documentation available to users who request it in writing only under a data-sharing agreement approved by study co-investigators that provides for: (1) a commitment to using the data only for research purposes and not to identify any individual participant; (2) a commitment to securing the data using appropriate computer technology; and (3) a commitment to destroying or returning the data after analyses are completed. This data sharing plan will be made clear in consent documents for participants.

# Study Finances

## Funding Source

This study is funded by NIDA grant opportunity HEAL Initiative: HEAL Data2Action Innovation Projects (ID: RFA-DA-22-051)

## Costs to the Participant

There are no costs to subjects for participating in this study

## Participant Reimbursements or Payments

Participants in the Provider Part 1 interviews will be compensated $40 for their participation. Clients will receive a $30 incentive to participate.^[[242]](#endnote-242)^

# Study Administration

## Study Leadership

The Study PI’s, Dr. Charles Neighbors, Dr. Kelly Ramsay and Dr. Yuhao Bao govern the conduct of the study. It is the responsibility of the Principal Investigators to oversee the safety of the study at the study site. This safety monitoring will include careful assessment and appropriate reporting of adverse events, as well as the implementation of a site data and safety-monitoring plan. This award is subject to the Data and Safety Monitoring Plan (DSMP) submitted and approved by NIDA, via email on June 23, 2020.

## Data and Safety Monitoring Plan (DSMP)

The Principal Investigators are a clinical psychologist (Neighbors), health economist and behavioral health services researcher (Bao), and Chief of Addiction Medicine (Ramsay) who will be responsible for distinguishing between a Serious Adverse Event (SAE) and a non-serious adverse event (AE). All SAE and AE will be reported to the NYU IRB and to the NIDA project officer within 48 hours of its occurrence. Additionally, one of the PIs (Neighbors) will prepare an annual report summarizing all SAE and AE to be submitted to the NIDA project officer and the IRB. All participants in the study will be participating in treatment in the outpatient program and will receive regular monitoring and services as part of that program. If there is a significant increase in drug use since the baseline assessment; or other indicators through any interaction with the research staff that the patient is deteriorating, the participant will be immediately withdrawn from the study and referred to immediate and appropriate treatment. Primary responsibility and decision making in regards to participants remaining in or being withdrawn from the study will lie with the principal investigators in collaboration with the patient’s treating clinical staff at the outpatient clinic where they are receiving treatment.

The aims of the project are to help clinic staff improve quality of their operations, and there are no safety issues associated with participation.

## Rationale for Multiple Principal Investigators and Site Responsibilities

Given the complexity of the grant as well as its relevance to the State, we believe that a multiple PI leadership model bringing together an academic-state partnership would bring the necessary breadth and scope in leadership capabilities for the project as well as aid in dissemination of the findings. Each Principal Investigator, Dr. Neighbors, Dr. Ramsey and Dr. Bao, brings unique expertise, diverse backgrounds, and complimentary experiences and resources that are complementary and essential to the success of the proposed study. Dr. Neighbors is an Associate Professor and the Director of the Health Evaluation and Analytics Lab (HEAL) at the NYU Grossman School of Medicine (NYUGSOM). As a health services researcher, Dr. Neighbors has worked for more than 15 years with OASAS on research related to improving the addictions treatment system, and has expertise and experience conducting complex analyses of the State’s administrative datasets. Dr. Ramsey is the Chief of Addiction Medicine for the New York State (NYS) Office of Addiction Services and Supports (OASAS). She brings experience and authority as the State’s medical policy voice for OTPs, as well as her experience as a medical director in OTPs. Dr. Bao is a health economist and Associate Professor of Population Health Sciences (with a secondary appointment in Psychiatry) at Weill Cornell Medical College. She has a unique research program aimed at aligning incentives (especially provider payment models) with evidence-based, integrated care for people with mental health and/or substance use conditions.

Dr. Neighbors will lead project management of data analyses and the stepped-wedge trial. The trial will draw from protocols of a similar quality improvement intervention that Dr. Neighbors co-leads with Ms. Lincourt, Association Commissioner of OASAS.

Dr. Ramsey will lead the development of regulatory and clinical best practice content for the OTP intervention. She will also lead communication efforts with the OTPs as well as with other national and state regulatory bodies overseeing OTP policies. Dr. Ramsey will only work on administrative research analysis and will have no involvement in recruitment.

Dr. Bao will lead the development and implementation of the financial decision support components of the intervention. She will also lead the investigative team’s efforts in conducting analyses of New York State administrative data to understand OTP uptake of bundled payment and relationship with uptake of THD. Dr. Bao will only work on administrative research analysis and will have no involvement in recruitment.

Dr. Megan O’Grady will participate as a Sub-Investigator at the University of Connecticut. Dr. O’Grady will be involved in interviewing and consenting clients. Templates for site-related documents in the consent process have been submitted for the University of Connecticut to approve.

Dr. Neighbors, Dr. Ramsey and Dr. Bao will provide oversight of the entire project and development and implementation of all policies, procedures and processes. In these roles, they will be jointly responsible for the implementation of the R61 and R33 activities and ensure that systems are in place to guarantee institutional compliance with US laws, DHHS and NIH policies (including human research, data and facilities). Together they will define and design intervention components.

### Communication among Principal Investigators

The MPIs will hold bi-weekly meetings to coordinate efforts. The meetings will be used to review the overall study status and to ensure the project is complying with the timeline and goals of the project. Weekly team meetings will be to review progress toward completion of Milestones and status of the Stepped Wedge RCT, including clinic recruitment, intervention activities, and data collection topics.

Dissemination and reporting activities will also be discussed during weekly meetings. Email will be the main method of communication between group meetings. Dr. Neighbors’ office at NYUGSOM is located about 2.5 hours by car or train from Dr. Ramsey’s office in the state capitol, Albany, NY, and in close proximity to Dr. Bao’s office in New York City.

### Plans for Resolution of Conflicts

We do not anticipate that major conflicts in study management will arise given the long, successful history of collaboration of this public-academic partnership. The success to date has relied on mutual trust, respect for the perspectives of all members, and an understanding that collaboration is strengthened by common understanding of challenges that are faced. The MPIs have agreed upon the following conflict resolution approach in the event that major disagreements arise. First, they will try to resolve the conflict through a meeting involving all three MPIs. After a detailed discussion of the issues at hand, the PIs will attempt to reach an agreement—either a compromise or an agreement to move forward with the preferred plan of one of the members. If a PI remains dissatisfied despite this process, the PIs will convene a meeting with all the Co-Is of the project team and the issue in conflict will be presented. All aspects of the decision at hand will be discussed until all members of the team agree to proceed to making a decision. A vote will then be taken among all project team members to choose by majority support the action to be taken.

### Change in PI Location

If a PI moves to a new institution, attempts will be made to transfer the relevant portion of the grant to the new institution. In the event that a PI cannot carry out their duties, a new PI will be recruited as a replacement at one of the participating institutions.

# Conflict of Interest Policy

The independence of this study from any actual or perceived influence, such as by the pharmaceutical industry, is critical. Therefore any actual conflict of interest of persons who have a role in the design, conduct, analysis, publication, or any aspect of this trial will be disclosed and managed. Furthermore, persons who have a perceived conflict of interest will be required to have such conflicts managed in a way that is appropriate to their participation in the trial. The study leadership in conjunction with the <specify NIH IC> has established policies and procedures for all study group members to disclose all conflicts of interest and will establish a mechanism for the management of all reported dualities of interest.

Any investigator who has a conflict of interest with this study (patent ownership, royalties, or financial gain greater than the minimum allowable by their institution, etc.) must have the conflict reviewed by the NYU Langone Conflict of Interest Management Unit (CIMU) with a Committee-sanctioned conflict management plan that has been reviewed and approved by the study sponsor prior to participation in this study. All NYULMC investigators will follow the applicable conflict of interest policies.

1. Creswell JW, Creswell JD. Research Design: Qualitative, Quantitative, and Mixed Methods Approaches. 5th ed. Los Angeles, CA: Sage; 2018. [↑](#endnote-ref-1)
2. Ivankova NV, Creswell JW, Stick SL. Using Mixed-Methods Sequential Explanatory Design: FromTheory to Practice. Field Methods. 2006;18(1):3-20. [↑](#endnote-ref-2)
3. Facing Addiction in America: The Surgeon General's Report on Alcohol, Drugs, and Health. In: General OotS, ed. Washington (DC): U.S. Department of Health & Human Services; November 2016. [↑](#endnote-ref-3)
4. Murthy VH. Ending the Opioid Epidemic - A Call to Action. N Engl J Med. 2016;375(25):2413-2415. [↑](#endnote-ref-4)
5. Volkow ND. Collision of the COVID-19 and Addiction Epidemics. Ann Intern Med. 2020;173(1):61-62. [↑](#endnote-ref-5)
6. Volkow ND, Blanco C. The changing opioid crisis: development, challenges and opportunities. Mol

   Psychiatry. 2021;26(1):218-233. [↑](#endnote-ref-6)
7. Hser YI, Evans E, Grella C, Ling W, Anglin D. Long-term course of opioid addiction. Harv Rev

   Psychiatry. 2015;23(2):76-89. [↑](#endnote-ref-7)
8. Hser YI, Evans E, Huang D, Brecht ML, Li L. Comparing the dynamic course of heroin, cocaine, and

   methamphetamine use over 10 years. Addict Behav. 2008;33(12):1581-1589. [↑](#endnote-ref-8)
9. Hser YI, Gelberg L, Hoffman V, Grella CE, McCarthy W, Anglin MD. Health conditions among aging

   narcotics addicts: medical examination results. J Behav Med. 2004;27(6):607-622. [↑](#endnote-ref-9)
10. Hser YI, Huang D, Chou CP, Anglin MD. Trajectories of heroin addiction: growth mixture modeling

    results based on a 33-year follow-up study. Eval Rev. 2007;31(6):548-563. [↑](#endnote-ref-10)
11. Hser YI, Huang D, Saxon AJ, et al. Distinctive Trajectories of Opioid Use Over an Extended Follow-up

    of Patients in a Multisite Trial on Buprenorphine + Naloxone and Methadone. J Addict Med.

    2017;11(1):63-69. [↑](#endnote-ref-11)
12. Cacciola JS, Dugosh KL, Camilleri AC. Treatment history: relationship to treatment outcomes. Subst

    Use Misuse. 2009;44(3):305-321. [↑](#endnote-ref-12)
13. Dennis ML, Scott CK, Funk R, Foss MA. The duration and correlates of addiction and treatment

    careers. J Subst Abuse Treat. 2005;28 Suppl 1:S51-62. [↑](#endnote-ref-13)
14. Wilson N, Kariisa M, Seth P, Smith Ht, Davis NL. Drug and Opioid-Involved Overdose Deaths - United

    States, 2017-2018. MMWR Morb Mortal Wkly Rep. 2020;69(11):290-297. [↑](#endnote-ref-14)
15. Hughes PM, Verrastro G, Fusco CW, Wilson CG, Ostrach B. An examination of telehealth policy

    impacts on initial rural opioid use disorder treatment patterns during the COVID-19 pandemic. J Rural

    Health. 2021;37(3):467-472. [↑](#endnote-ref-15)
16. Goedel WC, Shapiro A, Cerdá M, Tsai JW, Hadland SE, Marshall BDL. Association of Racial/Ethnic

    Segregation With Treatment Capacity for Opioid Use Disorder in Counties in the United States. JAMA

    Netw Open. 2020;3(4):e203711. [↑](#endnote-ref-16)
17. Friedman J, Hansen H. Far From a "White Problem": Responding to the Overdose Crisis as a Racial

    Justice Issue. Am J Public Health. 2022;112(S1):S30-s32. [↑](#endnote-ref-17)
18. Friedman J, Beletsky L, Jordan A. Surging Racial Disparities in the U.S. Overdose Crisis. Am J

    Psychiatry. 2022;179(2):166-169. [↑](#endnote-ref-18)
19. Townsend T, Kline D, Rivera-Aguirre A, et al. Racial/Ethnic and Geographic Trends in Combined

    Stimulant/Opioid Overdoses, 2007-2019. Am J Epidemiol. 2022. [↑](#endnote-ref-19)
20. Jordan A, Mathis M, Haeny A, Funaro M, Paltin D, Ransome Y. An Evaluation of Opioid Use in Black

    Communities: A Rapid Review of the Literature. Harv Rev Psychiatry. 2021;29(2):108-130. [↑](#endnote-ref-20)
21. Lippold KM, Jones CM, Olsen EO, Giroir BP. Racial/Ethnic and Age Group Differences in Opioid and

    Synthetic Opioid-Involved Overdose Deaths Among Adults Aged ≥18 Years in Metropolitan Areas -

    United States, 2015-2017. MMWR Morb Mortal Wkly Rep. 2019;68(43):967-973. [↑](#endnote-ref-21)
22. Mason M, Soliman R, Kim HS, Post LA. Disparities by Sex and Race and Ethnicity in Death Rates Due

    to Opioid Overdose Among Adults 55 Years or Older, 1999 to 2019. JAMA Netw Open.

    2022;5(1):e2142982 [↑](#endnote-ref-22)
23. Mattick RP, Breen C, Kimber J, Davoli M. Methadone maintenance therapy versus no opioid

    replacement therapy for opioid dependence. Cochrane Database Syst Rev. 2009(3):CD002209. [↑](#endnote-ref-23)
24. Kleber HD. Methadone Maintenance 4 Decades Later. JAMA. 2008;300(19):2303. [↑](#endnote-ref-24)
25. Dole VP. A Medical Treatment for Diacetylmorphine (Heroin) Addiction. JAMA. 1965;193(8):646. [↑](#endnote-ref-25)
26. Dole VP. Methadone Maintenance Treatment for 25,000 Heroin Addicts. JAMA: The Journal of the

    American Medical Association. 1971;215(7):1131. [↑](#endnote-ref-26)
27. Mattick RP, Breen C, Kimber J, Davoli M. Buprenorphine maintenance versus placebo or methadone

    maintenance for opioid dependence. Cochrane Database Syst Rev. 2014(2):CD002207. [↑](#endnote-ref-27)
28. Gryczynski J, Mitchell SG, Jaffe JH, et al. Retention in methadone and buprenorphine treatment among

    African Americans. J Subst Abuse Treat. 2013;45(3):287-292. [↑](#endnote-ref-28)
29. Medications for Opioid Use Disorder Save Lives. Washington, DC: National Academies of Science,

    Engineering, and Medicine; 2019. [↑](#endnote-ref-29)
30. Earnshaw V, Smith L, Copenhaver M. Drug Addiction Stigma in the Context of Methadone Maintenance Therapy: An Investigation into Understudied Sources of Stigma. Int J Ment Health Addict. 2013;11(1):110-112 [↑](#endnote-ref-30)
31. Jackson DS, Nguemeni Tiako MJ, Jordan A. Disparities in Addiction Treatment: Learning from the Past to Forge an Equitable Future. Med Clin North Am. 2022;106(1):29-41. [↑](#endnote-ref-31)
32. Peterkin A, Davis CS, Weinstein Z. Permanent Methadone Treatment Reform Needed to Combat the Opioid Crisis and Structural Racism. J Addict Med. 2021. [↑](#endnote-ref-32)
33. Hansen HB, Siegel CE, Case BG, Bertollo DN, Dirocco D, Galanter M. Variation in Use of Buprenorphine and Methadone Treatment by Racial, Ethnic, and Income Characteristics of Residential Social Areas in New York City. The Journal of Behavioral Health Services & Research. 2013;40(3):367- 377. [↑](#endnote-ref-33)
34. Hansen H, Siegel C, Wanderling J, Dirocco D. Buprenorphine and methadone treatment for opioid dependence by income, ethnicity and race of neighborhoods in New York City. Drug and Alcohol Dependence. 2016;164:14-21. [↑](#endnote-ref-34)
35. Davis CS, Carr DH. Legal and policy changes urgently needed to increase access to opioid agonist therapy in the United States. Int J Drug Policy. 2019;73:42-48. [↑](#endnote-ref-35)
36. Allen B, Nolan ML, Paone D. Underutilization of medications to treat opioid use disorder: What role does stigma play? Subst Abus. 2019;40(4):459-465. [↑](#endnote-ref-36)
37. Joudrey PJ, Bart G, Brooner RK, et al. Research priorities for expanding access to methadone treatment for opioid use disorder in the United States: A National Institute on Drug Abuse Center for Clinical Trials Network Task Force report. Subst Abus. 2021;42(3):245-254. [↑](#endnote-ref-37)
38. Frank D, Mateu-Gelabert P, Perlman DC, Walters SM, Curran L, Guarino H. "It's like 'liquid handcuffs": The effects of take-home dosing policies on Methadone Maintenance Treatment (MMT) patients' lives. Harm Reduct J. 2021;18(1):88. [↑](#endnote-ref-38)
39. Shover CL, Abraham A, D'Aunno T, Friedmann PD, Humphreys K. The relationship of Medicaid expansion to psychiatric comorbidity care within substance use disorder treatment programs. J Subst Abuse Treat. 2019;105:44-50. [↑](#endnote-ref-39)
40. Frimpong JA, D'Aunno T, Helleringer S, Metsch LR. Low Rates of Adoption and Implementation of Rapid HIV Testing in Substance Use Disorder Treatment Programs. J Subst Abuse Treat. 2016;63:46- 53. [↑](#endnote-ref-40)
41. Frimpong JA, D'Aunno T, Jiang L. Determinants of the availability of hepatitis C testing services in opioid treatment programs: results from a national study. Am J Public Health. 2014;104(6):e75-82. [↑](#endnote-ref-41)
42. Pollack HA, D'Aunno T. HIV testing and counseling in the nation's outpatient substance abuse treatment system, 1995-2005. J Subst Abuse Treat. 2010;38(4):307-316. [↑](#endnote-ref-42)
43. D'Aunno T. The role of organization and management in substance abuse treatment: Review and roadmap. J Subst Abuse Treat. 2006;31(3):221-233. [↑](#endnote-ref-43)
44. Friedmann PD, Lemon SC, Durkin EM, D'Aunno TA. Trends in comprehensive service availability in outpatient drug abuse treatment. Journal of substance abuse treatment. 2003;24(1):81-88. [↑](#endnote-ref-44)
45. D'Aunno T, Vaughn TE. An organizational analysis of service patterns in outpatient drug abuse treatment units. J Subst Abuse. 1995;7(1):27-42. [↑](#endnote-ref-45)
46. Goedel WC, Shapiro A, Cerdá M, Tsai JW, Hadland SE, Marshall BDL. Association of Racial/Ethnic

    Segregation With Treatment Capacity for Opioid Use Disorder in Counties in the United States. JAMA

    Netw Open. 2020;3(4):e203711. [↑](#endnote-ref-46)
47. Hansen HB, Siegel CE, Case BG, Bertollo DN, Dirocco D, Galanter M. Variation in Use of Buprenorphine and Methadone Treatment by Racial, Ethnic, and Income Characteristics of Residential Social Areas in New York City. The Journal of Behavioral Health Services & Research. 2013;40(3):367- 377. [↑](#endnote-ref-47)
48. Hansen H, Siegel C, Wanderling J, Dirocco D. Buprenorphine and methadone treatment for opioid dependence by income, ethnicity and race of neighborhoods in New York City. Drug and Alcohol Dependence. 2016;164:14-21. [↑](#endnote-ref-48)
49. Hollander MAG, Chang CH, Douaihy AB, Hulsey E, Donohue JM. Racial inequity in medication treatment for opioid use disorder: Exploring potential facilitators and barriers to use. Drug Alcohol Depend. 2021;227:108927. [↑](#endnote-ref-49)
50. Schuler MS, Dick AW, Stein BD. Growing racial/ethnic disparities in buprenorphine distribution in the United States, 2007-2017. Drug Alcohol Depend. 2021;223:108710. [↑](#endnote-ref-50)
51. Entress RM. The intersection of race and opioid use disorder treatment: A quantitative analysis. J Subst Abuse Treat. 2021;131:108589. [↑](#endnote-ref-51)
52. Goedel WC, Shapiro A, Cerdá M, Tsai JW, Hadland SE, Marshall BDL. Association of Racial/Ethnic

    Segregation With Treatment Capacity for Opioid Use Disorder in Counties in the United States. JAMA

    Netw Open. 2020;3(4):e203711. [↑](#endnote-ref-52)
53. Hansen HB, Siegel CE, Case BG, Bertollo DN, Dirocco D, Galanter M. Variation in Use of Buprenorphine and Methadone Treatment by Racial, Ethnic, and Income Characteristics of Residential Social Areas in New York City. The Journal of Behavioral Health Services & Research. 2013;40(3):367- 377. [↑](#endnote-ref-53)
54. Manhapra A, Quinones L, Rosenheck R. Characteristics of veterans receiving buprenorphine vs. methadone for opioid use disorder nationally in the Veterans Health Administration. Drug Alcohol Depend. 2016;160:82-89. [↑](#endnote-ref-54)
55. Stahler GJ, Mennis J, Baron DA. Racial/ethnic disparities in the use of medications for opioid use disorder (MOUD) and their effects on residential drug treatment outcomes in the US. Drug Alcohol Depend. 2021;226:108849. [↑](#endnote-ref-55)
56. Lagisetty PA, Ross R, Bohnert A, Clay M, Maust DT. Buprenorphine Treatment Divide by Race/Ethnicity and Payment. JAMA Psychiatry. 2019;76(9):979-981. [↑](#endnote-ref-56)
57. Kilaru AS, Xiong A, Lowenstein M, et al. Incidence of Treatment for Opioid Use Disorder Following Nonfatal Overdose in Commercially Insured Patients. JAMA Network Open. 2020;3(5):e205852. [↑](#endnote-ref-57)
58. D’Aunno T, Pollack HA, Frimpong JA, Wuchiett D. Evidence-based treatment for opioid disorders: A 23- year national study of methadone dose levels. Journal of Substance Abuse Treatment. 2014;47(4):245- 250. [↑](#endnote-ref-58)
59. D'Aunno T. Variations in Methadone Treatment Practices. JAMA. 1992;267(2):253. [↑](#endnote-ref-59)
60. D'Aunno T, Park SE, Pollack HA. Evidence-based treatment for opioid use disorders: A national study

    of methadone dose levels, 2011–2017. Journal of Substance Abuse Treatment. 2019;96:18-22. [↑](#endnote-ref-60)
61. Entress RM. The intersection of race and opioid use disorder treatment: A quantitative analysis. J Subst Abuse Treat. 2021;131:108589. [↑](#endnote-ref-61)
62. Guerrero EG. Enhancing access and retention in substance abuse treatment: the role of Medicaid

    payment acceptance and cultural competence. Drug Alcohol Depend. 2013;132(3):555-561. [↑](#endnote-ref-62)
63. Guerrero E, Amaro H, Kong Y, Khachikian T, Marsh JC. Gender disparities in opioid treatment progress

    in methadone versus counseling. Subst Abuse Treat Prev Policy. 2021;16(1):52. [↑](#endnote-ref-63)
64. Mennis J, Stahler GJ. Racial and Ethnic Disparities in Outpatient Substance Use Disorder Treatment

    Episode Completion for Different Substances. J Subst Abuse Treat. 2016;63:25-33. [↑](#endnote-ref-64)
65. Stahler GJ, Mennis J. Treatment outcome disparities for opioid users: Are there racial and ethnic

    differences in treatment completion across large US metropolitan areas? Drug Alcohol Depend.

    2018;190:170-178. [↑](#endnote-ref-65)
66. Gaither JR, Gordon K, Crystal S, et al. Racial disparities in discontinuation of long-term opioid therapy

    following illicit drug use among black and white patients. Drug Alcohol Depend. 2018;192:371-376. [↑](#endnote-ref-66)
67. Davis CS, Samuels EA. Opioid Policy Changes During the COVID-19 Pandemic - and Beyond. J Addict

    Med. 2020;14(4):e4-e5. [↑](#endnote-ref-67)
68. Eaves E, Trotter R, 2nd, Baldwin J. Another silver lining?: Anthropological perspectives on the promise

    and practice of relaxed restrictions for telemedicine and medication-assisted treatment in the context of

    COVID-19. Hum Organ. 2020;79(4):292-303. [↑](#endnote-ref-68)
69. Joudrey PJ, Bart G, Brooner RK, et al. Research priorities for expanding access to methadone treatment for opioid use disorder in the United States: A National Institute on Drug Abuse Center for Clinical Trials Network Task Force report. Subst Abus. 2021;42(3):245-254. [↑](#endnote-ref-69)
70. Davis CS, Samuels EA. Opioid Policy Changes During the COVID-19 Pandemic - and Beyond. J Addict

    Med. 2020;14(4):e4-e5. [↑](#endnote-ref-70)
71. Administration SAaMHS. Statutes, Regulations, and Guidelines. Medication-Assisted Treatment Web

    site. https://www.samhsa.gov/medication-assisted-treatment/statutes-regulations-guidelines. Published

    2022. Updated 2022/02/01/. Accessed. [↑](#endnote-ref-71)
72. Center for Substance Abuse T. SAMHSA/CSAT Treatment Improvement Protocols. In: Medication-

    Assisted Treatment for Opioid Addiction in Opioid Treatment Programs. Rockville (MD): Substance

    Abuse and Mental Health Services Administration (US); 2005. [↑](#endnote-ref-72)
73. 42 CFR § 8.12 - Federal opioid treatment standards. Cornell Law School.

    https://www.law.cornell.edu/cfr/text/42/8.12. Accessed February 5, 2022. [↑](#endnote-ref-73)
74. Madden EF, Christian BT, Lagisetty PA, Ray BR, Sulzer SH. Treatment provider perceptions of take-

    home methadone regulation before and during COVID-19. Drug Alcohol Depend. 2021;228:109100. [↑](#endnote-ref-74)
75. Larance B, Carragher N, Mattick RP, Lintzeris N, Ali R, Degenhardt L. A latent class analysis of self-

    reported clinical indicators of psychosocial stability and adherence among opioid substitution therapy

    patients: do stable patients receive more unsupervised doses? Drug Alcohol Depend. 2014;142:46-55. [↑](#endnote-ref-75)
76. Frank D, Mateu-Gelabert P, Perlman DC, Walters SM, Curran L, Guarino H. "It's like 'liquid handcuffs": The effects of take-home dosing policies on Methadone Maintenance Treatment (MMT) patients' lives. Harm Reduct J. 2021;18(1):88. [↑](#endnote-ref-76)
77. Anstice S, Strike CJ, Brands B. Supervised methadone consumption: client issues and stigma. Subst

    Use Misuse. 2009;44(6):794-808. [↑](#endnote-ref-77)
78. Deering DEA, Sheridan J, Sellman JD, et al. Consumer and treatment provider perspectives on

    reducing barriers to opioid substitution treatment and improving treatment attractiveness. Addict Behav.

    2011;36(6):636-642. [↑](#endnote-ref-78)
79. Treloar C, Fraser S, Valentine K. Valuing methadone takeaway doses: The contribution of service-user

    perspectives to policy and practice. Drugs: Education, Prevention and Policy. 2007;14(1):61-74. [↑](#endnote-ref-79)
80. Amiri S, Lutz RB, McDonell MG, Roll JM, Amram O. Spatial access to opioid treatment program and

    alcohol and cannabis outlets: analysis of missed doses of methadone during the first, second, and third

    90 days of treatment. Am J Drug Alcohol Abuse. 2020;46(1):78-87. [↑](#endnote-ref-80)
81. Joudrey PJ, Edelman EJ, Wang EA. Drive Times to Opioid Treatment Programs in Urban and Rural

    Counties in 5 US States. Jama. 2019;322(13):1310-1312. [↑](#endnote-ref-81)
82. Kiang MV, Barnett ML, Wakeman SE, Humphreys K, Tsai AC. Robustness of estimated access to

    opioid use disorder treatment providers in rural vs. urban areas of the United States. Drug Alcohol

    Depend. 2021;228:109081. [↑](#endnote-ref-82)
83. Lister JJ, Lister HH. Improving methadone access for rural communities in the USA: lessons learned

    from COVID-19 adaptations and international models of care. Rural Remote Health. 2021;21(4):6770. [↑](#endnote-ref-83)
84. D'Aunno T. Variations in Methadone Treatment Practices. JAMA. 1992;267(2):253. [↑](#endnote-ref-84)
85. Nolan S, Hayashi K, Milloy MJ, et al. The impact of low-threshold methadone maintenance treatment

    on mortality in a Canadian setting. Drug Alcohol Depend. 2015;156:57-61. [↑](#endnote-ref-85)
86. Langendam MW, van Brussel GH, Coutinho RA, van Ameijden EJ. The impact of harm-reduction- based methadone treatment on mortality among heroin users. Am J Public Health. 2001;91(5):774-780. [↑](#endnote-ref-86)
87. Fraser S, valentine k, Treloar C, Macmillan K. Methadone maintenance treatment in New South Wales and Victoria: Takeaways, diversion and other key issues. Sydney, Australia: National Centre in HIV Social Research;2007. [↑](#endnote-ref-87)
88. Strike C, Millson M, Hopkins S, Smith C. What is low threshold methadone maintenance treatment? Int J Drug Policy. 2013;24(6):e51-56. [↑](#endnote-ref-88)
89. Priest KC, Gorfinkel L, Klimas J, Jones AA, Fairbairn N, McCarty D. Comparing Canadian and United States opioid agonist therapy policies. Int J Drug Policy. 2019;74:257-265. [↑](#endnote-ref-89)
90. Peles E, Schreiber S, Sason A, Adelson M. Earning "take-home" privileges and long-term outcome in a methadone maintenance treatment program. J Addict Med. 2011;5(2):92-98. [↑](#endnote-ref-90)
91. Adelson M, Schreiber S, Sason A, Peles E. Are 2 weeks of "take-home" privileges beneficial for patients' long-term outcome in a methadone maintenance treatment program? J Addict Med. 2014;8(3):170-175. [↑](#endnote-ref-91)
92. Administration DE. Methadone. DEA Diversion Control Division: Drug & Chemical Evaluation Section;2019. [↑](#endnote-ref-92)
93. Jones CM, Baldwin GT, Manocchio T, White JO, Mack KA. Trends in Methadone Distribution for Pain Treatment, Methadone Diversion, and Overdose Deaths - United States, 2002-2014. MMWR Morb Mortal Wkly Rep. 2016;65(26):667-671. [↑](#endnote-ref-93)
94. Treatment CfSA. Methadone-Associated Mortality: Background Briefing Report. Center for Substance Abuse Treatment, Substance Abuse and Mental Health Services Administration;2004. [↑](#endnote-ref-94)
95. Methadone Take-Home Flexibilities Extension Guidance. SAMHSA. https://www.samhsa.gov/medication-assisted-treatment/statutes-regulations-guidelines/methadone- guidance. Published 2021. Accessed February 5, 2022. [↑](#endnote-ref-95)
96. Andraka-Christou B, Bouskill K, Haffajee RL, et al. Common themes in early state policy responses to substance use disorder treatment during COVID-19. Am J Drug Alcohol Abuse. 2021;47(4):486-496. [↑](#endnote-ref-96)
97. Nesoff ED, Marziali ME, Martins SS. The estimated impact of state-level support for expanded delivery of substance use disorder treatment during the COVID-19 pandemic. Addiction. 2021. [↑](#endnote-ref-97)
98. Jackson JR, Harle CA, Silverman RD, Simon K, Menachemi N. Characterizing variability in state-level regulations governing opioid treatment programs. J Subst Abuse Treat. 2020;115:108008. [↑](#endnote-ref-98)
99. Madden EF, Christian BT, Lagisetty PA, Ray BR, Sulzer SH. Treatment provider perceptions of take-

    home methadone regulation before and during COVID-19. Drug Alcohol Depend. 2021;228:109100. [↑](#endnote-ref-99)
100. Treitler PC, Bowden CF, Lloyd J, Enich M, Nyaku AN, Crystal S. Perspectives of opioid use disorder treatment providers during COVID-19: Adapting to flexibilities and sustaining reforms. J Subst Abuse Treat. 2022;132:108514. [↑](#endnote-ref-100)
101. Hatch-Maillette MA, Peavy KM, Tsui JI, Banta-Green CJ, Woolworth S, Grekin P. Re-thinking patient stability for methadone in opioid treatment programs during a global pandemic: Provider perspectives. Journal of Substance Abuse Treatment. 2021;124:108223. [↑](#endnote-ref-101)
102. Goldsamt LA, Rosenblum A, Appel P, Paris P, Nazia N. The impact of COVID-19 on opioid treatment programs in the United States. Drug Alcohol Depend. 2021;228:109049. [↑](#endnote-ref-102)
103. Hunter SB, Dopp AR, Ober AJ, Uscher-Pines L. Clinician perspectives on methadone service delivery and the use of telemedicine during the COVID-19 pandemic: A qualitative study. J Subst Abuse Treat. 2021;124:108288. [↑](#endnote-ref-103)
104. Levander XA, Pytell JD, Stoller KB, Korthuis PT, Chander G. COVID-19-related policy changes for methadone take-home dosing: A multistate survey of opioid treatment program leadership. Subst Abus. 2022;43(1):633-639. [↑](#endnote-ref-104)
105. Amram O, Amiri S, Panwala V, Lutz R, Joudrey PJ, Socias E. The impact of relaxation of methadone take-home protocols on treatment outcomes in the COVID-19 era. Am J Drug Alcohol Abuse. 2021;47(6):722-729. [↑](#endnote-ref-105)
106. Trujols J, Larrabeiti A, Sànchez O, Madrid M, De Andrés S, Duran-Sindreu S. Increased flexibility in methadone take-home scheduling during the COVID-19 pandemic: Should this practice be incorporated into routine clinical care? J Subst Abuse Treat. 2020;119:108154. [↑](#endnote-ref-106)
107. Brothers S, Viera A, Heimer R. Changes in methadone program practices and fatal methadone overdose rates in Connecticut during COVID-19. J Subst Abuse Treat. 2021;131:108449. [↑](#endnote-ref-107)
108. Figgatt MC, Salazar Z, Day E, Vincent L, Dasgupta N. Take-home dosing experiences among persons receiving methadone maintenance treatment during COVID-19. J Subst Abuse Treat. 2021;123:108276. [↑](#endnote-ref-108)
109. Hazan J, Congdon L, Sathanandan S, Grewal P. An analysis of initial service transformation in response to the COVID-19 pandemic in two inner-city substance misuse services. Journal of Substance Use. 2021;26(3):275-279. [↑](#endnote-ref-109)
110. Saloner B, Krawczyk N, Solomon K, et al. Experiences with substance use disorder treatment during the COVID-19 pandemic: Findings from a multistate survey. Int J Drug Policy. 2021;101:103537. [↑](#endnote-ref-110)
111. Gomes T, Campbell TJ, Kitchen SA, et al. Association Between Increased Dispensing of Opioid Agonist Therapy Take-Home Doses and Opioid Overdose and Treatment Interruption and Discontinuation. Jama. 2022;327(9):846-855. [↑](#endnote-ref-111)
112. Joseph G, Torres-Lockhart K, Stein MR, Mund PA, Nahvi S. Reimagining patient-centered care in opioid treatment programs: Lessons from the Bronx during COVID-19. J Subst Abuse Treat. 2021;122:108219. [↑](#endnote-ref-112)
113. Tracy K, Wachtel L, Friedman T. The impact of COVID-19 on opioid treatment program (OTP) services: Where do we go from here? J Subst Abuse Treat. 2021;131:108394. [↑](#endnote-ref-113)
114. Gomes T, Campbell TJ, Kitchen SA, et al. Association Between Increased Dispensing of Opioid Agonist Therapy Take-Home Doses and Opioid Overdose and Treatment Interruption and Discontinuation. Jama. 2022;327(9):846-855. [↑](#endnote-ref-114)
115. Joudrey PJ, Bart G, Brooner RK, et al. Research priorities for expanding access to methadone treatment for opioid use disorder in the United States: A National Institute on Drug Abuse Center for Clinical Trials Network Task Force report. Subst Abus. 2021;42(3):245-254. [↑](#endnote-ref-115)
116. Nunes EV, Levin FR, Reilly MP, El-Bassel N. Medication treatment for opioid use disorder in the age of COVID-19: Can new regulations modify the opioid cascade? J Subst Abuse Treat. 2021;122:108196. [↑](#endnote-ref-116)
117. Kilbourne AM, Switzer G, Hyman K, Crowley-Matoka M, Fine MJ. Advancing Health Disparities Research Within the Health Care System: A Conceptual Framework. Am J Public Health. 2006;96(12):2113-2121. [↑](#endnote-ref-117)
118. Harvey G, Kitson AJIS. PARIHS revisited: from heuristic to integrated framework for the successful implementation of knowledge into practice. 2016;11(1):33. [↑](#endnote-ref-118)
119. Swindle T, Johnson SL, Whiteside-Mansell L, Curran GM. A mixed methods protocol for developing and testing implementation strategies for evidence-based obesity prevention in childcare: a cluster randomized hybrid type III trial. Implementation Science. 2017;12(1):90. [↑](#endnote-ref-119)
120. Kitson A, Harvey G. FACILITATING AN EVIDENCE-BASED INNOVATION INTO PRACTICE. Implementing evidence-based practice in healthcare: a facilitation guide. 2015:85. [↑](#endnote-ref-120)
121. Institute of Medicine. Improving the Quality of Health Care for Mental and Substance-Use Conditions. Washington, D.C.: National Academies Press; 2006. [↑](#endnote-ref-121)
122. Office of Surgeon General. In: Facing Addiction in America: The Surgeon General's Report on Alcohol, Drugs, and Health. Washington (DC): US Department of Health and Human Services; 2016. [↑](#endnote-ref-122)
123. Padwa H, Urada D, Gauthier P, et al. Organizing Publicly Funded Substance Use Disorder Treatment in the United States: Moving Toward a Service System Approach. J Subst Abuse Treat. 2016;69:9-18. [↑](#endnote-ref-123)
124. National Center on Addiction and Substance Abuse at Columbia University. Addiciton medicine: Closing the gap between science and practice author; June 2012 2012. [↑](#endnote-ref-124)
125. England MJ, Butler AS, Gonzalez ML. Psychosocial interventions for mental and substance use disorders: A framework for establishing evidence-based standards. National Academy Press; 2015. [↑](#endnote-ref-125)
126. McLellan AT, Lewis DC, O'Brien CP, Kleber HD. Drug dependence, a chronic medical illness: implications for treatment, insurance, and outcomes evaluation. Jama. 2000;284(13):1689-1695. [↑](#endnote-ref-126)
127. Institute of Medicine. Improving the Quality of Health Care for Mental and Substance-Use Conditions. Washington, D.C.: National Academies Press; 2006. [↑](#endnote-ref-127)
128. National Center on Addiction and Substance Abuse at Columbia University. Addiciton medicine: Closing the gap between science and practice author; June 2012 2012. [↑](#endnote-ref-128)
129. England MJ, Butler AS, Gonzalez ML. Psychosocial interventions for mental and substance use disorders: A framework for establishing evidence-based standards. National Academy Press; 2015. [↑](#endnote-ref-129)
130. O'Grady MA, Lincourt P, Gilmer E, et al. How are Substance Use Disorder Treatment Programs Adjusting to Value-Based Payment? A Statewide Qualitative Study. Subst Abuse. 2020;14:1178221820924026. [↑](#endnote-ref-130)
131. O'Grady MA, Lincourt P, Greenfield B, et al. A facilitation model for implementing quality improvement practices to enhance outpatient substance use disorder treatment outcomes: a stepped-wedge randomized controlled trial study protocol. Implement Sci. 2021;16(1):5. [↑](#endnote-ref-131)
132. Hunter SB, Ober AJ, Paddock SM, Hunt PE, Levan D. Continuous quality improvement (CQI) in addiction treatment settings: design and intervention protocol of a group randomized pilot study. Addiction Science & Clinical Practice. 2014;9(1):4. [↑](#endnote-ref-132)
133. Hunter SB, Rutter CM, Ober AJ, Booth MS. Building capacity for continuous quality improvement (CQI): A pilot study. Journal of substance abuse treatment. 2017;81:44-52. [↑](#endnote-ref-133)
134. Quanbeck AR, Madden L, Edmundson E, et al. A business case for quality improvement in addiction treatment: evidence from the NIATx collaborative. The journal of behavioral health services & research. 2012;39(1):91-100. [↑](#endnote-ref-134)
135. McCarty D, Gustafson D, Capoccia VA, Cotter F. Improving care for the treatment of alcohol and drug disorders. The journal of behavioral health services & research. 2009;36(1):52-60. [↑](#endnote-ref-135)
136. Crèvecoeur-MacPhail D, Bellows A, Rutkowski BA, Ransom L, Myers AC, Rawson RA. “I've been NIATxed”: Participants' Experience with Process Improvement. Journal of psychoactive drugs. 2010;42(sup6):249-259. [↑](#endnote-ref-136)
137. Gustafson DH, Quanbeck AR, Robinson JM, et al. Which elements of improvement collaboratives are most effective? A cluster-randomized trial. Addiction. 2013;108(6):1145-1157. [↑](#endnote-ref-137)
138. McCarty D, Gustafson DH, Wisdom JP, et al. The Network for the Improvement of Addiction Treatment (NIATx): enhancing access and retention. Drug Alcohol Depend. 2007;88(2-3):138-145. [↑](#endnote-ref-138)
139. Fields D, Knudsen HK, Roman PM. Implementation of Network for the Improvement of Addiction Treatment (NIATx) processes in substance use disorder treatment centers. The journal of behavioral health services & research. 2016;43(3):354-365. [↑](#endnote-ref-139)
140. Joseph G, Torres-Lockhart K, Stein MR, Mund PA, Nahvi S. Reimagining patient-centered care in opioid treatment programs: Lessons from the Bronx during COVID-19. J Subst Abuse Treat. 2021;122:108219. [↑](#endnote-ref-140)
141. Bao Y, Williams AR, Schackman BR. COVID-19 Could Change the Way We Respond to the Opioid Crisis-for the Better. Psychiatr Serv. 2020;71(12):1214-1215. [↑](#endnote-ref-141)
142. Bao Y, Li Y, Jeng PJ, et al. Design of a Payment Decision-Support Tool for Coordinated Specialty Care for Early Psychosis. Psychiatr Serv. 2021;72(2):180-185. [↑](#endnote-ref-142)
143. Creswell JW, Creswell JD. Research Design: Qualitative, Quantitative, and Mixed Methods Approaches. 5th ed. Los Angeles, CA: Sage; 2018. [↑](#endnote-ref-143)
144. Ivankova NV, Creswell JW, Stick SL. Using Mixed-Methods Sequential Explanatory Design: FromTheory to Practice. Field Methods. 2006;18(1):3-20. [↑](#endnote-ref-144)
145. NIH Office of Behavioral and Social Sciences. Best Practices for Mixed Methods Research in the Health Sciences. 2nd ed. Bethesda, MD: Naional Institutes of Health; 2018. [↑](#endnote-ref-145)
146. Creswell JW, Creswell JD. Research Design: Qualitative, Quantitative, and Mixed Methods Approaches. 5th ed. Los Angeles, CA: Sage; 2018. [↑](#endnote-ref-146)
147. Ivankova NV, Creswell JW, Stick SL. Using Mixed-Methods Sequential Explanatory Design: FromTheory to Practice. Field Methods. 2006;18(1):3-20. [↑](#endnote-ref-147)
148. Little RA, Rubin DB. Statistical Analysis with Missing Data. New York, NY: Wiley; 1987. [↑](#endnote-ref-148)
149. Raghunathan TE. What do we do with missing data? Some options for analysis of incomplete data.

     Annu Rev Public Health. 2004;25:99-117. [↑](#endnote-ref-149)
150. Robins JM, Rotnitzky A, Zhao LP. Estimation of Regression-Coefficients When Some Regressors Are

     Not Always Observed. Journal of the American Statistical Association. 1994;89(427):846-866. [↑](#endnote-ref-150)
151. MacKenzie TA, Grunkemeier GL, Grunwald GK, et al. A primer on using shrinkage to compare in-

     hospital mortality between centers. Ann Thorac Surg. 2015;99(3):757-761. [↑](#endnote-ref-151)
152. Cohen ME, Ko CY, Bilimoria KY, et al. Optimizing ACS NSQIP modeling for evaluation of surgical

     quality and risk: patient risk adjustment, procedure mix adjustment, shrinkage adjustment, and surgical

     focus. J Am Coll Surg. 2013;217(2):336-346.e331. [↑](#endnote-ref-152)
153. George EI, Ročková V, Rosenbaum PR, Satopää VA, Silber JH. Mortality Rate Estimation and

     Standardization for Public Reporting: Medicare’s Hospital Compare. Journal of the American Statistical

     Association. 2017;112(519):15. [↑](#endnote-ref-153)
154. Varewyck M, Goetghebeur E, Eriksson M, Vansteelandt S. On shrinkage and model extrapolation in the

     evaluation of clinical center performance. Biostatistics. 2014;15(4):651-664. [↑](#endnote-ref-154)
155. Woodward EN, Matthieu MM, Uchendu US, Rogal S, Kirchner JE. The health equity implementation

     framework: proposal and preliminary study of hepatitis C virus treatment. Implement Sci. 2019;14(1):26. [↑](#endnote-ref-155)
156. Woodward EN, Singh RS, Ndebele-Ngwenya P, Melgar Castillo A, Dickson KS, Kirchner JE. A more

     practical guide to incorporating health equity domains in implementation determinant frameworks.

     Implement Sci Commun. 2021;2(1):61. [↑](#endnote-ref-156)
157. Hsieh HF, Shannon SE. Three approaches to qualitative content analysis. Qual Health Res.

     2005;15(9):1277-1288. [↑](#endnote-ref-157)
158. Shelley DR, Ogedegbe G, Anane S, et al. Testing the use of practice facilitation in a cluster randomized stepped-wedge design trial to improve adherence to cardiovascular disease prevention guidelines: HealthyHearts NYC. Implement Sci. 2016;11(1):88. [↑](#endnote-ref-158)
159. Nutting PA, Crabtree BF, Stewart EE, et al. Effect of facilitation on practice outcomes in the National Demonstration Project model of the patient-centered medical home. Ann Fam Med. 2010;8 Suppl 1:S33-44; S92. [↑](#endnote-ref-159)
160. Solberg LI, Asche SE, Margolis KL, Whitebird RR. Measuring an organization's ability to manage change: the change process capability questionnaire and its use for improving depression care. Am J Med Qual. 2008;23(3):193-200. [↑](#endnote-ref-160)
161. Klein KJ, Sorra JS. The challenge of innovation implementation. The Academy of Management Review. 1996;21(4):1055-1080. [↑](#endnote-ref-161)
162. Klein KJ, Conn AB, Sorra JS. Implementing computerized technology: an organizational analysis. J Appl Psychol. 2001;86(5):811-824. [↑](#endnote-ref-162)
163. Kilbourne AM, Switzer G, Hyman K, Crowley-Matoka M, Fine MJ. Advancing Health Disparities Research Within the Health Care System: A Conceptual Framework. Am J Public Health. 2006;96(12):2113-2121. [↑](#endnote-ref-163)
164. Woodward EN, Matthieu MM, Uchendu US, Rogal S, Kirchner JE. The health equity implementation

     framework: proposal and preliminary study of hepatitis C virus treatment. Implement Sci. 2019;14(1):26. [↑](#endnote-ref-164)
165. Woodward EN, Singh RS, Ndebele-Ngwenya P, Melgar Castillo A, Dickson KS, Kirchner JE. A more

     practical guide to incorporating health equity domains in implementation determinant frameworks.

     Implement Sci Commun. 2021;2(1):61. [↑](#endnote-ref-165)
166. Green CA, McCarty D, Mertens J, et al. A qualitative study of the adoption of buprenorphine for opioid addiction treatment. J Subst Abuse Treat. 2014;46(3):390-401. [↑](#endnote-ref-166)
167. Shelley DR, Ogedegbe G, Anane S, et al. Testing the use of practice facilitation in a cluster randomized stepped-wedge design trial to improve adherence to cardiovascular disease prevention guidelines: HealthyHearts NYC. Implement Sci. 2016;11(1):88. [↑](#endnote-ref-167)
168. Curry L, Nunez-Smith M. Mixed Methods in Health Sciences Research: A Practical Primer. In: Thousand Oaks, California: Sage; 2015: https://methods.sagepub.com/book/mixed-methods-in-health- sciences-research-a-practical-primer. Accessed 2022/03/06. [↑](#endnote-ref-168)
169. Little RA, Rubin DB. Statistical Analysis with Missing Data. New York, NY: Wiley; 1987. [↑](#endnote-ref-169)
170. Raghunathan TE. What do we do with missing data? Some options for analysis of incomplete data.

     Annu Rev Public Health. 2004;25:99-117. [↑](#endnote-ref-170)
171. Robins JM, Rotnitzky A, Zhao LP. Estimation of Regression-Coefficients When Some Regressors Are

     Not Always Observed. Journal of the American Statistical Association. 1994;89(427):846-866. [↑](#endnote-ref-171)
172. Li F, Hughes JP, Hemming K, Taljaard M, Melnick ER, Heagerty PJ. Mixed-effects models for the design and analysis of stepped wedge cluster randomized trials: An overview. Stat Methods Med Res. 2021;30(2):612-639. [↑](#endnote-ref-172)
173. NIH Office of Behavioral and Social Sciences. Best Practices for Mixed Methods Research in the Health Sciences. 2nd ed. Bethesda, MD: Naional Institutes of Health; 2018. [↑](#endnote-ref-173)
174. O'Grady MA, Lincourt P, Greenfield B, et al. A facilitation model for implementing quality improvement practices to enhance outpatient substance use disorder treatment outcomes: a stepped-wedge randomized controlled trial study protocol. Implement Sci. 2021;16(1):5. [↑](#endnote-ref-174)
175. Copas AJ, Lewis JJ, Thompson JA, Davey C, Baio G, Hargreaves JR. Designing a stepped wedge trial: three main designs, carry-over effects and randomisation approaches. Trials. 2015;16:352. [↑](#endnote-ref-175)
176. Barker D, McElduff P, D'Este C, Campbell MJ. Stepped wedge cluster randomised trials: a review of the statistical methodology used and available. BMC Med Res Methodol. 2016;16:69. [↑](#endnote-ref-176)
177. Beard E, Lewis JJ, Copas A, et al. Stepped wedge randomised controlled trials: systematic review of studies published between 2010 and 2014. Trials. 2015;16:353. [↑](#endnote-ref-177)
178. Mdege ND, Man MS, Taylor Nee Brown CA, Torgerson DJ. Systematic review of stepped wedge cluster randomized trials shows that design is particularly used to evaluate interventions during routine implementation. J Clin Epidemiol. 2011;64(9):936-948. [↑](#endnote-ref-178)
179. Taljaard M, Teerenstra S, Ivers NM, Fergusson DA. Substantial risks associated with few clusters in cluster randomized and stepped wedge designs. Clin Trials. 2016;13(4):459-463. [↑](#endnote-ref-179)
180. Woodward EN, Matthieu MM, Uchendu US, Rogal S, Kirchner JE. The health equity implementation

     framework: proposal and preliminary study of hepatitis C virus treatment. Implement Sci. 2019;14(1):26. [↑](#endnote-ref-180)
181. Woodward EN, Singh RS, Ndebele-Ngwenya P, Melgar Castillo A, Dickson KS, Kirchner JE. A more

     practical guide to incorporating health equity domains in implementation determinant frameworks.

     Implement Sci Commun. 2021;2(1):61. [↑](#endnote-ref-181)
182. Stouten J, Rousseau DM, de Cremer D. Successful organizational change: Integrating the management practice and scholarly literatures. The Academy of Management Annals. 2018;12(2):752- 788. [↑](#endnote-ref-182)
183. Wang SY, Groene O. The effectiveness of behavioral economics-informed interventions on physician behavioral change: A systematic literature review. PLoS One. 2020;15(6):e0234149. [↑](#endnote-ref-183)
184. Mostofian F, Ruban C, Simunovic N, Bhandari M. Changing physician behavior: what works? Am J Manag Care. 2015;21(1):75-84. [↑](#endnote-ref-184)
185. Stouten J, Rousseau DM, de Cremer D. Successful organizational change: Integrating the management practice and scholarly literatures. The Academy of Management Annals. 2018;12(2):752- 788. [↑](#endnote-ref-185)
186. Wang SY, Groene O. The effectiveness of behavioral economics-informed interventions on physician behavioral change: A systematic literature review. PLoS One. 2020;15(6):e0234149. [↑](#endnote-ref-186)
187. Mostofian F, Ruban C, Simunovic N, Bhandari M. Changing physician behavior: what works? Am J Manag Care. 2015;21(1):75-84. [↑](#endnote-ref-187)
188. MacKenzie TA, Grunkemeier GL, Grunwald GK, et al. A primer on using shrinkage to compare in-

     hospital mortality between centers. Ann Thorac Surg. 2015;99(3):757-761. [↑](#endnote-ref-188)
189. Cohen ME, Ko CY, Bilimoria KY, et al. Optimizing ACS NSQIP modeling for evaluation of surgical

     quality and risk: patient risk adjustment, procedure mix adjustment, shrinkage adjustment, and surgical

     focus. J Am Coll Surg. 2013;217(2):336-346.e331. [↑](#endnote-ref-189)
190. George EI, Ročková V, Rosenbaum PR, Satopää VA, Silber JH. Mortality Rate Estimation and

     Standardization for Public Reporting: Medicare’s Hospital Compare. Journal of the American Statistical

     Association. 2017;112(519):15. [↑](#endnote-ref-190)
191. Varewyck M, Goetghebeur E, Eriksson M, Vansteelandt S. On shrinkage and model extrapolation in the

     evaluation of clinical center performance. Biostatistics. 2014;15(4):651-664. [↑](#endnote-ref-191)
192. Larance B, Carragher N, Mattick RP, Lintzeris N, Ali R, Degenhardt L. A latent class analysis of self-

     reported clinical indicators of psychosocial stability and adherence among opioid substitution therapy

     patients: do stable patients receive more unsupervised doses? Drug Alcohol Depend. 2014;142:46-55. [↑](#endnote-ref-192)
193. Treitler PC, Bowden CF, Lloyd J, Enich M, Nyaku AN, Crystal S. Perspectives of opioid use disorder treatment providers during COVID-19: Adapting to flexibilities and sustaining reforms. J Subst Abuse Treat. 2022;132:108514. [↑](#endnote-ref-193)
194. Hatch-Maillette MA, Peavy KM, Tsui JI, Banta-Green CJ, Woolworth S, Grekin P. Re-thinking patient stability for methadone in opioid treatment programs during a global pandemic: Provider perspectives. Journal of Substance Abuse Treatment. 2021;124:108223. [↑](#endnote-ref-194)
195. Goldsamt LA, Rosenblum A, Appel P, Paris P, Nazia N. The impact of COVID-19 on opioid treatment programs in the United States. Drug Alcohol Depend. 2021;228:109049. [↑](#endnote-ref-195)
196. Hunter SB, Dopp AR, Ober AJ, Uscher-Pines L. Clinician perspectives on methadone service delivery and the use of telemedicine during the COVID-19 pandemic: A qualitative study. J Subst Abuse Treat. 2021;124:108288. [↑](#endnote-ref-196)
197. Levander XA, Pytell JD, Stoller KB, Korthuis PT, Chander G. COVID-19-related policy changes for methadone take-home dosing: A multistate survey of opioid treatment program leadership. Subst Abus. 2022;43(1):633-639. [↑](#endnote-ref-197)
198. Bao Y, Williams AR, Schackman BR. COVID-19 Could Change the Way We Respond to the Opioid Crisis-for the Better. Psychiatr Serv. 2020;71(12):1214-1215. [↑](#endnote-ref-198)
199. Bao Y, Li Y, Jeng PJ, et al. Design of a Payment Decision-Support Tool for Coordinated Specialty Care for Early Psychosis. Psychiatr Serv. 2021;72(2):180-185. [↑](#endnote-ref-199)
200. Kidorf M, Brooner RK, Dunn KE, Peirce JM. Use of an electronic pillbox to increase number of methadone take-home doses during the COVID-19 pandemic. J Subst Abuse Treat. 2021;126:108328. [↑](#endnote-ref-200)
201. Dunn KE, Brooner RK, Stoller KB. Technology-assisted methadone take-home dosing for dispensing methadone to persons with opioid use disorder during the Covid-19 pandemic. J Subst Abuse Treat. 2021;121:108197. [↑](#endnote-ref-201)
202. Brooklyn JR, Stothart M, Stunell M, Berman VM, Rylant D, Hanson M. Characterizing the Clinical use of a Novel Video-assisted Dosing Protocol With Secure Medication Dispensers to Reduce Barriers to Opioid Treatment. J Addict Med. 2021. [↑](#endnote-ref-202)
203. Institute of Medicine. Improving the Quality of Health Care for Mental and Substance-Use Conditions. Washington, D.C.: National Academies Press; 2006. [↑](#endnote-ref-203)
204. Office of Surgeon General. In: Facing Addiction in America: The Surgeon General's Report on Alcohol, Drugs, and Health. Washington (DC): US Department of Health and Human Services; 2016. [↑](#endnote-ref-204)
205. Padwa H, Urada D, Gauthier P, et al. Organizing Publicly Funded Substance Use Disorder Treatment in the United States: Moving Toward a Service System Approach. J Subst Abuse Treat. 2016;69:9-18. [↑](#endnote-ref-205)
206. National Center on Addiction and Substance Abuse at Columbia University. Addiciton medicine: Closing the gap between science and practice author; June 2012 2012. [↑](#endnote-ref-206)
207. England MJ, Butler AS, Gonzalez ML. Psychosocial interventions for mental and substance use disorders: A framework for establishing evidence-based standards. National Academy Press; 2015. [↑](#endnote-ref-207)
208. McLellan AT, Lewis DC, O'Brien CP, Kleber HD. Drug dependence, a chronic medical illness: implications for treatment, insurance, and outcomes evaluation. Jama. 2000;284(13):1689-1695. [↑](#endnote-ref-208)
209. Little RA, Rubin DB. Statistical Analysis with Missing Data. New York, NY: Wiley; 1987. [↑](#endnote-ref-209)
210. Raghunathan TE. What do we do with missing data? Some options for analysis of incomplete data.

     Annu Rev Public Health. 2004;25:99-117. [↑](#endnote-ref-210)
211. Robins JM, Rotnitzky A, Zhao LP. Estimation of Regression-Coefficients When Some Regressors Are

     Not Always Observed. Journal of the American Statistical Association. 1994;89(427):846-866. [↑](#endnote-ref-211)
212. MacKenzie TA, Grunkemeier GL, Grunwald GK, et al. A primer on using shrinkage to compare in-

     hospital mortality between centers. Ann Thorac Surg. 2015;99(3):757-761. [↑](#endnote-ref-212)
213. Cohen ME, Ko CY, Bilimoria KY, et al. Optimizing ACS NSQIP modeling for evaluation of surgical

     quality and risk: patient risk adjustment, procedure mix adjustment, shrinkage adjustment, and surgical

     focus. J Am Coll Surg. 2013;217(2):336-346.e331. [↑](#endnote-ref-213)
214. George EI, Ročková V, Rosenbaum PR, Satopää VA, Silber JH. Mortality Rate Estimation and

     Standardization for Public Reporting: Medicare’s Hospital Compare. Journal of the American Statistical

     Association. 2017;112(519):15. [↑](#endnote-ref-214)
215. Varewyck M, Goetghebeur E, Eriksson M, Vansteelandt S. On shrinkage and model extrapolation in the

     evaluation of clinical center performance. Biostatistics. 2014;15(4):651-664. [↑](#endnote-ref-215)
216. Little RA, Rubin DB. Statistical Analysis with Missing Data. New York, NY: Wiley; 1987. [↑](#endnote-ref-216)
217. Raghunathan TE. What do we do with missing data? Some options for analysis of incomplete data.

     Annu Rev Public Health. 2004;25:99-117. [↑](#endnote-ref-217)
218. Robins JM, Rotnitzky A, Zhao LP. Estimation of Regression-Coefficients When Some Regressors Are

     Not Always Observed. Journal of the American Statistical Association. 1994;89(427):846-866. [↑](#endnote-ref-218)
219. Li F, Hughes JP, Hemming K, Taljaard M, Melnick ER, Heagerty PJ. Mixed-effects models for the design and analysis of stepped wedge cluster randomized trials: An overview. Stat Methods Med Res. 2021;30(2):612-639. [↑](#endnote-ref-219)
220. Li F, Hughes JP, Hemming K, Taljaard M, Melnick ER, Heagerty PJ. Mixed-effects models for the design and analysis of stepped wedge cluster randomized trials: An overview. Stat Methods Med Res. 2021;30(2):612-639. [↑](#endnote-ref-220)
221. Stroup WW. Generalized linear mixed models : modern concepts, methods and applications. Boca Raton: CRC Press, Taylor & Francis Group; 2013. [↑](#endnote-ref-221)
222. Brooks ME, Kristensen K, van Benthem KJ, et al. glmmTMB Balances Speed and Flexibility Among Packages for Zero-inflated Generalized Linear Mixed Modeling. R J. 2017;9(2):378-400. [↑](#endnote-ref-222)
223. R: A language and environment for statistical computing [computer program]. Vienna, Austria: R Foundation for Statistical Computing; 2021. [↑](#endnote-ref-223)
224. Laster LL, Johnson MF. Non-inferiority trials: the 'at least as good as' criterion. Stat Med. 2003;22(2):187-200. [↑](#endnote-ref-224)
225. Barber JS, Murphy SA, Axinn WG, Maples J. Discrete-time multilevel hazard analysis. Sociol Methodol. 2000;30:201-235. [↑](#endnote-ref-225)
226. Austin PC. A Tutorial on Multilevel Survival Analysis: Methods, Models and Applications. International Statistical Review. 2017;85(2):185-203. [↑](#endnote-ref-226)
227. Steele F. MULTILEVEL DISCRETE-TIME EVENT HISTORY MODELS WITH APPLICATIONS TO THE ANALYSIS OF RECURRENT EMPLOYMENT TRANSITIONS. Australian & New Zealand Journal of Statistics. 2011;53(1):1-20. [↑](#endnote-ref-227)
228. Singer JD, Willett JB. Applied longitudinal data analysis: Modeling change and event occurrence. 1st Edition ed: Oxford University Press; 2003. [↑](#endnote-ref-228)
229. Baron RM, Kenny DA. The moderator-mediator variable distinction in social psychological research: conceptual, strategic, and statistical considerations. Journal of Personality and Social Psychology. 1986;51(6):1173-1182. [↑](#endnote-ref-229)
230. Kraemer HC, Wilson GT, Fairburn CG, Agras WS. Mediators and moderators of treatment effects in randomized clinical trials. Archives of General Psychiatry. 2002;59(10):877-883. [↑](#endnote-ref-230)
231. Kraemer HC, Stice E, Kazdin A, Offord D, Kupfer D. How do risk factors work together? Mediators, moderators, and independent, overlapping, and proxy risk factors. American Journal of Psychiatry. 2001;158(6):848-856. [↑](#endnote-ref-231)
232. Morgenstern J, Blanchard KA, Kahler C, Barbosa KM, McCrady BS, McVeigh KH. Testing mechanisms of action for intensive case management. Addiction. 2008;103(3):469-477. [↑](#endnote-ref-232)
233. Morgenstern J, Frey RM, McCrady BS, Labouvie E, Neighbors CJ. Examining mediators of change in traditional chemical dependency treatment. J Stud Alcohol. 1996;57(1):53-64. [↑](#endnote-ref-233)
234. Shadish WR, Cook TD, Campbell DT. Experimental and Quasi-Experimental Designs for Generalized Causal Inference. New York: Houghton Mifflin; 2002. [↑](#endnote-ref-234)
235. Copas AJ, Lewis JJ, Thompson JA, Davey C, Baio G, Hargreaves JR. Designing a stepped wedge trial: three main designs, carry-over effects and randomisation approaches. Trials. 2015;16:352. [↑](#endnote-ref-235)
236. Hughes JP, Granston TS, Heagerty PJ. Current issues in the design and analysis of stepped wedge trials. Contemp Clin Trials. 2015;45(Pt A):55-60. [↑](#endnote-ref-236)
237. Creswell J, Plano Clark, V.L., Gutmann, M.L., & Hanson, W.E. . Advanced mixed methods research designs. In: Teddlie ATC, ed. Handbook of mixed methods research designs. Thousand Oaks, CA: Sage; 2003. [↑](#endnote-ref-237)
238. Hsieh HF, Shannon SE. Three approaches to qualitative content analysis. Qual Health Res.

     2005;15(9):1277-1288. [↑](#endnote-ref-238)
239. Smith J, Firth J. Qualitative data analysis: the framework approach. Nurse Res. 2011;18(2):52-62. [↑](#endnote-ref-239)
240. Singer JW, J.B. Applied Longitudinal Data Analysis: Modeling change and event occurance New York:

     Oxford University Press 2003. [↑](#endnote-ref-240)
241. PASS 2022 Power Analysis and Sample Size Software [computer program]. Kaysville, Utah, USA:

     NCSS, LLC; 2022. [↑](#endnote-ref-241)
242. Palinkas LA, Horwitz SM, Green CA, Wisdom JP, Duan N, Hoagwood K. Purposeful Sampling for Qualitative Data Collection and Analysis in Mixed Method Implementation Research. Administration and policy in mental health. 2015;42(5):533-544. [↑](#endnote-ref-242)
