# Supplementary material for: Clinics Optimizing MEthadone Take-homes for opioid use disorder (COMET): Protocol for a stepped-wedge randomized trial to facilitate clinic level changes
Source: PLoS One. 2023 Jun 9;18(6):e0286859. doi: 10.1371/journal.pone.0286859 (PMC10256218; doi:10.1371/journal.pone.0286859)
Supplement: Peer review history

# Editor report: Decision Letter 0

**DOI:** 10.1371/journal.pone.0286859.r001

**Authors:** James Mockridge

25 May 2023

Changing and Optimizing MEthadone Take-homes for opioid use disorder (COMET): Protocol for a stepped-wedge randomized trial to facilitate clinic level changes

PONE-D-23-10287

Dear Dr. Neighbors,

We’re pleased to inform you that your manuscript has been judged scientifically suitable for publication and will be formally accepted for publication once it meets all outstanding technical requirements.

Within one week, you’ll receive an e-mail detailing the required amendments. When these have been addressed, you’ll receive a formal acceptance letter and your manuscript will be scheduled for publication.

An invoice for payment will follow shortly after the formal acceptance. To ensure an efficient process, please log into Editorial Manager at http://www.editorialmanager.com/pone/, click the 'Update My Information' link at the top of the page, and double check that your user information is up-to-date. If you have any billing related questions, please contact our Author Billing department directly at authorbilling@plos.org.

If your institution or institutions have a press office, please notify them about your upcoming paper to help maximize its impact. If they’ll be preparing press materials, please inform our press team as soon as possible -- no later than 48 hours after receiving the formal acceptance. Your manuscript will remain under strict press embargo until 2 pm Eastern Time on the date of publication. For more information, please contact onepress@plos.org.

Kind regards,

James Mockridge

Staff Editor

PLOS ONE

# Editor report: Acceptance letter

**DOI:** 10.1371/journal.pone.0286859.r002

**Authors:** James Mockridge

1 Jun 2023

PONE-D-23-10287 

Clinics Optimizing MEthadone Take-homes for opioid use disorder (COMET): Protocol for a stepped-wedge randomized trial to facilitate clinic level changes 

Dear Dr. Neighbors:

I'm pleased to inform you that your manuscript has been deemed suitable for publication in PLOS ONE. Congratulations! Your manuscript is now with our production department. 

If your institution or institutions have a press office, please let them know about your upcoming paper now to help maximize its impact. If they'll be preparing press materials, please inform our press team within the next 48 hours. Your manuscript will remain under strict press embargo until 2 pm Eastern Time on the date of publication. For more information please contact onepress@plos.org.

If we can help with anything else, please email us at plosone@plos.org. 

Thank you for submitting your work to PLOS ONE and supporting open access. 

Kind regards, 

PLOS ONE Editorial Office Staff

on behalf of

Dr James Mockridge 

Staff Editor

PLOS ONE
